# Diverse marine *Vibrio* species convert methylphosphonate to methane

**DOI:** 10.1007/s42995-025-00278-w

**Published:** 2025-02-20

**Authors:** Shu-Xian Yu, Xiaolei Wang, Yan Wang, Haonan Wang, Jiwen Liu, Wen Hong, Yunhui Zhang, Min Yu, Gui-Ling Zhang, Fabiano Thompson, Xiao-Hua Zhang

**Affiliations:** 1 Frontiers Science Center for Deep Ocean Multispheres and Earth System, and College of Marine Life Sciences, Ocean University of China, Qingdao, 266003 China; 2 Laboratory for Marine Ecology and Environmental Science, Qingdao Marine Science and Technology Center, Qingdao, 266237 China; 3 Key Laboratory of Marine Chemistry Theory and Technology, Ministry of Education/Institute for Advanced Ocean Study, Ocean University of China, Qingdao, 266100 China; 4 Key Laboratory of Evolution and Marine Biodiversity (Ministry of Education), Institute of Evolution and Marine Biodiversity, Ocean University of China, Qingdao, 266003 China; 5 Institute of Biology and Coppe, Federal University of Rio de Janeiro (UFRJ), Rio, 21941-599 Brazil

**Keywords:** Methylphosphonate demethylation, Aerobic methane production, Marine *Vibrio* strains, *Phn* operon

## Abstract

Microbial degradation of methylphosphonate (MPn) is an important pathway contributing to the ‘methane paradox’ in the oxic ocean. *Vibrio* spp. are suggested to participate in this process. However, little is known about the molecular basis, phylogenetic breadth and catabolic efficiency of methane production in *Vibrio* species. Here, 18 *Vibrionales* strains known to be effective in MPn demethylation were obtained. The most effective strains, i.e., *Vibrio gallaecicus* HW2-07 and HW2-08, can convert 70%–80% of amended MPn into methane in 5 days. Estimations based on quantitative PCR determination indicated that *Vibrio* spp. were influential contributors to marine methane production. Genes flanking the common *phn* genes suggested a divergent gene arrangement and grouped the *phn* operons into nine types. This was consistent with the phylogeny of *phnJ* and *phnL*. The *phn* operons of cluster I and II were identified frequently in *Vibrio* isolates and were common in coastal seas and the open ocean. Addition of MPn increased expression of the *phn* genes, as well as an unexpected gene that encodes an acyltransferase (*act*), which frequently occurred in cluster I–IV operons. This study provided experimental evidence and theoretical support for a further understanding that *Vibrio* spp. may play important roles in aerobic marine methane production.

**Supplementary Information:**

The online version contains supplementary material available at 10.1007/s42995-025-00278-w.

## Introduction

Methane (CH_4_) is a potent greenhouse gas, second only to carbon dioxide, that plays important roles in global warming and ozone destruction (Reeburgh 2007). Currently, the ocean is considered an important source of atmospheric methane, contributing around 1–4% of the global methane budget through sea-air exchange (Karl et al. 2008; Reeburgh 2007; Ye et al. 2020). It is widely thought that biologic methane production is mainly mediated by strict anaerobic archaea (Reeburgh 2007; Ulrich et al. 2018). All methanogens are obligate methane-producers that obtain all or most of their energy from methanogenesis, producing methane as the end-product of their anaerobic respiration (Liu and Whitman 2008). Three types of methanogenic pathways are known: CO_2_-reduction that can reduce CO_2_ to methane with H_2_ as the primary electron donor, methyl-group dismutation and, the aceticlastic reaction, which splits acetate, oxidizing the carboxyl-group to CO_2_ and reducing the methyl group to CH_4_. In methyl-group dismutation, the methylated compounds (methanol, methylated amines and methylated sulfides) are transferred to a cognate corrinoid protein and then to CoM (Coenzyme M). Subsequently, methyl–CoM enters the methanogenesis pathway and is reduced to CH_4_ (Liu and Whitman 2008). However, methane supersaturation, with respect to the atmosphere, in oxygen-rich ocean waters has been widely observed (Hilt et al. 2022; Sun et al. 2018; Ye et al. 2016), presenting the ‘oceanic methane paradox’ (Kiene 1991). Other methanogenic pathways may exist that explain the oceanic methane paradox, such as degradation of methylphosphonate (MPn) and dimethylsulfoniopropionate (DMSP) (Ye et al. 2020). Repeta et al. (2016) determined that MPn was one of the most important dissolved organic compounds in surface seawater of the North Pacific Ocean. Several abundant groups of marine microorganisms, including SAR11 (Born et al. 2017) and *Thaumarchaetoa* (Metcalf et al. 2012), synthesized MPn and the key gene *mpnS* was found to be widely distributed in marine microbes. These findings suggest substantial biotic production of MPn in marine environments, but the active MPn consumers and their potential contribution to methane accumulation in oxic seawater remain unknown.

Marine microbes, such as *Trichodesmium erythraeum* IMS 101, can degrade MPn to mitigate their phosphorus (P) limitation, and release methane into seawater (Karl et al. 2008). Several marine bacterial groups were considered to demethylate MPn and to contribute to methane production in oxic seawater, including *Pelagibacterales*, *Pseudomonas*, *Sulfitobacter*, *Rhodobacterales* and *Vibrionales* spp. (Carini et al. 2014; Martinez et al. 2013; Repeta et al. 2016; Sosa et al. 2017; Ye et al. 2020). Recently, several *Vibrio* spp. were observed to thrive in response to the addition of MPn during seawater incubation experiments; these included *Vibrio nigripulchritudo* (Martinez et al. 2013) and *Vibrio atlanticus* (Ye et al. 2020). These *Vibrio* species are important and ubiquitous heterotrophic bacteria, sharing the common characteristics of halophilism, short generation times and a range of metabolic capabilities (Thompson et al. 2006; Zhang et al. 2018). These organisms can utilize a diverse range of organic carbon compounds in the marine environment, including chitin, alginic acid, agar, laminarin and fucoidan (Farmer et al. 2005; Zhang et al. 2018), and immediately respond to nutrient enrichment through rapid growth (Liang et al. 2021). Although *Vibrio* spp. usually comprise up to ~ 1% of the bacterioplankton community in coastal waters, they can thrive to become the dominant members of the bacterial population during algal blooms or micronutrient input (Thompson and Polz 2006; Wang et al. 2020; Zhang et al. 2018). Furthermore, ongoing ocean warming also favors the global spread of *Vibrio* spp. (Vezzulli et al. 2012), causing an increase in marine carbon and phosphorus cycling. *Vibrio* spp. may be among the most important bacterial groups executing MPn demethylation and methane production in oxic oceans.

The ability bacteria to demethylate MPn into methane via the *phn* operon (Ulrich et al. 2018) was first identified in *Escherichia coli* (Chen et al. 1990; Metcalf and Wanner 1993a, b). The *phn* operon in *E. coli* contains 14 functional genes (*phnCDEFGHIJKLMNOP*) encoding the C-P lyase pathway, which catabolizes a broad suite of phosphonates (e.g., MPn, 2-aminoethylphosphonic acid). *phnCDE* encodes the specific transporter of phosphonates; *phnGHIJKLM* encodes the basic catalytic unit to break the C-P bond and *phnFNOP* encodes regulatory or related necessary proteins (Martinez et al. 2013). In the presence of PhnG, PhnH and PhnL, PhnI is required to catalyze the nucleophilic attack of methylphosphonate on the anomeric carbon of MgATP to form adenine and α-D-ribose-1-methylphosphonate-5-triphosphate (RPnTP). Then, PhnM releases pyrophosphate from the resulting 5’-phosphoribosyl-α-1-phosphonate to allow PhnJ to cleave the C–P bond via a S-adenosyl methionine (SAM)-dependent glycine-radical reaction mechanism and release CH_4_. Finally, the combined action of PhnP and PhnN converts the resulting cyclic ribose into 5-phosphoribosyl-α-1-diphosphate (PRPP) (Amstrup et al. 2023; Kamat et al. 2013). Among these genes, *phnJ* is strictly conserved and encodes a protein, PhnJ, directly involved in methane production (Kamat et al. 2013; Sosa et al. 2019). In addition, gene composition and structure of the *phn* operon varies greatly among bacterial species (Huang et al. 2005; Stosiek et al. 2020). In contrast with *E. coli*, *V. nigripulchritudo* ATCC 27043 has a *phn* operon containing 13 genes, *phnCDEFGHIJKLMNP*, without *phnO* (Martinez et al. 2013), whereas the *phn* operons in other *Vibrio* species, such as *V. atlanticus*, remain unknown. Further investigations into the structural and functional diversity of *phn* operons in *Vibrio* spp. are needed to better understand their roles in the process of MPn demethylation.

The aims of this study were to explore the diversity of *phn* operons in *Vibrio* spp., to confirm the abundance of MPn-demethylating *Vibrio* strains in natural environments and to determine their potential contributions to marine methane production. In this study, MPn-incubation experiments were conducted and methane measurements made, to screen for MPn-demethylating *Vibrio* strains. Genomic and transcriptomic analyses were further performed to find the related genes responsible for MPn demethylation process in *Vibrio* species. Also, bioinformatic and quantitative analyses were conducted to explore the distribution and abundance of key *phn* genes.

## Materials and methods

### Isolation and purification of *Vibrio* strains

Coastal seawater was collected from the Zhanqiao pier area of Qingdao (ZQ; 120.377°E, 36.04°N) in May of 2020, May and June of 2021. Enrichment experiments of MPn-demethylating microbes were then undertaken following (Martinez et al. 2013; Ye et al. 2020). Sixty mL of seawater was transferred into 100-mL sterile vials, amended with glucose (C), nitrate (N) and MPn at a final concentration of 1000 μmol L^−1^, 160 μmol L^−1^ and 10 μmol L^−1^, respectively, and incubated at 28 °C in a shaker for 5 days; this comprised the seawater-MPn-incubation system. During incubation, diluted samples were regularly spread onto the surface of marine 2216E agar (MA; 10^–3^–10^–5^) and TCBS (thiosulfate citrate bile salts sucrose agar; 10^–1^–10^–3^) plates to retrieve MPn-demethylating *Vibrio* cultures. Single cultures, isolated from the seawater-MPn-incubation system, were purified at least three times using the plate streaking method on MA plates prior to identification of 16S rRNA gene sequences using the EzBioCloud database (Yoon et al. 2017b). Several *Vibrio* strains were retrieved from the − 80 °C glycerol stock (15%) in our laboratory, and purified on MA plates for subsequent CH_4_ determination.

### *Vibrio*-MPn incubation and CH_4_ measurement

The CH_4_ production of *Vibrio* strains was measured to determine their MPn-demethylating ability. Acid-washed headspace vials, containing 60 mL of liquid medium and 40 mL of headspace, were used in the incubation experiments. Each *Vibrio* strain was washed clear of MA medium, inoculated into 60-mL MPn medium (1:100), and then incubated at 28 °C for 5 days as previously described (Ye et al. 2020). The MPn medium contained low-phosphate seawater (Pi < 0.02 μmol L^−1^) amended with 1000 μmol L^−1^ C, 160 μmol L^−1^ N and 10 μmol L^−1^ MPn. After the 5 days incubation, 2 mL of gas was extracted from the headspace using a gas-tight syringe (VICI, Baton Rouge, USA), and then measured on a gas chromatograph (Shimadzu GC-14B) equipped with a flame ionization detector (FID). The dissolved CH_4_ concentrations were calculated using the Bunsen coefficient (Ruppel and Kessler 2017). To confirm the MPn-demethylating ability of *Vibrio* spp., incubations of *V. gallaecicus* HW2-07 with MPn as the sole carbon and phosphorus sources, a series of glucose concentrations (10, 100, 200, 500 and 1000 μmol L^−1^) and a series of MPn concentrations (0.001, 0.01, 0.1, 1 and 10 μmol L^−1^) were carried out.

During the incubations, the growth of *Vibrio* spp. was measured using spectrophotometry at 600 nm. An additional 1 mL from each sample was stained with SYBR Green and the cells were counted using an influx flow cytometer (Beckman-FC-500) as previously described (Ye et al. 2020). All the experiments were conducted with three replicates.

### Genome sequencing and analysis

Each *Vibrio* strain was maintained and cultured on MA medium, and washed with 0.85% sodium chloride solution prior to centrifugation at 10,000 rpm for 15 min. Genomic DNA was extracted using a Blood & Cell Culture DNA Midi Kit (Qiagen, USA) according to the manufacturers protocol, and then sequenced on the MGISEQ-2000 platform and PacBio Sequel II system at BGI (Shenzhen, China). Clean reads were assembled by Unicycler v 0.4.8 (Wick et al. 2017).

Assembled genomes were annotated with the Rapid Annotation using Subsystem Technology (RAST) pipeline (Aziz et al. 2008), and further annotation was performed against the NCBI non-redundant proteins (NR) database (Pruitt et al. 2005), clusters of orthologous groups of proteins (COG) database (Tatusov et al. 2000), Kyoto encyclopedia of genes and genomes (KEGG) database (Kanehisa et al. 2015), gene ontology (GO) database (Ashburner et al. 2000) and swiss-prot database (Consortium 2020). The PHASTER (Arndt et al. 2016) web server was used for the rapid identification and annotation of prophage sequences within *Vibrio* genomes and plasmids. To determine the definitive species of sequenced *Vibrio* strains, the genome distance, based on the average nucleotide identity (ANI) (Yoon et al. 2017a) and DNA-DNA hybridization (DDH) (Meier-Kolthoff et al. 2013), was calculated. The online tool IslandViewer 4 was used to predict the genomic regions (genomic islands) with abnormal sequence composition that was horizontally transferred (Bertelli et al. 2017).

Based on the valid *Vibrio* species records of LPSN (https://lpsn.dsmz.de/), 105 complete genomes were selected from the NCBI database and the sequenced genomes to perform the core genome phylogenetic analysis, as previously described (Lin et al. 2018). In addition, the *phn* operons in 101 non-complete *Vibrio* genomes were confirmed through a protein reference sequence search and conversed domain (CD) identification in NCBI. The *phn* gene sequences from *Vibrio* genomes were used to perform phylogenetic analysis and local database construction. The maximum-likelihood (ML) trees of the *phn* gene sequences were constructed using MEGA v10.2.2 (Kumar et al. 2018). In addition, the clades of *Vibrio* spp. were classified according to a previous standard (Jiang et al. 2022).

### Transcriptome sequencing and analysis

*Vibrio* strains were pre-induced in 10 mL MPn medium for 24 h prior to mass culturing. Triple treatment samples were setup in Erlenmeyer flasks with MPn medium (MPn-incubation samples) and MPn medium with phosphate (MPn + Pho incubation samples). The control samples were subject to the same concentrations of C, N and phosphate (Pi = 10 μmol L^−1^, phosphate incubation samples). The phosphate solution (pH 7.6) was made using 10 μmol L^−1^ NaH_2_PO_4_ and 10-μmol L^−1^ Na_2_HPO_4_. After incubation for 16–20 h (the latent period of the logarithmic phase), cultures were harvested by centrifugation at 10,000 rpm for 5 min, then frozen in liquid nitrogen, and finally stored at − 80 °C. The RNA library was prepared using an Illumina TruSeq™ RNA Sample Prep Kit method, and transcriptome sequencing were performed at Majorbio (Shanghai, China). Clean data mapping was conducted using the Bowtie program with Burrows-Wheeler method (Langmead et al. 2009). Quantitative analysis of gene expressions was performed using RSEM (RNA-Seq by Expectation–Maximization) with the TPM (Transcripts Per Million reads) index (Li and Dewey 2011). Operons of transcripts was predicted using Rockhopper (Tjaden 2020). Differentially expressed genes (DEGs) were obtained based on the normalized FPKM (Fragments Per Kilobase of transcript per Million mapped reads) values in alginate- and glucose-treated samples. False Discovery Rate (FDR) control (Trapnell et al. 2010) was used to correct for the P-value. Genes with an FDR value ≤ 0.05 and |logFC|≥ 1 were assigned as differentially expressed. Differences in gene transcription (i.e., up-regulated and down-regulated genes) were analyzed with a one-way ANOVA.

### *Vibrio*-specific *phnJ* and *phnL* primers design

*phnJ* and *phnL* have been successfully used in the PCR-dependent study of Wang et al., (2017). Here sequences of *phnJ* and *phnL* from the *Vibrio* genomic data were used to design *Vibrio*-specific primers. CLUSTALW (Thompson et al. 1994) and the online program GeneFisher 2 (Giegerich et al. 1996) were used to align the *phnJ*/*L* sequences and calculate their consensus sequences. Several pairs of degenerate primers were designed to cover the conversed regions on the *phnJ* or *phnL* sequences (Table S1). PCR was conducted to verify the accuracy of these primers using the *Vibrio* strains that already possess the complete genome data and the bacterial strains isolated from the seawater-MPn-incubation system. The combination of *phnJ*-94F/895R and *phnL*-63F/672R was able to generate long products of *phnJ* and *phnL* (Table S2), whereas *phnJ*-620F and *phnJ*-895R target the CD containing four cysteine residues of the PhnJ sequence, and were able to identify the *phnJ* gene in all CH_4_-producing strains. The primers *phnL*-370F and *phnL*-672R were also very effective in *phnL* gene detection in most *Vibrio* strains.

### High-throughput sequencing and qPCR using *Vibrio*-specific primers

Sea water samples were collected from the coast near ZQ by filtering 500-mL seawater through 0.22-μm polycarbonate membranes (GTTP, 47 mm, Millipore). Total DNA was extracted using the DNeasy PowerSoil Kit (Qiagen, USA), according to the protocol. The concentration and quality of the DNA extracts were determined by a NanoDrop 2000 spectrophotometer (ThermoFisher, USA) and agarose gel electrophoresis. High-throughput sequencing was performed at Majorbio (Shanghai, China) using the 300-bp PE Illumina MiSeq sequencing platform with primers *phnJ*-620F/895R and *phnL*-370F/672R to estimate the gene abundance. OTUs were generated with 97% similarity, and annotated against the NCBI nucleotide database (NT_v20200604) and the local *phnJ*/*phnL* databases.

Oceanic seawater was collected from the northwestern Pacific Ocean (NPO) and incubated with 100 μmol L^−1^ C, 16 μmol L^−1^ N and 1 μmol L^−1^ MPn. The seawater samples were filtered using 0.22-μm polycarbonate membranes at 0 h (NPO) and 72 h (NPO-MPn). The abundance of the gene *phnL* was quantified by a SYBR Green qPCR method using the primers *phnL*-370F and *phnL*-672R (Table S1). The reactions were performed in triplicate according to the following profile: an initial denaturation at 95 °C for 2 min, followed by 40 cycles of a 3-step reaction at 95 °C for 20 s, 53 °C for 20 s and 72 °C for 40 s. The 20-μL qPCR system involved 10 μL 2 × SYBR Premix Ex Taq II, 0.4 μL 50 × ROX Reference Dye II, 0.8-μL primers (10 μM), 6-μL sterile double-distilled water and 2-μL DNA samples. The total *Vibrio* abundance was determined as previously using the *Vibrio*-specific primers (Wang et al. 2020). All qPCR assays were performed using an Applied Biosystems QuantStudio® 5 Real-Time PCR System in triplicate, and the efficiencies of the qPCR reactions varied from 95 to 105%, with R^2^ values > 99%.

### Analysis of *phnJ*/*L* genes in Tara Ocean dataset

Based on the assigned KEGG identifiers, the *phnJ* (K06163) and *phnL* (K05780) genes were queried in the Ocean Microbial Reference Gene Catalogue (OM-RGC) dataset (http://ocean-microbiome.embl.de/companion.html) from the *Tara Ocean* expedition (Sunagawa et al. 2015). These *phnJ*/*L* sequence records were analyzed against the nucleotide database of NCBI and the local database of *phn* genes in order to find the *phnJ*/*L* sequences belonging to *Vibrio*. The single copy gene *recA* (K03553) was used as a reference gene to normalize the abundance of *phnJ*/*L* and uncover their global distribution and diversity.

## Results

### CH_4_ production by *Vibrio* strains

**Fig. 1:**
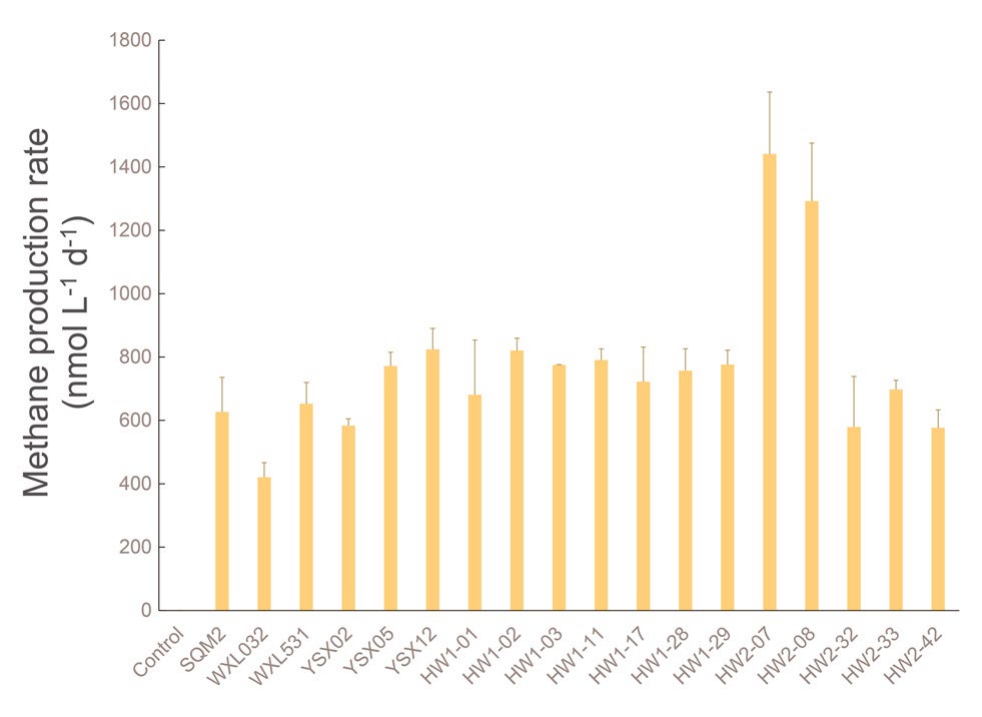
The daily methane production of *Vibrio* strains demethylating MPn (10 μmol L^−1^). The error bars present 1 standard deviation (SD) of at least triplicate samples

In this study, CH_4_-producing capability of 46 *Vibrionales* isolates, which were affiliated with at least 17 species (Table S2), were examined. Of these, 18 strains from 12 *Vibrio* species were able to demethylate MPn into CH_4_ (Fig. 1 and Table S2). Incubation with MPn led to rapid cell growth of *Vibrio* species during the first 2–3 days (Figs. S1 and S2) and methane release was evident within 5 days (Fig. 1). *Vibrio gallaecicus* HW2-07 and HW2-08 were relatively more efficient in MPn demethylation than other strains, producing 1441.0 ± 195.6 nmol L^−1^ and 1292.3 ± 182.7 nmol L^−1^ CH_4_ per day, respectively. *Vibrio cyclitrophicus* WXL032 showed the weakest MPn-demethylating capability among all strains, releasing CH_4_ at an average rate of 420.4 ± 46.3 nmol L^−1^ d^−1^. The CH_4_ production rates of the other strains ranged from 576.7 ± 56.5 to 824.4 ± 65.8 nmol L^−1^ d^−1^. Thus, it was calculated that most of the *Vibrio* strains demethylated approximately 23%–45% of the amended MPn (10 μmol L^−1^) after incubation for 5-days, whereas highly efficient *V. gallaecicus* strain HW2-07 and HW2-08 could convert 74% and 82%, respectively, approaching the efficiency of *E. coli* BL21 (Fig. S3). Several none-*Vibrio* strains were also isolated from the seawater-MPn-incubation system, including six *Rhodobacterales* strains, one each of *Oceanospirillales* and *Sphingomonadales* (Table S3). Those none-*Vibrio* strains were only able to convert 0.2%–3.9% of amended MPn during the 5-day incubation period (Fig. S4).

To confirm the MPn-demethylating ability of *V. gallaecicus* HW2-07, MPn was added as the sole carbon and phosphorus sources to the incubation. The results show that methane was present in the incubation system of strain HW2-07 (Fig. S5A). Incubation of HW2-07, under a series of glucose concentrations, showed that a high concentration of glucose can affect methane production. However, when the glucose concentration was greater than 200 μM, its influence on methane production had only a limited effect (Fig. S5B). Incubation of HW2-07 under a series of MPn concentrations found that methane production was low in the low MPn groups, although this may be attributed to the inaccurate detection method (Fig. S5C).

### Diversity of the *phn* operons in *Vibrio *spp.

Six of the MPn-demethylating strains, including *V. mediterranei* QT6D1 (WXL531), *V. cyclitrophicus* WXL032, *V. gigantis* SQM2, *V. pomeroyi* YSX02, *V. chagasii* YSX05 and *V. gigantis* YSX12, were verified previously than the other strains which were listed in the Table S2, and the complete genomes were sequenced. The *phn* operon was identified in the genomes of all six strains. The *phn* operon in *V. mediterranei* WXL531 contains ten concatenated genes *phnFGHIJKLMNP* whereas phnCDEE are located separately. Another five strains share the identical *phn* operon, including 13 linked genes *phnCDEFGHIJKLMNP*, suggesting diverse types of *phn* operons in *Vibrio* strains.

**Fig. 2:**
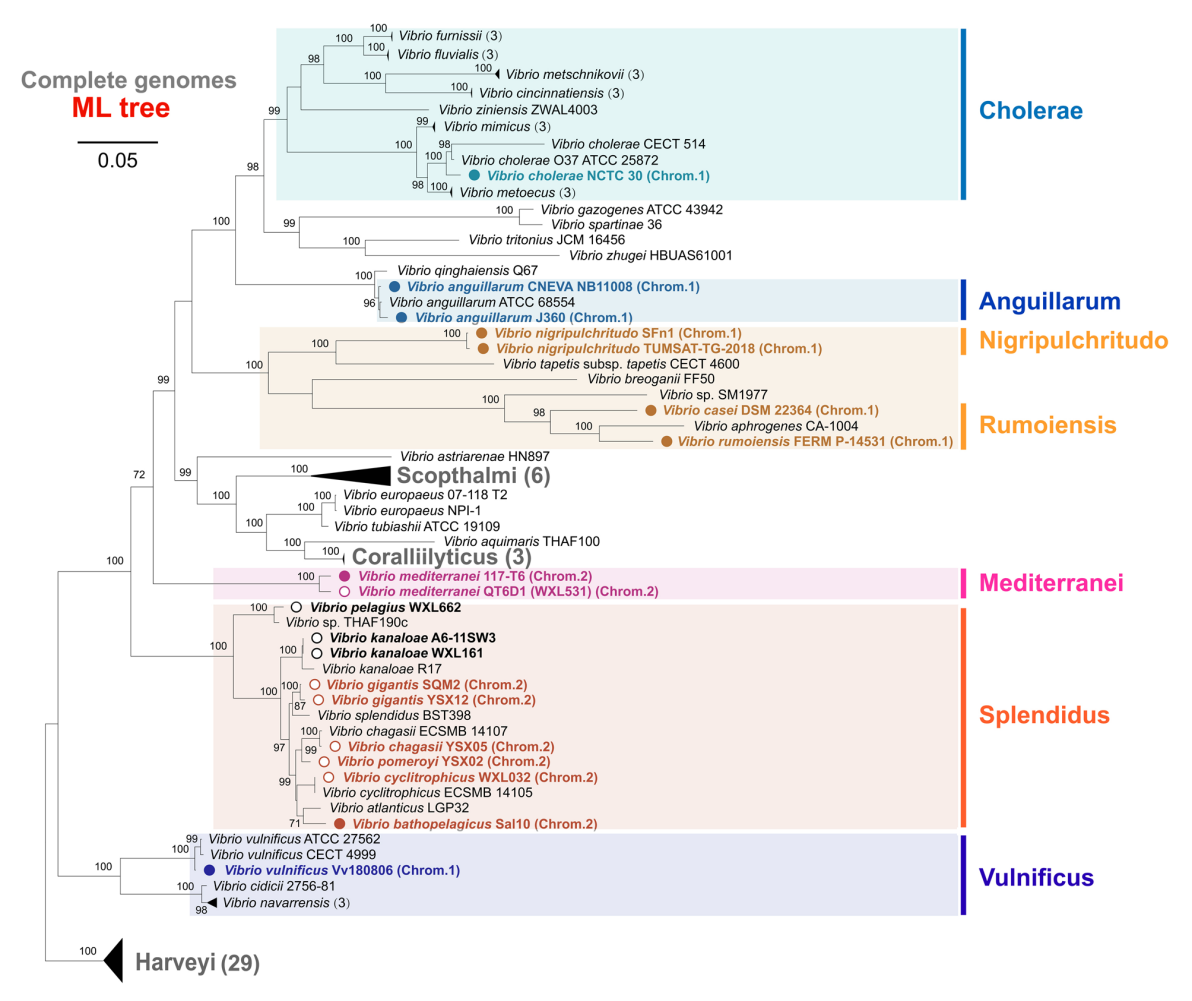
Phylogenetic analysis of the genus *Vibrio* based on the maximum-likelihood tree of core genomic protein sequences from 105 complete genomes. The defined clades that do not contain *phn* operons in genomes were collapsed in triangles, and the number of complete genomes included in the collapsed branches is indicated in the parentheses. Genomes that contain *phnG* ~ *phnK* were labeled with blue dots, whereas those genomes include at least *phnG* ~ *phnP* were colored by orange. Location of these *phn* operons in the larger chromosome (Chr. 1) or the smaller one (Chr. 2) is indicated in the parentheses. Genomes from our laboratory were marked with the circle markers

Over 5000 genomic assemblies of *Vibrio* spp., available in the NCBI database, and corresponding to 133 *Vibrio* species with validly published names in the LPSN database (Table S4), were investigated. Of these, 116 *phn* operons, belonging to 12 *Vibrio* clades and 32 *Vibrio* species, were identified. Eleven representative *phn* operons were identified from 345 complete *Vibrio* genomes (Table S4) and these were mainly affiliated with the *Vibrio* clades Cholerae, Anguillarum, Nigripulchritudo, Rumoiensis, Mediterranei, Splendidus and Vulnificus (Fig. 2). Based on the gene composition, these *phn* operons could be roughly classified into two groups. One group contained five *phn* operons with only *phnGHIJK* that was located in the larger chromosome (Chr. 1). These were affiliated with the clades Cholerae, Anguillarum, Rumoiensis and Vulnificus. The other group (six operons) possessed at least the critical genes *phnG* ~ *phnP* in their operons, and belonged to the clades Nigripulchritudo, Rumoiensis, Mediterranei and Splendidus. Both two types of the *phn* operons occurred in *V. casei* DSM 22364 (clade Rumoiensis). All six MPn-demethylating strains validated in this study belonged to the latter group. The *phn* operons of clades Nigripulchritudo and Rumoiensis were in Chr. 1, whereas those of Mediterranei and Splendidus existed in the smaller chromosome (Chr. 2). According to the complete assemblies, not all *Vibrio* spp. possessed the *phn* operons. Even different strains of the same species were not conserved in the presence of the *phn* operon, such as *V. cyclitrophicus* WXL032 (+) and *V. cyclitrophicus* ECSMB 14105 (−). This may well suggest that the *phn* operons in *Vibrio* spp. were originally gained via horizontal gene transfer (HGT).

**Fig. 3:**
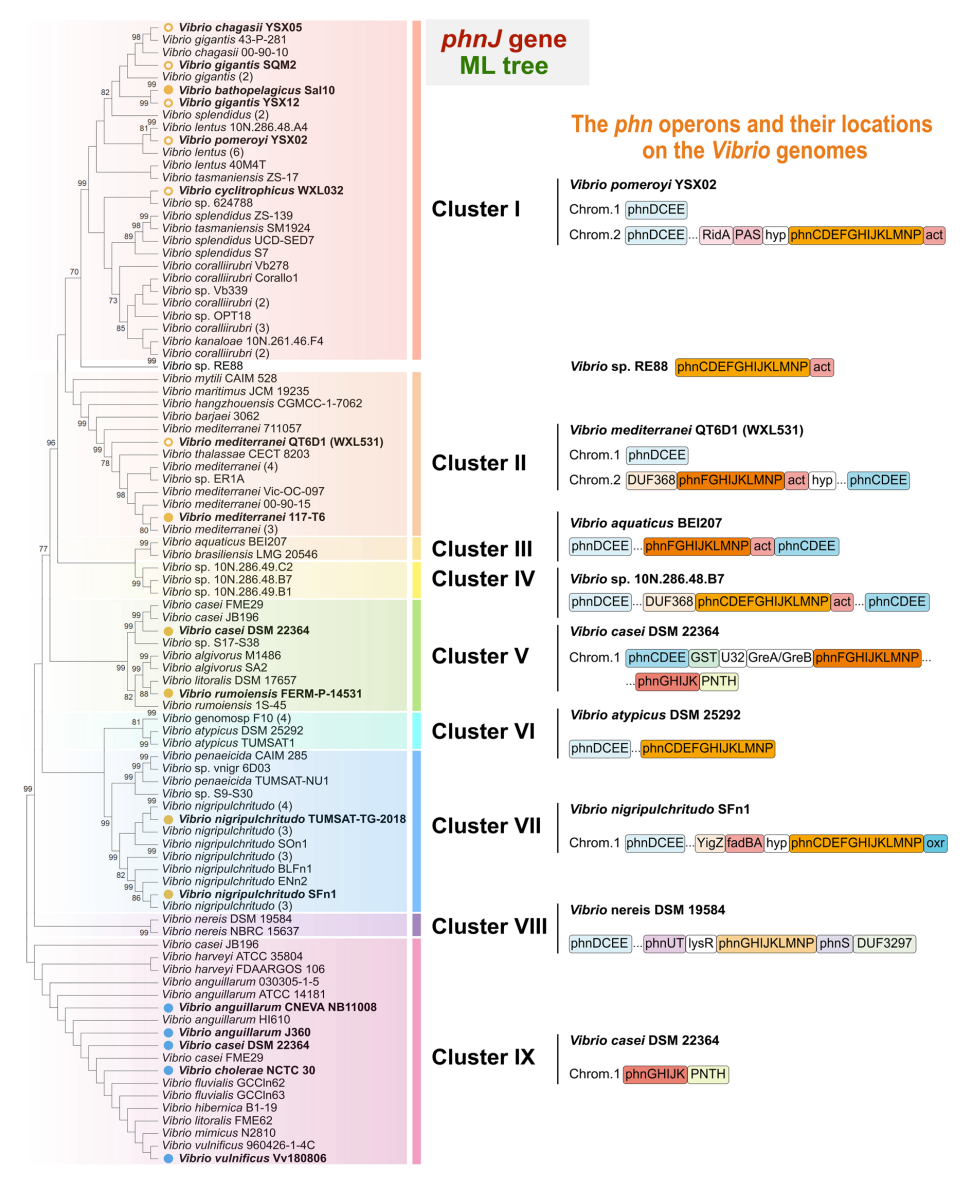
Phylogenetic analysis of *phnJ* sequences from 116 *Vibrio* genomes and their related *phn* operons. The bootstrap values ≥ 70% were shown in the phylogenetic tree. *phnJ* sequences from NCBI complete genomes were marked with blue or orange dots, whereas those sequences from our laboratory were labeled with orange circles. Genomes that contain *phnG* ~ *phnK* were labeled in blue dots, whereas those genomes include at least *phnG* ~ *phnP* were colored by orange. The branches of the same species were folded as much as possible, and their quantity was displayed in the parentheses. RidA, reactive intermediate/imine deaminase A family protein; PAS, PAS domain-containing protein; hyp, hypothetical protein; act, acetyltransferase; GST, glutathione S-transferase; GreA/GreB, GreA/GreB family transcription elongation factor; PNTH, P-loop containing nucleoside triphosphate hydrolases; fadBA, fatty acid oxidation complex subunit alpha FadB and acetyl-CoA C-acyltransferase FadA; oxr, oxidoreductase

The composition and location (including upstream and downstream genes) of the *phn* operons in *Vibrio* genomes were classified into nine clusters according to their differential gene arrangement (Fig. 3). The topologies of *phnJ* (Fig. 3) and *phnL* (Fig. S6) trees, based on the ML approach, were very similar and the *phn* operons divided both into nine clusters, indicating the synchronized evolution of the single gene (*phnJ* or *phnL*) and their complete operon. The *phn* operons of cluster I and II accounted for nearly 50% (56 *Vibrio* spp.) of the total *Vibrio* spp. investigated in this study. Clusters I, IV, VI and VII operons of the key *phn* genes, all contained *phnC* ~ *phnP*; clusters II, III and V involved *phnF* ~ *phnP*; cluster VIII operon had *phnG* ~ *phnP*, whereas cluster IX operon included only *phnG* ~ *phnK*.

**Fig. 4:**
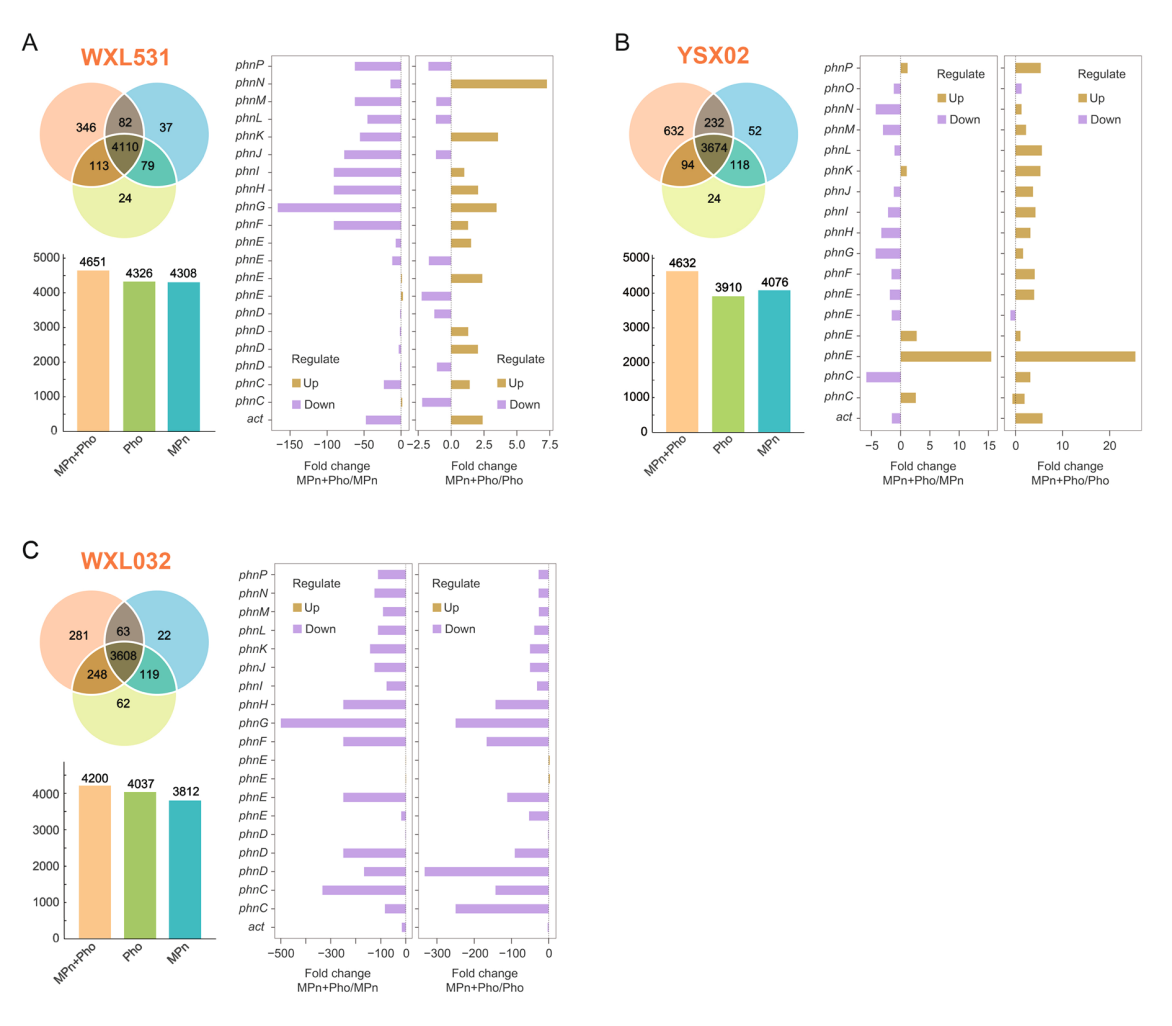
The expressed gene number and the fold change of *phn* gene expression in the *Vibrio* strains when they were separately incubated with MPn and phosphate (**A** ~ **C**). *act*, the gene encoding acetyltransferase. The genes that were significantly upregulated (ANOVA, *P* < 0.05) in the MPn-incubation system are labeled with asterisks (*) in the figure. The orange area showed the expressed gene number when vibrio demethylated MPn as the sole phosphorus source, whereas the blue area presented the gene number expressed in the phosphate incubation samples

There were two frequently up-regulated genes near the *phnC* of cluster I, encoding reactive intermediate/imine deaminase, for the neighbor genes of the *phn* operon, a family protein (RidA) and a PAS domain-containing protein (e.g., *V. pomeroyi* YSX02 in Fig. 4). Both cluster II and IV had a DUF368-encoding gene located upstream of the *phn* operon and the *phnCDEE* occurred far away from other key genes (e.g., *V. mediterranei* WXL531 and *Vibrio* sp. 10N.286.48.B7). Cluster III did not have the DUF368-encoding gene and the *phnCDEE* were close to other *phn* genes (e.g., *V. aquaticus* BEI207). All these four operon types had a gene encoding acetyltransferase (*act*) next to *phnP*. In the cluster V operon, there were four genes between *phnCDEE* and *phnF* ~ *phnP*, respectively, encoding glutathione S-transferase (GST), U32 and GreA/GreB family transcription elongation factors (e.g., *V. casei* DSM 22364). The *phn* genes in *Vibrio rumoiensis* FERM P-14531 (cluster V) were closely joined to the questionable phage sequences predicted by PHASTER. *Vibrio* spp. affiliated with cluster VII, e.g., *V. nigripulchritudo* SFn1, commonly had a gene encoding oxidoreductase (*oxr*) next to the *phn* operon. The *phn* operon of cluster VI contained *phnC* ~ *phnP* (e.g., *V. atypicus* DSM 25292), whereas *phn* genes of cluster VIII were located between *phnUT* and *phnS* (encoding 2-aminoethylphosphonate transport system) (e.g., *V. nereis* DSM 19584). The phylogenetical conservation in *phn* operon clustering and the frequent occurrence of adjacent genes may well indicate that the evolution of *phn* operons in *Vibrio* spp. occurred together with the gene rearrangement.

Most of the *Vibrio* genomes had a single copy of the *phn* operon. However, *V. casei* DSM 22364 had another type of *phn* operon that only contained genes *phnG* ~ *phnK*, representing the cluster IX operon (Fig. 3). Intriguingly, the *phnJ* sequences from cluster IX were highly homologous, and encoded exactly the same PhnJ protein (100% identity with WP_019282993.1). This *phn* operon was surrounded by insertion sequences (ISs), such as the genes encoding IS3 and IS66 family transposes in the upstream and downstream region. Consequently, the genomic region of *phnG* ~ *phnK* was predicted to be within a genomic island. In addition, *Vibrio vulnificus* Vv180806 had genes encoding inovirus Gp2 family protein and bacteriophage abortive infection AbiH family protein in the upstream region of *phn* operon, suggesting the possibility of HGT being mediated by phage induction. These findings indicate the *phn* operons of cluster IX might have the same genetic resource and be highly conserved during their dispersal among *Vibrio* spp.

### Transcription of MPn-demethylating genes in *Vibrio* isolates

Of the six *Vibrio* isolates in this study, the high MPn-demethylating strains, i.e., *V. mediterranei* WXL531, *V. pomeroyi* YSX02 and *V. cyclitrophicus* WXL032, were chosen to perform transcriptome analysis. These three strains exhibited different response patterns upon change of P resource (Fig. S1, Fig. 4A–C and Table S5). When incubated with MPn, *V. mediterranei* WXL531 grew much more rapidly than *V. cyclitrophicus* WXL032 (Fig. S2). In addition, the growth status of *V. mediterranei* WXL531 showed a similar trend in MPn- and phosphate incubation systems, whereas the growth of *V. cyclitrophicus* WXL032 in the MPn-incubation system was relatively subdued compared to that in the phosphate incubation system (Fig. S7). This suggests that *V. mediterranei* WXL531 could adapt rapidly to the Pi-limited environment with MPn.

At the transcriptional level, 42 and 11 genes of *V. mediterranei* WXL531 were significantly up-regulated or down-regulated (ANOVA, *P* < 0.05), respectively, in the MPn-incubation samples, when compared with genes in the phosphate incubation samples (Table S5). In contrast, expression of 136 and 95 genes in *V. pomeroyi* YSX02 was significantly up-regulated and down-regulated (ANOVA, *P* < 0.05), respectively, in the MPn-incubation system. For *V. cyclitrophicus* WXL032, 360 genes were repressed under incubation with MPn (Fig. 4C) and the expression of more than 860 genes was regulated (Table S5). Although the regulation of gene expression in response to P source change differed greatly among these strains, *phn* gene expressions were up-regulated in the MPn-incubation samples of all three strains (Fig. 4, Fig. S8 and Table S5). In addition, transcriptomic analyses of WXL531 and YSX02 under high MPn and high phosphate conditions showed that almost all *phn* gene expressions were up-regulated compared to the phosphate incubation group (Fig. S9). It is noteworthy that the gene *act*, beside *phnP* in cluster I–IV operons with unknown function, was also significantly up-regulated in the MPn-amended incubation (ANOVA, *P* < 0.05). Operon prediction of transcriptomes indicated that *act* may be a constant component of the *phn* operons. The *act* gene frequently occurs in the *phn* operons of many other bacteria, such as *Agrobacterium tumefaciens*, *Mesorhizobium loti*, *Sinorhizobium meliloti* and *Thermus thermophilus* (Fig. S10), and their encoding proteins shared 44%–51% identity with those in *Vibrio* spp.

### Diversity and abundance of *phnJ*/*phnL* revealed by *Vibrio*-specific primers

**Fig. 5:**
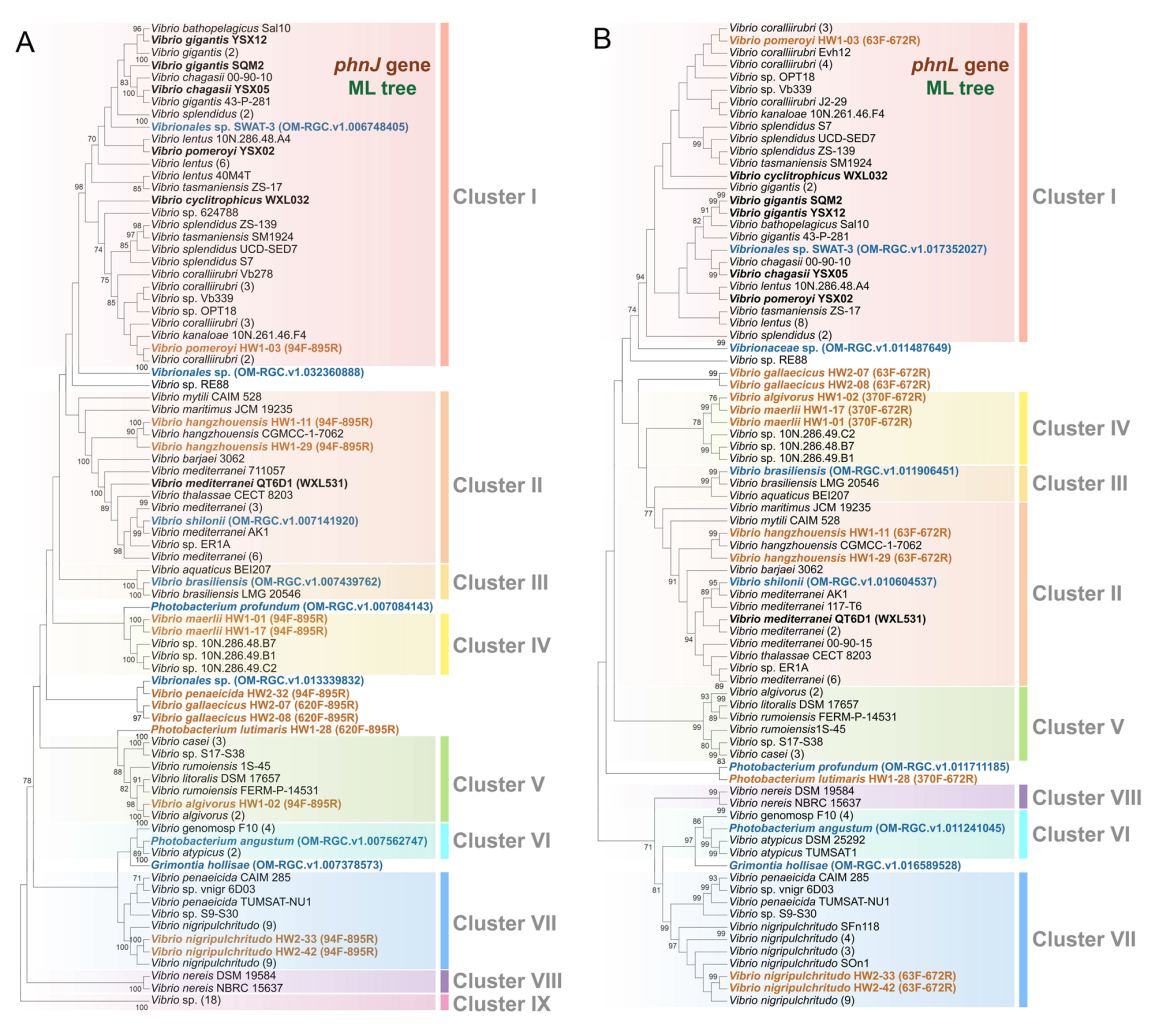
Phylogenetic analysis of the *phnJ* (**A**) and *phnL* (**B**) sequences from the *Vibrio* genomes, the isolated vibrios and *Tara Ocean* datasets. The bootstrap values ≥ 70% were shown in the phylogenetic tree. Sequences from the complete genomes of our laboratory were highlighted with bold letters, whereas those sequences from sequencing PCR products of isolated strains were colored with orange, and the primers used for the sequences were presented in parentheses. Sequences from the *Tara Ocean* datasets were colored by blue. The branches of the same species were folded as much as possible, and their quantity was displayed in the parentheses

*Vibrio*-specific primers targeting the *phnJ* and *phnL* genes (Table S1) were designed and optimized to examine the *phn* operon types in MPn-demethylating isolates. Phylogenetic analyses of *phnJ* and *phnL* indicated that all isolates were mainly affiliated with the *phn* operon clusters I, II, IV, V and VII (Fig. 5). Clusters I, II, IV, V and VII showed similar CH_4_ producing rates (ranging from 576.7 ± 56.5 to 824.4 ± 65.8 nmol L^−1^ d^−1^; Fig. 1). The highly efficient isolates, i.e., *V. gallaecicus* HW2-07 and HW2-08, were not classified into any *phn* operon cluster, indicating that new clusters may exist and even potentially novel genes involved in MPn demethylation.

High-throughput sequencing was performed to evaluate the *Vibrio*-specificity of the *phnJ* and *phnL* primers and to estimate the abundance and diversity of *phnJ* and *phnL* in the ZQ coastal sample. As a result, only 1 of 147 *phnJ* OTUs (133 of 100 144 reads) was assigned to *Vibrio* spp. (Table S6). The *phnJ* primers appeared to preferentially target *Rhodobacterales* in the coastal microbial community. This might suggest that the highly conversed *phnJ* was insufficient for *Vibrio*-specific identification in the microbial community. Unlike *phnJ*, the *Vibrio* OTUs based on *phnL* sequence homology (40 OTUs, 40 999 reads) accounted for approximately 50% of total reads (446 OTUs, 82 004 reads) and indicated that clusters I, II and V were the most abundant *phn* operon types in coastal seawater (Table S6).

The *Vibrio*-specific *phnL* primers were subsequently used to primarily quantify the abundance of MPn-demethylating *Vibrio* species in microbial communities. In the coastal sample of ZQ, the *phnL* abundance was determined as 5.2 × 10^6^ copies L^−1^. In the oceanic samples, the methane concentration was 3 nmol L^−1^ in natural NPO seawater (0 h) and the *phnL* abundance was beneath the detection limit. After addition of MPn, the methane concentration increased to 385 nmol L^−1^ in NPO-MPn (72 h) and the *phnL* abundance was determined as 3.9 × 10^6^ copies L^−1^. Correspondingly, the abundance of *Vibrio*-16S rRNA gene increased from 35 copies L^−1^ to 6.9 × 10^6^ copies L^−1^ in three days, exhibiting a rapid response to the amended nutrients.

### Diversity of *phnJ*/*phnL* in Tara Ocean data

**Fig. 6:**
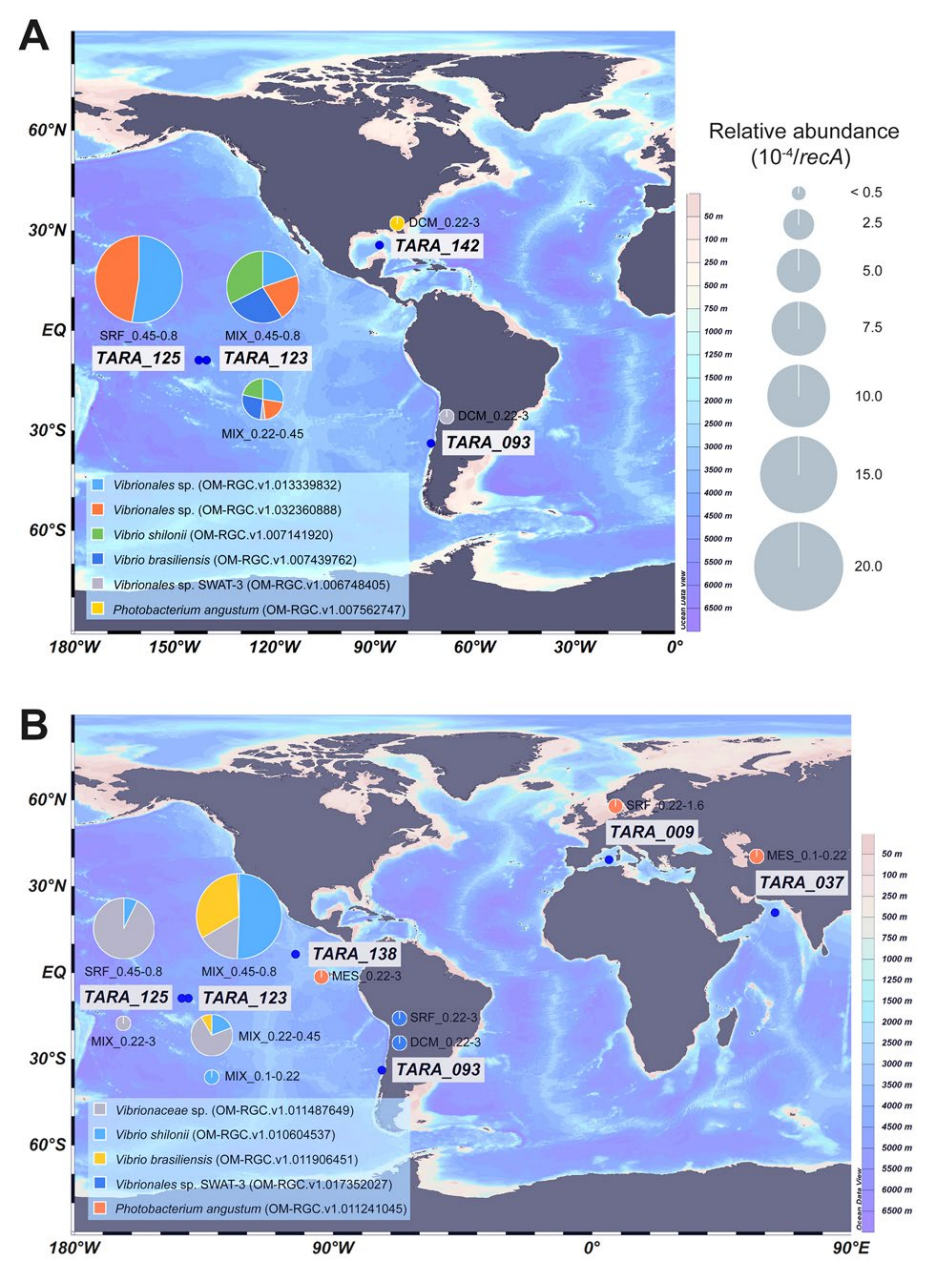
Distribution of the *Vibrionales phnJ* (**A**) and *phnL* (**B**) sequence records found in the *Tara Ocean* dataset. Stations were labeled with blue dots. The sequence IDs were presented in parentheses, and their relative proportions in the samples were shown by the pie charts. DCM, deep chlorophyll maximum layer; SRF, surface water layer; MIX, marine epipelagic mixed layer; MES, mesopelagic zone. The suffixes present the sample fractions, such as 0.22–3 means the sample was collected with the fraction size between 0.22 μm and 3 μm

A total of 285 and 253 records of *phnJ* (K06163) and *phnL* (K05780), respectively, were retrieved from the *Tara Ocean* dataset. Among these records, only eight *phnJ* and seven *phnL* sequences were assigned to *Vibrionales* and these were mainly affiliated with the *phn* operon clusters I, II, III and VI (Fig. 5). The *phnJ* and *phnL* sequences were relatively more abundant (on average > 4 × 10^–4^ normalized by *recA*) in the samples from the upper water layers (depth ≤ 150 m) of TARA_123 and TARA_125 (Fig. 6 and Table S7), including two *V. brasiliensis* sequences (cluster III), two *V. shilonii* sequences (cluster II), two *Vibrionales* sp. SWAT-3 sequences (cluster I) and another three *Vibrionales* sequences. *Vibrionales* sp. SWAT-3 was also observed in TARA_093, whereas *Photobacterium angustum* (cluster VI) was mainly found at coastal sites.

## Discussion

Methane, which exerts important roles in global warming and ozone destruction, has become a potent greenhouse gas. It has been reported that the concentration of CH_4_ in the upper oxygen-rich ocean waters is supersaturated. Several *Vibrio* species are able to grow rapidly in response to the addition of MPn and release CH_4_. In this study, MPn-demethylating *Vibrionales* strains were isolated, and it was found that most of *Vibrio* strains could efficiently demethylate MPn and produce methane. Genomic and transcriptomic results indicate that the *phn* operons, which showed high diversity among *Vibrio* species, were critical for the *Vibrio* strains to demethylate MPn. The potential contribution of *Vibrio* to methane production was estimated at the sampling sites and from the *Tara Ocean* database. These findings will help to better understand the microbial process of phosphonate demethylation and explain the ‘methane paradox’ in aerobic seawater.

### *Vibrio* spp. may be the ideal contributors in the conversion of MPn into methane

Microbial demethylation of MPn can release methane into seawater, contributing to the methane oversaturation in upper oceanic waters. As indicated by the incubation experiments described herein, emendation of MPn into the Pi-starved seawater stimulated rapid accumulation of *Vibrio* biomass and substantial methane release (Fig. 1, Figs. S1 and S2). This suggests that MPn was effectively demethylated by *Vibrio* spp. This high efficiency of MPn demethylation depended on their metabolic capabilities of nutrients (e.g., C, N and P). Indeed, most of the MPn-demethylating strains screened in this study were isolated from coastal seawater with high nutrient concentrations and a high ratio of N:P (> 16:1). In the seawater near ZQ, the concentration of inorganic nitrogen and phosphate reached 58.1 μmol L^−1^ and 1.02 μmol L^−1^ (Liu et al. 2007). *Vibrio* isolates from such eutrophic environments can be highly active in nutrient metabolism and effectively convert MPn into methane. For example, *V. gallaecicus* HW2-07 and HW2-08, which were isolated from coastal seawater near ZQ, could demethylate approximately 70–80% of amended MPn after a 5-day incubation. In contrast, the others could demethylate approximately 20–40% (Fig. 1). As for other MPn-demethylating bacteria, *Trichodesmium* IMS101 could demethylate only approximately 20% of added MPn into methane during 10-day incubation (Beversdorf et al. 2010), whereas *Pseudomonas* sp. demethylated nearly 80% of MPn in freshwater during 3-day incubation (Wang et al. 2017). In some lakes, *Proteobacteria* could release > 500 nmol L^−1^ methane in one day (Gunthel et al. 2021). Therefore, *Vibrio* spp. is definitely one of the most important bacterial groups for MPn consumption in the aquatic environment.

In the coastal seawater near ZQ, the methane concentration varied from 4 to 877 nmol L^−1^ (Zhang et al. 2007), whereas the methane concentration in the Pacific Ocean is less than 6 nmol L^−1^ (Karl et al. 2008; Reeburgh 2007). In the incubation experiments conducted here, the methane concentration of oceanic seawater increased from 3 nmol L^−1^ (NPO) to 385 nmol L^−1^ (NPO-MPn) in three days. For most of the *Vibrio* isolates in this study, nearly 140–270 nmol L^−1^ methane could be accumulated within the same incubation time, equivalent to demethylating 14% to 27% of total amended MPn (1 μmol L^−1^). For *V. gallaecicus* HW2-07 and HW2-08, the accumulated methane would reach 440–490 nmol L^−1^. It should be noted that methane released into aerobic seawater would be oxidized by biotic processes (Drake et al. 2015). It has been reported that the conversion efficiency of MPn in coastal seawater could increase to ~ 60% when the biologic activity of methane oxidation was depressed (Ye et al. 2020). Moreover, the strong methane oxidation during incubation could reduce the final methane production from 600 nmol L^−1^ to 300 nmol L^−1^. Thus, only half of the methane (70–245 nmol L^−1^) produced by the *Vibrio* isolates may be released and detectable if incubation was conducted under such oxic condition for 3 days. This amount still could contribute a proportion between 23% and 82% of the released methane (385 nmol L^−1^). Our study demonstrated that marine *Vibrio* spp. are powerful contributors to methane production in aerobic seawater.

Phosphonates including MPn can increase the abundance of both the related microbes and the *phn* operons in Pi starved marine environments (Sosa et al. 2019). Quantitative analysis showed that *phnL* abundance was nearly 5.2 × 10^6^ copies L^−1^ in the coastal seawater of ZQ; half of the *phnL* reads were assigned to the genus *Vibrio*. Considering that *phn* genes are single-copied in *Vibrio* genomes, the abundance of MPn-demethylating *Vibrio* species could be estimated at ~ 2.6 × 10^6^ cells L^−1^ in ZQ seawater. This estimation is within reasonable range given an average *Vibrio* 16S rRNA gene abundance of 10^4^ to 10^8^ copies L^−1^ in estuarine and coastal waters (Zhang et al. 2018). In contrast, the *phnL* abundance of *Vibrio* was beneath detection levels in NPO (0 h), which may be attributed to the low concentration of nutrients in oceanic seawater that was insufficient for these species to maintain a high population (Moore et al. 2013). After incubation with amended C, N and MPn, the abundance of *phnL* in NPO-MPn (72 h) increased to nearly 3.9 × 10^6^ copies L^−1^. If it is assumed that 50% of the *phnL* sequences belonged to *Vibrio* spp., there would be almost 2 × 10^6^ copies L^−1^ of MPn-demethylating *Vibrio* in NPO-MPn samples, accounting for ~ 28% of the total (normalized by *Vibrio* 16S rRNA gene sequences, 6.9 × 10^6^ copies L^−1^). Since the 16S rRNA gene is multiple-copied in *Vibrio* genomes (Wang et al. 2019), the actual percentage of MPn-demethylating *Vibrio* cells should be higher than 28%. This evaluation could mean that *Vibrio* cells with *phn* operons increased in abundance with the addition of MPn in Pi-limited seawater.

### Distinct clusters of the phn operons may help *Vibrio* cells participate in MPn-demethylation more effectively

Results presented here show that the *phn* operons of *Vibrio* species were highly diverse in gene organization and could be clustered into nine types. Eight of these *phn* operon types included at least nine genes *phnGHIJKLMNP* and the transporter-encoding genes *phnCDE*/*phnCDEE*/*phnDCEE*, except cluster IX (Fig. 3). *phnG* ~ *phnP* is directly related to the process where P is transferred from MPn to PRPP, which further participates in the synthesis of important biomolecules, e.g., nucleotides (Ulrich et al. 2018). *phnCDE* render transportation of MPn into cells, and *phnF* is related to expression regulation of the *phn* operon. These genes are essential for MPn demethylation, and were up-regulated in the MPn-incubation samples. Unexpectedly, the up-regulated genes also included *act*, which encodes acetyltransferase and occurred beside *phnP* in the *phn* operons of clusters I–IV. The occurrence of *act* in the *phn* operons of other bacteria indicates that it might be involved in phosphonate metabolism rather than a random gene arrangement. The *phn* operon has a diverse composition among bacteria and there are other possible components in the *phn* operon in addition to the common *phn* genes (Huang et al. 2005; Martinez et al. 2013). For example, *fosX* in the *phn* operon of *Mesorhizobium loti* encodes an enzyme that breaks the epoxide ring of Fosfomycin, a widely used antibiotic (Fillgrove et al. 2007). The diverse functional components in *phn* operons may help bacteria to demethylate different types of phosphonates in the environment (Ulrich et al. 2018). Although the function of *act* remains unknown, results presented here suggest that the frequently occurring genes flanking common *phn* genes may be necessary components of the *phn* operon and participate to a certain degree in the process of phosphonate demethylation. There was no clear relationship between *phn* operon types and a methane production ability, which indicates complex gene-product relationships with MPn as a substrate.

The evolution of the *phn* operons in *Vibrio* species was well reflected in the phylogenetic analyses of *phnJ* and *phnL*, which are two essential functional components of the operon, suggesting concurrence of gene divergency and organization. Clusters I–IV were phylogenetically closed. However, cluster I had become a large clade that evolved independently (Figs. 2, 3 and Fig. S6). In comparison with cluster IV, the *phn* operons of cluster II and III may have lost the *phnCDE* genes but reserved the gene sets of *phnCDEE* and *phnDCEE* during evolution. Cluster I and II were among the most abundant and ubiquitous *phn* operon types in the coastal and oceanic seawater as evidenced by their frequent occurrence in the microbial community of ZQ and *Tara Ocean* and the MPn-demethylating *Vibrio* isolates obtained in this study. Cluster V was mainly affiliated with the Rumoiensis clade, including *V. casei* DSM 22364 and *V. rumoiensis* FERM P-14531. Expansion of mobile genetic elements in the genomes of these species implies ongoing genomic variation (Tanaka et al. 2020), which may result in the occurrence of the second *phn* operon (cluster IX) in *V. casei* DSM 22364. Genes *phnGHIJK* of cluster IX are likely to encode the core protein complex Phn(GHIJ)_2_ K (Ulrich et al. 2018). Although the implication is still unclear, the *phn* operons of cluster IX are actively spreading among pathogenic and conditional pathogenic *Vibrio* species.

The smaller chromosomes (Chr. 2) of *Vibrio* spp. are usually considered as the recipient of horizontally transferred genes, which may help them occupy a wide range of specialized niches (Lin et al. 2018; Zhang et al. 2018). However, the *phn* operons of clusters V, VII and IX were in Chr. 1 and cluster IX operon was predicted as genomic islands surrounded by ISs. These results suggest that Chr. 1 was also undergoing genomic change, like Chr. 2. In addition, some plasmids in *M. loti* and *S. meliloti* carry the *phn* operons, and their *phn* genes were speculated to be transferred to a broad range of host groups via autonomous inheritance of plasmid genomes (Huang et al. 2005). No *Vibrio phn* operons was found to be carried by plasmids in this study. However, numerous studies have found a frequent occurrence of HGT in *Vibrio* spp. via transformation, conjugation and transduction (Le Roux and Blokesch 2018). In analyses presented here, the ISs around cluster IX operon of *V. vulnificus* Vv180806 and the phage sequences next to the *phn* operon of *V. rumoiensis* FERM P-14531 were all potential indicators of HGT events. The uncertainty of the *phn* operons present in *Vibrio* strains of the same species also underscored the importance of HGT. These phenomena indicate that the distribution of *phn* operons in *Vibrio* spp. is related to vertical genomic evolution and affected by HGT, although it may not be sufficient to characterize the HGT pathways.

### Does *Vibrio* spp. prefer Pi or MPn?

Although Pi starvation was considered as a premise for microbial demethylation of MPn and thereby methane production (Karl et al. 2008; Sosa et al. 2019), a recent study demonstrated that Pi did not strongly restrict this process in eutrophic coastal waters (Ye et al. 2020). The major MPn decomposers in coastal water, such as *Vibrio* spp., may not have an obvious preference for Pi or MPn. In fact, WXL531 was found to maintain a similar abundance during incubation with either Pi or MPn and could rapidly switch the cell status from Pi to MPn metabolism (Fig. 4 and Table S5). In contrast, WXL032 tended to utilize Pi in preference to MPn, suggesting that not all *Vibrio* species exhibit the same preference for Pi or MPn. This preference for different P sources explains why *Vibrio* spp. show different responses and adaptive capabilities to changes in P source, which may be a result of them chronically adapting to dynamic oceanic environments (White and Metcalf 2007). The preference for MPn or other phosphonates acts as a strategy for them to gain access to P nutrient in the Pi-limited environment.

## Conclusions

In this study, it was found that the MPn-demethylating *Vibrionales* strains could efficiently demethylate MPn. In comparison with oceanic seawater, the eutrophic coastal waters were enriched with abundant vibrios with the *phn* operons. The *Vibrio* spp. with the *phn* operons in oceanic seawater were able to rapidly respond to the amended MPn. We speculated that the MPn-demethylating *Vibrio* species may account for more than 28% of all thriving *Vibrio* species, contributing at least 20% of the methane production during a 3-day incubation period. The *phn* operons were critical for these species to demethylate MPn and were highly diverse. The identified diversity of the *phn* operon types and the ability for heterogeneity of MPn metabolism in *Vibrio* spp. would help to better understand the microbial process of phosphonate demethylation and explain the ‘methane paradox’ in aerobic seawater.

## Supplementary Information

Below is the link to the electronic supplementary material.

Supplementary file1 (DOCX 1742 kb)

## Data Availability

The genomic sequences in this study were submitted to NCBI database under accession numbers GCA_002214345.1 (WXL531), CP090614-CP090617 (WXL662) and CP090843-CP090855. The high-throughput sequencing data of this study were deposited in the Sequence Read Archive of NCBI, and are available under accession numbers, SRR17288730 (ZQ_phnJ), SRR17289282 (ZQ_phnL) and SRR17373641-SRR17373646 (transcriptomes). The *phnJ* and *phnL* sequences of cultured vibrios sequenced using the *Vibrio*-specific primers were submitted to NCBI database under accession numbers OL961487-OL961509.
