# Supplementary material for: Diverse marine Vibrio species convert methylphosphonate to methane
Source: Mar Life Sci Technol. 2025 Feb 20;7(3):492–506. doi: 10.1007/s42995-025-00278-w (PMC12413358; doi:10.1007/s42995-025-00278-w)
Supplement: Supplementary file 1 — Supplementary file1 (DOCX 1742 kb) [file 42995_2025_278_MOESM1_ESM.docx]

**Supplementary materials for:**

**Diverse marine *Vibrio* species convert methylphosphate to methane**

**Shu-Xian Yu^1,2†^, Xiaolei Wang^1,2,4†^, Yan Wang^1^, Haonan Wang^3^, Jiwen Liu^1,2,4^, Wen Hong^1^, Yunhui Zhang^1,2,4^, Min Yu^1,2,4^, Gui-Ling Zhang^3^, Fabiano Thompson^5^ and Xiao-Hua Zhang^1,2,4*^**

^1^Frontiers Science Center for Deep Ocean Multispheres and Earth System, and College of Marine Life Sciences, Ocean University of China, Qingdao, China

^2^Laboratory for Marine Ecology and Environmental Science, Qingdao Marine Science and Technology Center, Qingdao, China

^3^Key Laboratory of Marine Chemistry Theory and Technology, Ministry of Education/Institute for Advanced Ocean Study, Ocean University of China, Qingdao, China

^4^Institute of Evolution & Marine Biodiversity, Ocean University of China, Qingdao 266003, China

^5^Institute of Biology and Coppe, Federal University of Rio de Janeiro (UFRJ), Rio 21941-599, Brazil

*Author for correspondence:

Xiao-Hua Zhang, E-mail: xhzhang@ouc.edu.cn.

**^†^**These authors contributed equally to this work.

**Running title:** Vibrios convert methylphosphate to methane


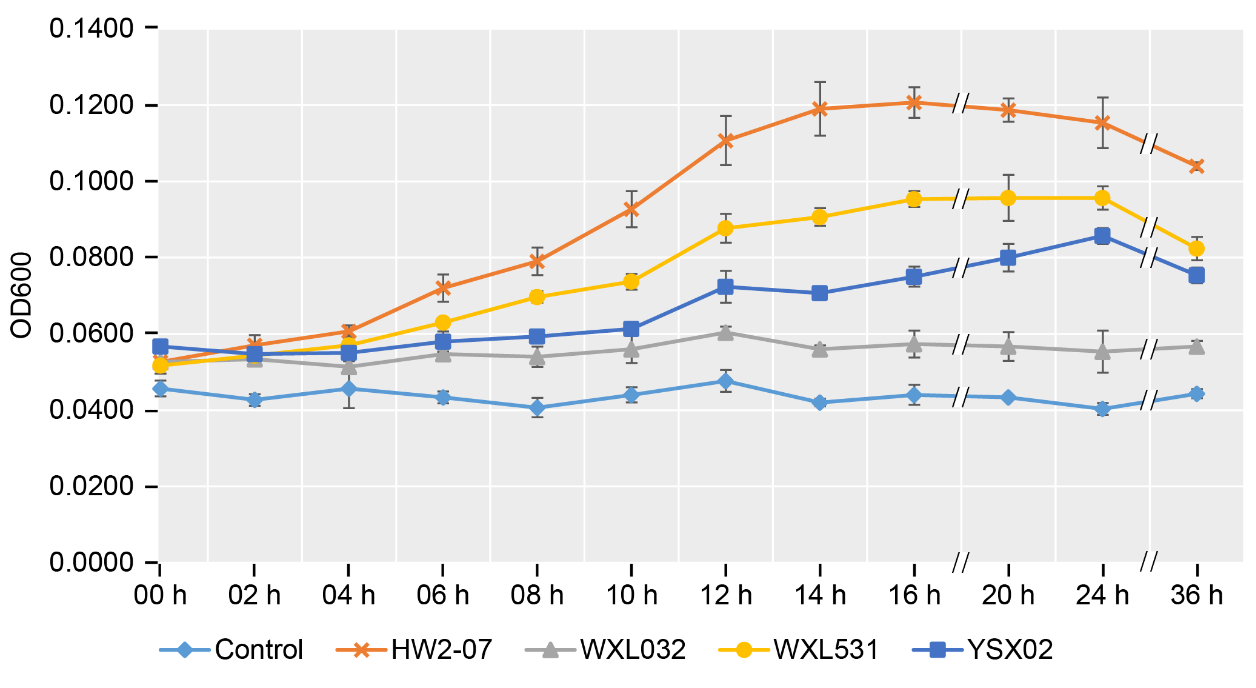


**Fig. S1 The mean optical absorbance at 600 nm (OD600) measured during incubation of *Vibrio* with MPn.** The error bars present 1 SD of triplicate samples. The control samples were amended with glucose, nitrate and MPn.


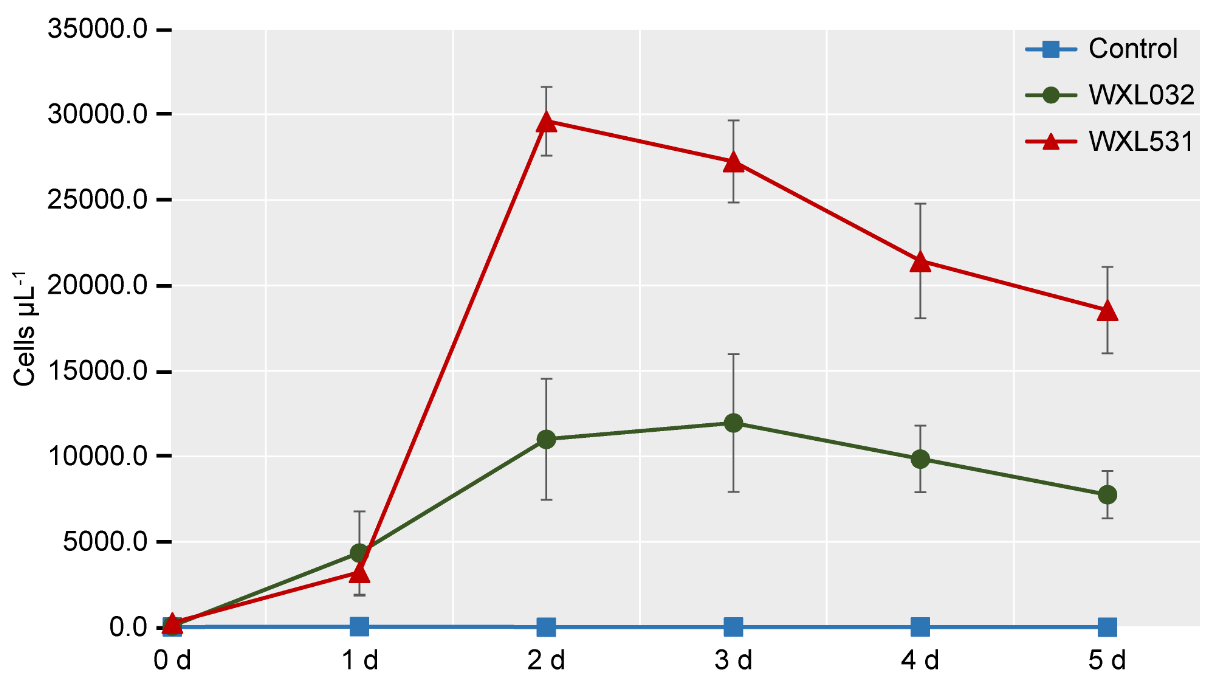


**Fig. S2 The mean concentrations of vibrios during the 5-day incubation in MPn medium measured by flow cytometry.** The error bars present 1 SD of triplicate samples. The control samples were amended with glucose, nitrate and MPn.


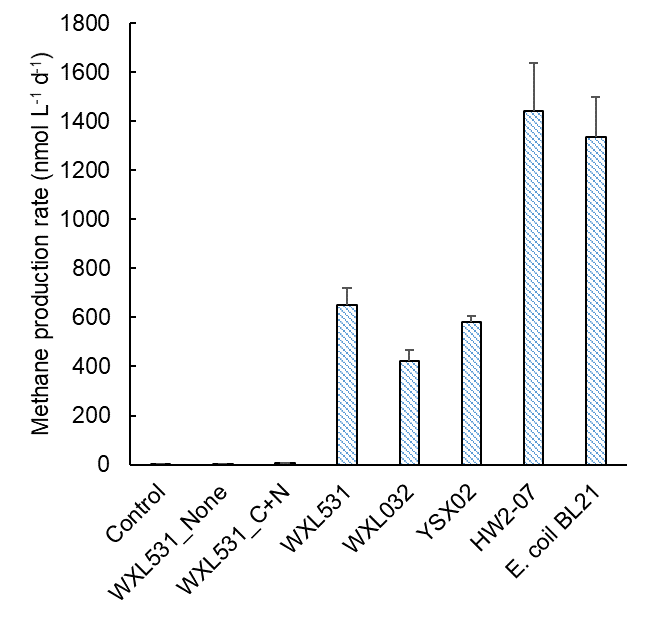


**Fig. S3 The daily methane production of 3 *Vibrio* strains and *E. coli* BL21 decomposing MPn (10 μmol L^-1^).** The error bars present 1 standard deviation (SD) of at least triplicate samples. The WXL531_None samples were incubated without glucose, nitrate and MPn. The WXL531_C+N samples were amended only with glucose and nitrate.


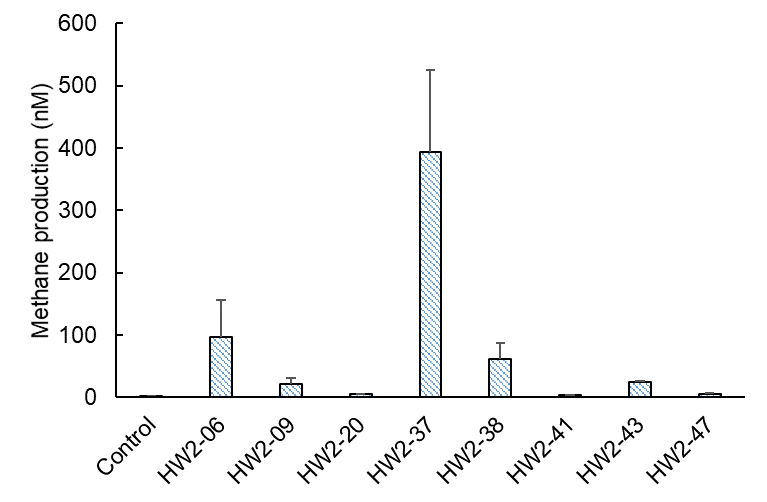


**Fig. S4 The accumulated methane production of eight none-vibrio isolates incubated in MPn medium for 5 days.** The error bars present 1 SD of triplicate samples.


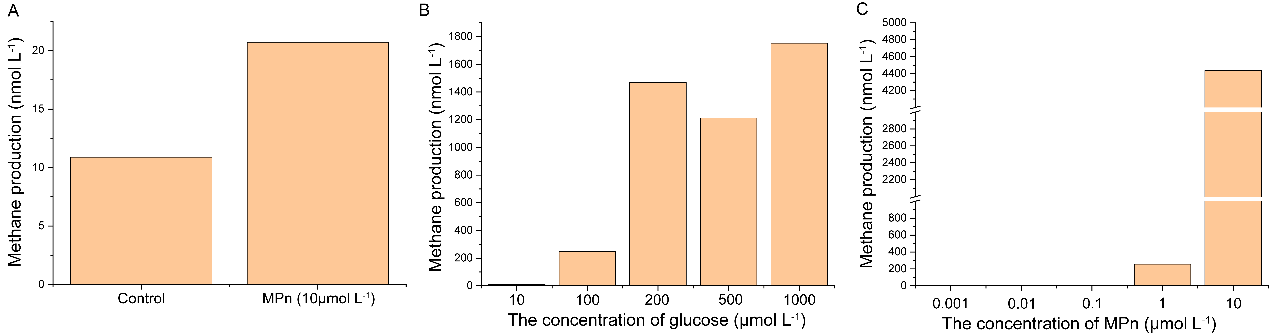


**Fig.** **S5 The methane production of *Vibrio gallaecicus* HW2-07 under a series of conditions with incubation for 3 days.** A, MPn as the sole carbon and phosphorus sources; B, under a series of glucose concentrations; C, under a series of MPn concentrations.


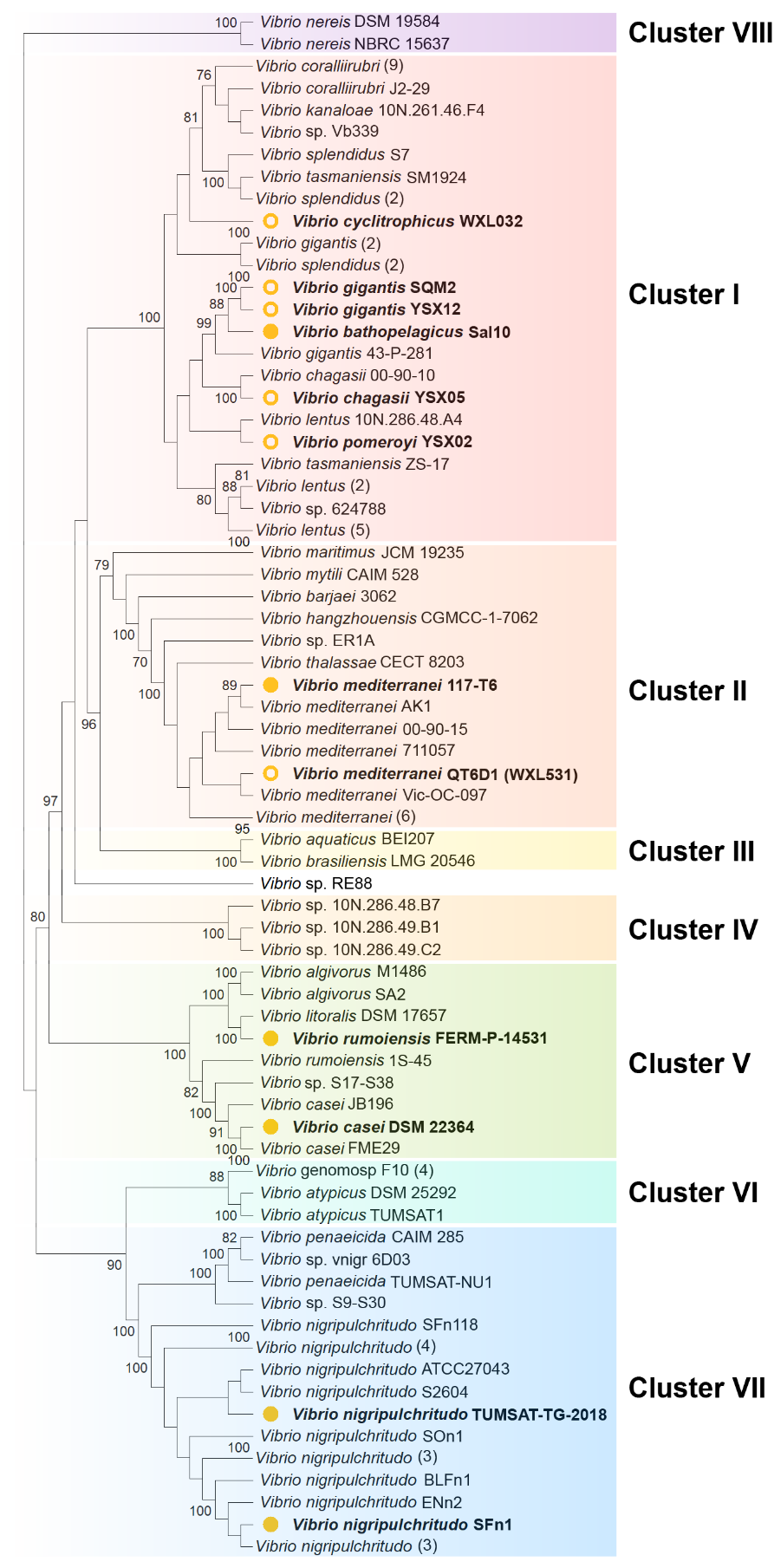


**Fig. S6 Phylogenetic analysis of 101 *phnL* sequences from *Vibrio* genomes. The bootstrap values (≥ 70%) were shown in the phylogenetic tree.** The *phnL* sequences from NCBI complete genomes were marked with dots, whereas those sequences from our laboratory were labelled with circles. Genomes that contain at least *phnG-phnP* were colored by orange. The branches of the same species were folded as much as possible, and their quantity was displayed in parentheses.


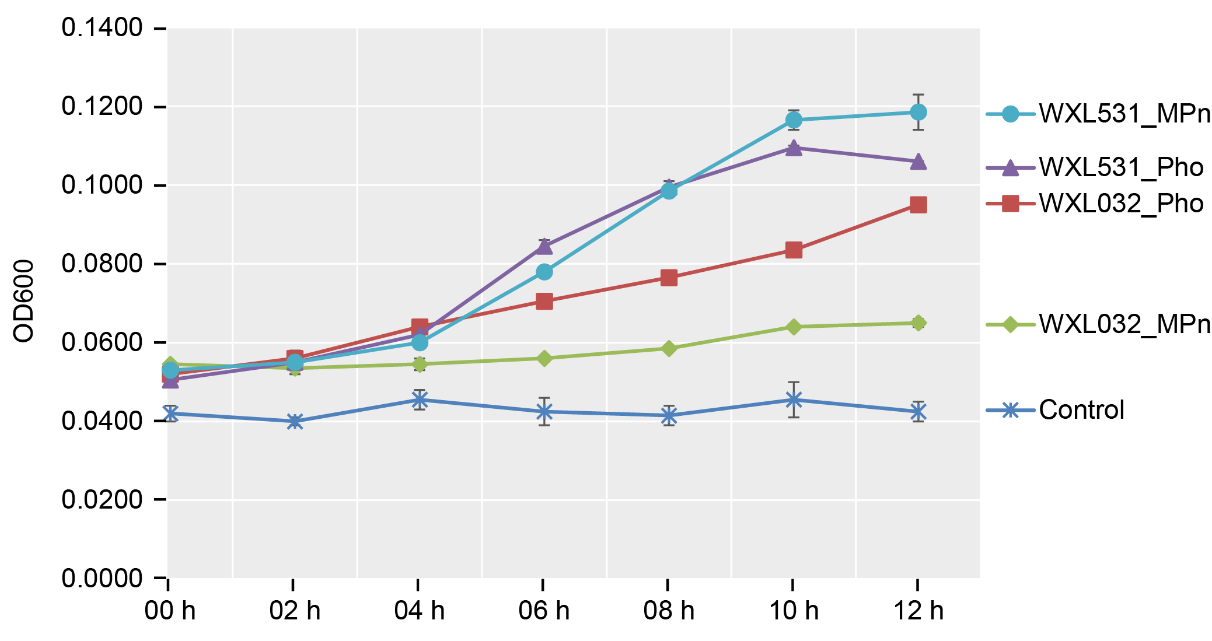


**Fig. S7 The mean optical absorbance at 600 nm (OD600) measured during the vibrio incubation with MPn and phosphate (Pho).** The error bars present 1 SD of triplicate samples. The control samples were amended with glucose and nitrate.


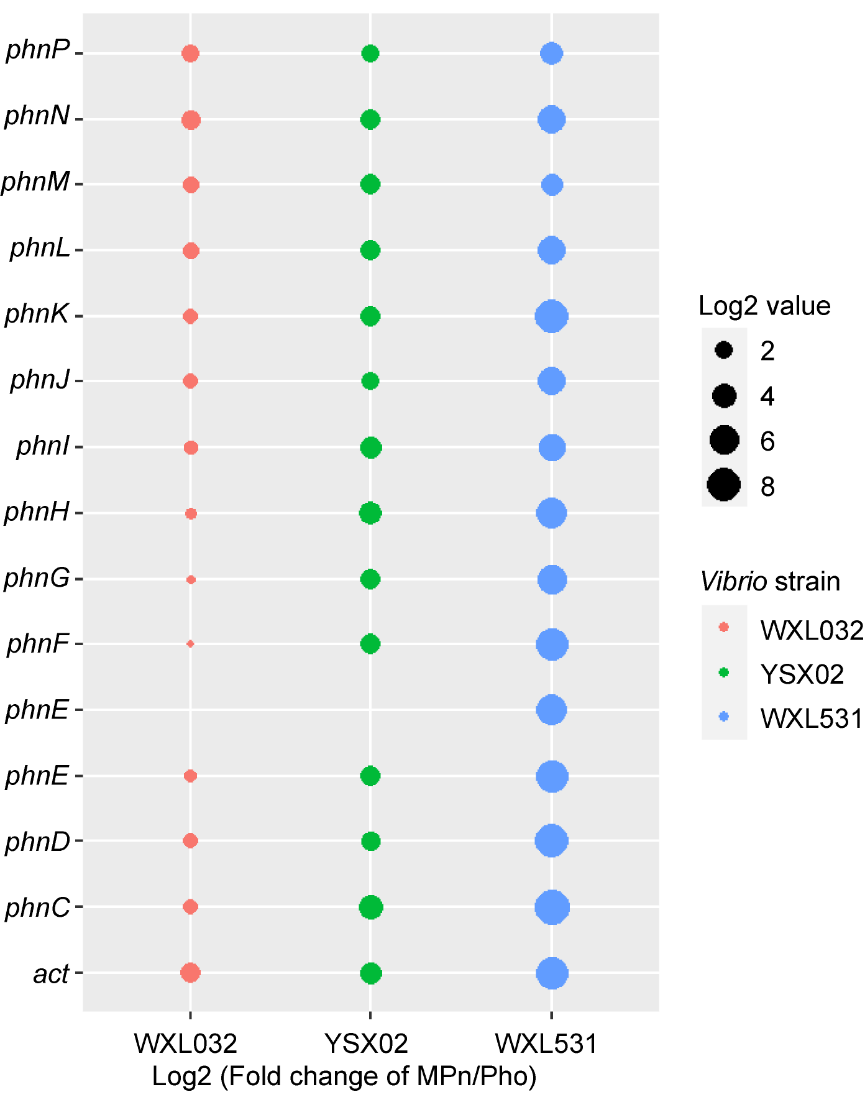


**Fig. S8 The log2 values of fold change shown for *phn* gene expression in MPn incubation samples when compared with the phosphate cultures.** act, acetyltransferase.


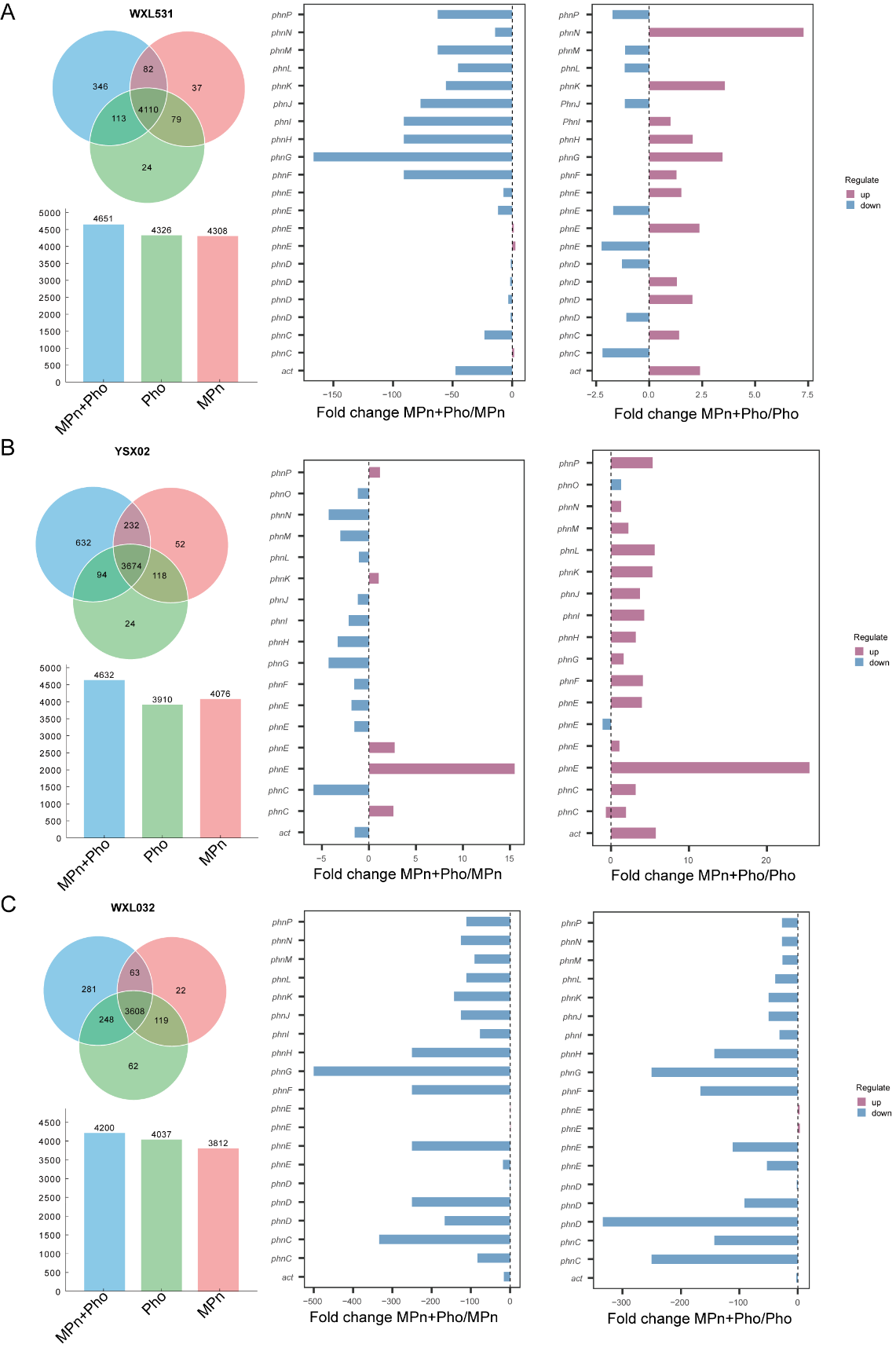


**Fig. S9 The expressed gene number and the fold change of phn gene expression in the *Vibrio* strains when they were incubated with MPn + Pho, MPn and phosphate (A~B), respectively.**


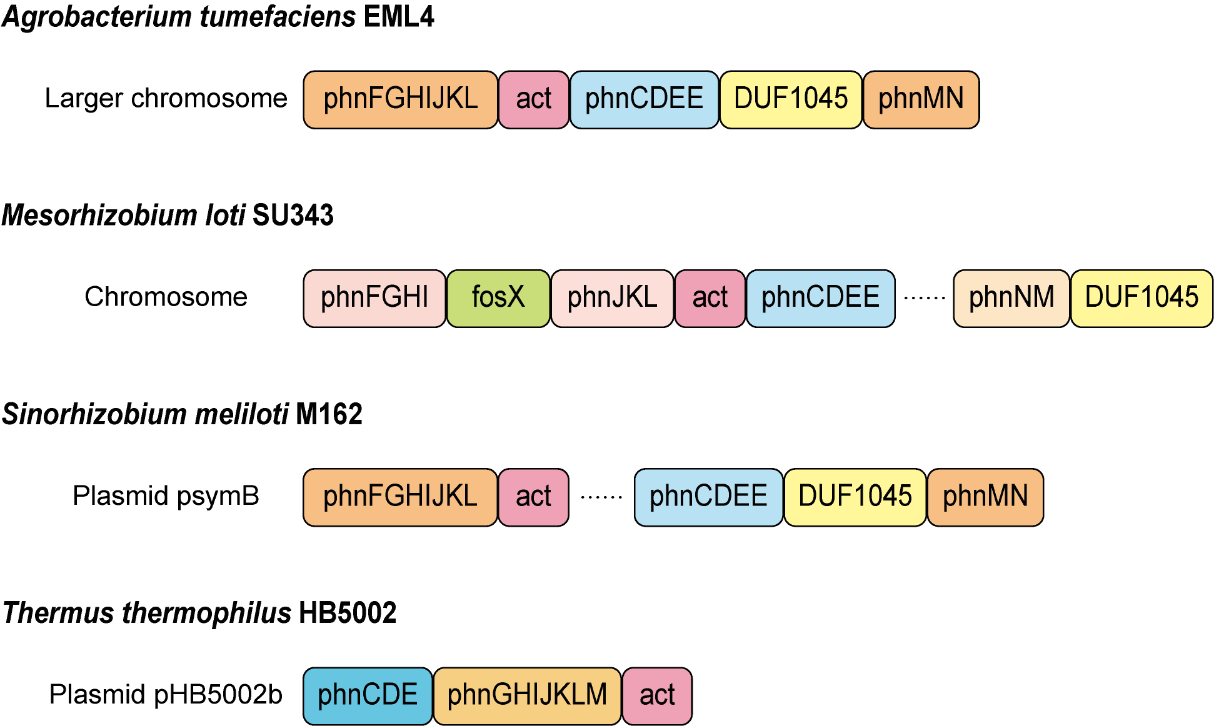


**Fig. S10 The *phn* operon examples of four bacterial strains. fosX, fosfomycin resistant protein subfamily FosX; act, acetyltransferase.** The *A. tumefaciens* EML4 have two chromosomes, and the *phn* operon was on the larger one.

**Table S1. Effective *Vibrio*-specific primers used in this study.**

| **Gene** | **Primer** | **Sequence** | **Annealing Temperature (**℃**)** |
| --- | --- | --- | --- |
| *phnJ* | 94F | GGYTACAACTAYGGYTACCT | 54 |
|  | 620F | TACATCATGGAYCCDTCKCC | 54 |
|  | 895R | GCARTAATCVGTRTCKGAACA | 54 |
| *phnL* | 63F | AASCTTTGTKCTSCAYAAYCAA | 53 |
|  | 370F | GARGTSGTGATGCARCCTWTG | 54 |
|  | 672R | TTCATCRTGGAARATDCCSA | 53-54 |

**Table S2. *Vibrio* strains tested in this study.**

| **Isolate_ID** | **Top-hit strain** | **Similarity (%)** | **Genome sequencing** | **Publish/Accession** | **MPn→CH_4_** | ***phnJ*** | | ***phnL*** | |
| --- | --- | --- | --- | --- | --- | --- | --- | --- | --- |
|  |  |  |  |  |  | **94F_895R** | **620F_896R** | **63F_672R** | **370F_673R** |
| WXL032 | *Vibrio cyclitrophicus* ECSMB14105 | ANI, 99.09; DDH, 91.40 | complete | CP090847-CP090848 | + | + | + | + | + |
| WXL531 | *Vibrio mediterranei* QT6D1 | - | complete | GCA_002214345.1 | + | + | + | + | + |
| SQM2 | *Vibrio gigantis* CAIM 25 | 16S rRNA gene, 99.93 | complete | unpublished | + | + | + | + | + |
| A6-11SW3 | *Vibrio kanaloae* R17 | ANI 98.45, DDH 85.20 | complete | CP090843-CP090844 | - | - | - | - | - |
| WXL161 | *Vibrio kanaloae* R17 | ANI 98.40, DDH 85.20 | complete | CP090845-CP090846 | - | - | - | - | - |
| WXL662 | *Vibrio pelagius* CECT 4202 | 16S rRNA gene, 99.52 | complete | CP090614-CP090617 | - | - | - | - | - |
| YSX02 | *Vibrio pomeroyi* LMG 20537 | 16S rRNA gene, 100 | complete | CP090854-CP090855 | + | + | + | + | + |
| YSX05 | *Vibrio chagasii* ECSMB14107 | ANI, 96.34; DDH, 68.60 | complete | CP090851-CP090853 | + | + | + | + | + |
| YSX12 | *Vibrio gigantis* CAIM 25 | 16S rRNA gene, 100 | complete | CP090849-CP090850 | + | + | + | + | + |
| HW1-01 | *Vibrio maerlii* G62 | 16S rRNA gene, 98.22 | no | this study | + | + | + | - | + |
| HW1-02 | *Vibrio algivorus* SA2 | 16S rRNA gene, 99.87 | no | this study | + | + | + | + | + |
| HW1-03 | *Vibrio pomeroyi* LMG 20537 | 16S rRNA gene, 100 | no | this study | + | + | + | + | + |
| HW1-11 | *Vibrio hangzhouensis* CN83 | 16S rRNA gene, 100 | no | this study | + | + | + | + | + |
| HW1-17 | *Vibrio maerlii* G62 | 16S rRNA gene, 98.23 | no | this study | + | + | + | - | + |
| HW1-28 | *Photobacterium lutimaris* DF-42 | 16S rRNA gene, 98.54 | no | this study | + | - | + | - | + |
| HW1-29 | *Vibrio hangzhouensis* CN83 | 16S rRNA gene, 100 | no | this study | + | + | + | + | + |
| HW2-07 | *Vibrio gallaecicus* VB 8.9 | 16S rRNA gene, 97.41 | no | this study | + | - | + | + | + |
| HW2-08 | *Vibrio gallaecicus* VB 8.9 | 16S rRNA gene, 97.55 | no | this study | + | - | + | + | + |
| HW2-13 | *Vibrio alginolyticus* NBRC 15630 | 16S rRNA gene, 98.93 | no | this study | - | - | - | - | - |
| HW2-14 | *Vibrio pomeroyi* LMG 20537 | 16S rRNA gene, 100 | no | this study | - | - | - | - | - |
| HW2-29 | *Vibrio tasmaniensis* LMG 21574 | 16S rRNA gene, 99.47 | no | this study | - | - | - | - | - |
| HW2-31 | *Vibrio pomeroyi* LMG 20537 | 16S rRNA gene, 100 | no | this study | - | - | - | - | - |
| HW2-32 | *Vibrio penaeicida* DSM 14398 | 16S rRNA gene, 99.73 | no | this study | + | - | + | - | - |
| HW2-33 | *Vibrio nigripulchritudo* ATCC 27043 | 16S rRNA gene, 100 | no | this study | + | + | + | + | + |
| HW2-34 | *Vibrio penaeicida* DSM 14398 | 16S rRNA gene, 99.73 | no | this study | - | - | - | - | - |
| HW2-42 | *Vibrio nigripulchritudo* ATCC 27043 | 16S rRNA gene, 99.87 | no | this study | + | + | + | + | + |

ANI, Average Nucleotide Identity; DDH, DNA-DNA hybridization; +/-, positive/negative result. Identification of vibrios were performed preferentially.

**Table S3. Strains tested in this study.**

| **Isolate_ID** | **Taxon-order** | **Top-hit strain** | **Similarity (%)** | **Genome sequencing** | **Publish/**  **Accession** | **MPn→CH_4_** | ***phnJ*** | ***phnL*** |
| --- | --- | --- | --- | --- | --- | --- | --- | --- |
|  |  |  |  |  |  |  | **620F_896R** | **370F_673R** |
| HW2-06 | *Rhodobacterales* | *Sulfitobacter pontiacus* DSM 10014 | 16S rRNA gene, 100 | no | this study | + | + | - |
| HW2-09 | *Rhodobacterales* | *Yoonia* sp. PT4BL | 16S rRNA gene, 100 | no | this study | - | - | - |
| HW2-20 | *Oceanospirillales* | *Marinobacterium* sp. EthaneSIP4-6-15 | 16S rRNA gene, 100 | no | this study | - | + | - |
| HW2-37 | *Rhodobacterales* | *Pacificibacter aestuarii* KJ21 | 16S rRNA gene, 98.27 | no | this study | + | + | - |
| HW2-38 | *Rhodobacterales* | *Sulfitobacter pontiacus* DSM 10014 | 16S rRNA gene, 100 | no | this study | + | + | - |
| HW2-41 | *Rhodobacterales* | *Lentibacter algarum* DSM 24677 | 16S rRNA gene, 100 | no | this study | - | - | - |
| HW2-43 | *Rhodobacterales* | *Sulfitobacter pontiacus* DSM 10014 | 16S rRNA gene, 100 | no | this study | - | - | - |
| HW2-47 | *Sphingomonadales* | *Qipengyuania flava* SW-46 | 16S rRNA gene, 100 | no | this study | - | - | - |

+/-, positive/negative result.

**Table S4. Available genome information from NCBI database and their taxonomic information from LPSN.**

| **#Organism Name** | **Size (Mb)** | **No. of Chr.** | **No. of Plasmids** | **Summary** | | **Assemblies at complete level** | | **Assemblies at chromosome, contig and scaffold level** | | ***phn* cluster type** | ***Vibrio* clade** | **Species in LPSN** | **References** |
| --- | --- | --- | --- | --- | --- | --- | --- | --- | --- | --- | --- | --- | --- |
|  |  |  |  | ***phn* clusters** | **Assemblies** | ***phn* clusters** | **Assemblies** | ***phn* clusters** | **Assemblies** |  |  |  |  |
| *Vibrio* | 6.59 | 2 | 0 | 15 | 249 | 0 | 13 | 15 | 236 | I(3), II(1), IV(3), V(1), VI(4), VII(2) and *phnCDEFGHIJKLMNP_act* | F10, Splendidus | *Vibrio* | - |
| *Vibrio aerogenes* | 5.25 | 0 | 0 | 0 | 1 | 0 | 0 | 0 | 1 | None | Gazogenes | *Vibrio aerogenes* | Shieh et al. 2000 |
| *Vibrio aestuarianus* | 4.15 | 0 | 0 | 0 | 12 | 0 | 0 | 0 | 12 | None | Anguillarum | *Vibrio aestuarianus* | Tison and Seidler 1983 |
| *Vibrio agarilyticus* | 4.35 | 0 | 0 | 0 | 1 | 0 | 0 | 0 | 1 | None |  | *Vibrio agarilyticus* | Wang et al. 2021 |
| *Vibrio agarivorans* | 4.50 | 0 | 0 | 0 | 1 | 0 | 0 | 0 | 1 | None |  | *Vibrio agarivorans* | Macián et al. 2001 |
| *Vibrio alfacsensis* | 4.91 | 2 | 1 | 0 | 1 | 0 | 0 | 0 | 1 | None |  | *Vibrio alfacsensis* | Gomez-Gil et al. 2012 |
| *Vibrio alginolyticus* | 5.18 | 0 | 0 | 0 | 146 | 0 | 27 | 0 | 119 | None | Harveyi | *Vibrio alginolyticus* | Miyamoto et al. 1961; Sakazaki 1968 (Approved Lists 1980) |
| *Vibrio algivorus* | 3.93 | 0 | 0 | 2 | 2 | 0 | 0 | 2 | 2 | V(2) |  | *Vibrio algivorus* | Doi et al. 2016 |
| *Vibrio anguillarum* | 3.85 | 2 | 0 | 5 | 130 | 2 | 14 | 3 | 116 | IX(5) | Anguillarum | *Vibrio anguillarum* | Bergman 1909 (Approved Lists 1980) |
| *Vibrio antiquarius* | 5.09 | 2 | 0 | 0 | 3 | 0 | 1 | 0 | 2 | None |  | *"Vibrio antiquarius"* | Hasan et al. 2015 |
| *Vibrio aphrogenes* | 3.38 | 2 | 0 | 0 | 1 | 0 | 1 | 0 | 0 | None |  | *Vibrio aphrogenes* | Tanaka et al. 2018 |
| *Vibrio aquaticus* | 4.45 | 0 | 0 | 1 | 1 | 0 | 0 | 1 | 1 | III(1) |  | *Vibrio aquaticus* | Li et al. 2020 |
| *Vibrio aquimaris* | 4.50 | 1 | 3 | 0 | 1 | 0 | 1 | 0 | 0 | None |  | *"Vibrio aquimaris"* | Franco et al. 2020 |
| *Vibrio astriarenae* | 4.80 | 2 | 0 | 0 | 1 | 0 | 1 | 0 | 0 | None |  | *Vibrio astriarenae* | Al-Saari et al. 2016 |
| *Vibrio atlanticus* | 4.97 | 2 | 0 | 0 | 4 | 0 | 1 | 0 | 3 | None | Splendidus | *Vibrio atlanticus* | Diéguez et al. 2011 |
| *Vibrio atypicus* | 4.84 | 0 | 0 | 2 | 2 | 0 | 0 | 2 | 2 | VI(2) |  | *Vibrio atypicus* | Wang et al. 2010 |
| *Vibrio azureus* | 4.83 | 2 | 2 | 0 | 3 | 0 | 0 | 0 | 3 | None | Harveyi | *Vibrio azureus* | Yoshizawa et al. 2009 |
| *Vibrio barjaei* | 5.54 | 0 | 0 | 1 | 1 | 0 | 0 | 1 | 1 | II(1) |  | *Vibrio barjaei* | Dubert et al. 2017 |
| *Vibrio bathopelagicus* | 5.67 | 2 | 0 | 1 | 1 | 1 | 1 | 0 | 0 | I(1) | Splendidus | None | - |
| *Vibrio bivalvicida* | 4.92 | 0 | 0 | 0 | 1 | 0 | 0 | 0 | 1 | None |  | *Vibrio bivalvicida* | Dubert et al. 2016 |
| *Vibrio brasiliensis* | 4.72 | 0 | 0 | 1 | 1 | 0 | 0 | 1 | 1 | III(1) | Orientalis | *Vibrio brasiliensis* | Thompson et al. 2003 |
| *Vibrio breoganii* | 4.05 | 0 | 0 | 0 | 59 | 0 | 1 | 0 | 58 | None | Halioticoli | *Vibrio breoganii* | Beaz Hidalgo et al. 2009 |
| *Vibrio campbellii* | 5.22 | 2 | 1 | 0 | 75 | 0 | 10 | 0 | 65 | None | Harveyi | *Vibrio campbellii* | Baumann et al. 1971; Baumann et al. 1981 |
| *Vibrio caribbeanicus* | 4.41 | 0 | 0 | 0 | 2 | 0 | 0 | 0 | 2 | None | Pectenicida | *Vibrio caribbeanicus* | Hoffmann et al. 2012 |
| *Vibrio casei* | 3.99 | 0 | 0 | 8 | 4 | 2 | 1 | 6 | 3 | V(4) + IX(4) | Rumoiensis | *Vibrio casei* | Bleicher et al. 2010 |
| *Vibrio celticus* | 5.32 | 0 | 0 | 0 | 2 | 0 | 0 | 0 | 2 | None |  | *Vibrio celticus* | Beaz-Hidalgo et al. 2011 |
| *Vibrio chagasii* | 5.30 | 0 | 0 | 1 | 6 | 0 | 1 | 1 | 5 | I(1) | Splendidus | *Vibrio chagasii* | Thompson et al. 2003 |
| *Vibrio chemaguriensis* | 5.01 | 0 | 0 | 0 | 1 | 0 | 0 | 0 | 1 | None |  | *"Vibrio chemaguriensis"* | Ghosh and Bhadury 2019 |
| *Vibrio cholerae* | 4.03 | 2 | 0 | 1 | 1552 | 1 | 92 | 0 | 1460 | IX(1) | Cholerae | *Vibrio cholerae* | Pacini 1854 (Approved Lists 1980) |
| *Vibrio cidicii* | 4.67 | 0 | 0 | 0 | 7 | 0 | 1 | 0 | 6 | None |  | *Vibrio cidicii* | Orata et al. 2016 |
| *Vibrio cincinnatiensis* | 3.80 | 0 | 0 | 0 | 6 | 0 | 4 | 0 | 2 | None | Cholerae | *Vibrio cincinnatiensis* | Brayton et al. 1986 |
| *Vibrio comitans* | 4.43 | 0 | 0 | 0 | 1 | 0 | 0 | 0 | 1 | None |  | *Vibrio comitans* | Sawabe et al. 2007 |
| *Vibrio coralliilyticus* | 5.55 | 0 | 0 | 0 | 32 | 0 | 6 | 0 | 26 | None | Coralliilyticus | *Vibrio coralliilyticus* | Ben-Haim et al. 2003 |
| *Vibrio coralliirubri* | 5.38 | 0 | 0 | 9 | 19 | 0 | 0 | 9 | 19 | I(9) |  | *Vibrio coralliirubri* | Poli et al. 2018 |
| *Vibrio crassostreae* | 5.15 | 0 | 0 | 1 | 168 | 0 | 0 | 1 | 168 | I(1) | Splendidus | *Vibrio crassostreae* | Faury et al. 2004 |
| *Vibrio cyclitrophicus* | 5.07 | 2 | 0 | 0 | 85 | 0 | 1 | 0 | 84 | None | Splendidus | *Vibrio cyclitrophicus* | Hedlund and Staley 2001 |
| *Vibrio diabolicus* | 5.43 | 2 | 0 | 0 | 22 | 0 | 6 | 0 | 16 | None |  | *Vibrio diabolicus* | Raguénès et al. 1997 |
| *Vibrio diazotrophicus* | 4.61 | 0 | 0 | 0 | 9 | 0 | 0 | 0 | 9 | None | Diazotrophicus | *Vibrio diazotrophicus* | Guerinot et al. 1982 |
| *Vibrio echinoideorum* | 5.63 | 0 | 0 | 0 | 1 | 0 | 0 | 0 | 1 | None |  | *Vibrio echinoideorum* | Hira et al. 2019 |
| *Vibrio europaeus* | 5.47 | 0 | 1 | 0 | 4 | 0 | 2 | 0 | 2 | None |  | *Vibrio europaeus* | Prado et al. 2016; Dubert et al. 2016 |
| *Vibrio ezurae* | 3.71 | 0 | 0 | 0 | 2 | 0 | 0 | 0 | 2 | None | Halioticoli | *Vibrio ezurae* | Sawabe et al. 2005 |
| *Vibrio fluvialis* | 4.83 | 2 | 0 | 2 | 58 | 0 | 9 | 2 | 49 | IX(2) | Cholerae | *Vibrio fluvialis* | Lee et al. 1981 |
| *Vibrio fortis* | 5.29 | 0 | 0 | 0 | 3 | 0 | 0 | 0 | 3 | None | Splendidus | *Vibrio fortis* | Thompson et al. 2003 |
| *Vibrio fujianensis* | 3.62 | 0 | 0 | 0 | 1 | 0 | 0 | 0 | 1 | None |  | *Vibrio fujianensis* | Fang et al. 2018 |
| *Vibrio furnissii* | 4.99 | 2 | 1 | 0 | 15 | 0 | 6 | 0 | 9 | None | Cholerae | *Vibrio furnissii* | Brenner et al. 1984 |
| *Vibrio galatheae* | 4.07 | 0 | 0 | 0 | 1 | 0 | 0 | 0 | 1 | None |  | *Vibrio galatheae* | Giubergia et al. 2016 |
| *Vibrio gallaecicus* | 5.00 | 0 | 0 | 0 | 2 | 0 | 0 | 0 | 2 | None |  | *Vibrio gallaecicus* | Beaz-Hidalgo et al. 2009 |
| *Vibrio gangliei* | 3.61 | 0 | 0 | 0 | 1 | 0 | 0 | 0 | 1 | None |  | *Vibrio gangliei* | Meng et al. 2018 |
| *Vibrio gazogenes* | 4.64 | 0 | 0 | 0 | 4 | 0 | 1 | 0 | 3 | None | Gazogenes | *Vibrio gazogenes* | Harwood et al. 1980; Baumann et al. 1981 |
| *Vibrio gigantis* | 5.68 | 0 | 0 | 3 | 3 | 0 | 0 | 3 | 3 | I(3) | Splendidus | *Vibrio gigantis* | Le Roux et al. 2005 |
| *Vibrio halioticoli* | 4.09 | 0 | 0 | 0 | 2 | 0 | 0 | 0 | 2 | None | Halioticoli | *Vibrio halioticoli* | Sawabe et al. 1998 |
| *Vibrio hangzhouensis* | 5.12 | 0 | 0 | 1 | 1 | 0 | 0 | 1 | 1 | II(1) |  | *Vibrio hangzhouensis* | Xu et al. 2009 |
| *Vibrio harveyi* | 5.88 | 2 | 0 | 2 | 50 | 0 | 6 | 2 | 44 | IX(2) | Harveyi | *Vibrio harveyi* | Johnson and Shunk 1936; Baumann et al. 1981 |
| *Vibrio hepatarius* | 4.60 | 0 | 0 | 0 | 4 | 0 | 0 | 0 | 4 | None | Orientalis | *Vibrio hepatarius* | Thompson et al. 2003 |
| *Vibrio hibernica* | 3.43 | 0 | 0 | 1 | 1 | 0 | 0 | 1 | 1 | IX(1) |  | *"Vibrio hibernica"* | Woods et al. 2020 |
| *Vibrio hyugaensis* | 5.62 | 0 | 0 | 0 | 6 | 0 | 1 | 0 | 5 | None |  | *Vibrio hyugaensis* | Urbanczyk et al. 2015 |
| *Vibrio ichthyoenteri* | 4.64 | 0 | 0 | 0 | 1 | 0 | 0 | 0 | 1 | None | Scopthalmi | *Vibrio ichthyoenteri* | Ishimaru et al. 1996 |
| *Vibrio injenensis* | 3.67 | 0 | 0 | 0 | 1 | 0 | 0 | 0 | 1 | None |  | *"Vibrio injenensis"* | Paek et al. 2017 |
| *Vibrio inusitatus* | 4.35 | 0 | 0 | 0 | 1 | 0 | 0 | 0 | 1 | None | Halioticoli | *Vibrio inusitatus* | Sawabe et al. 2007 |
| *Vibrio ishigakensis* | 4.89 | 0 | 0 | 0 | 3 | 0 | 0 | 0 | 3 | None |  | *Vibrio ishigakensis* | Gao et al. 2018 |
| *Vibrio jasicida* | 5.99 | 2 | 0 | 0 | 19 | 0 | 1 | 0 | 18 | None |  | *Vibrio jasicida* | Yoshizawa et al. 2012 |
| *Vibrio kanaloae* | 4.67 | 2 | 0 | 1 | 15 | 0 | 1 | 1 | 14 | I(1) | Splendidus | *Vibrio kanaloae* | Thompson et al. 2003 |
| *Vibrio lentus* | 5.56 | 0 | 0 | 8 | 88 | 0 | 0 | 8 | 88 | I(8) | Splendidus | *Vibrio lentus* | Macián et al. 2001 |
| *Vibrio litoralis* | 3.82 | 0 | 0 | 2 | 2 | 0 | 0 | 2 | 2 | V(1), IX(1) | Rumoiensis | *Vibrio litoralis* | Nam et al. 2007 |
| *Vibrio maerlii* | 4.76 | 0 | 0 | 0 | 1 | 0 | 0 | 0 | 1 | None |  | *"Vibrio maerlii"* | Silva et al. 2018 |
| *Vibrio mangrovi* | 4.94 | 0 | 0 | 0 | 1 | 0 | 0 | 0 | 1 | None |  | *Vibrio mangrovi* | Rameshkumar et al. 2011 |
| *Vibrio marinisediminis* | 4.59 | 0 | 0 | 0 | 1 | 0 | 0 | 0 | 1 | None |  | *"Vibrio marinisediminis"* | Zhao et al. 2021 |
| *Vibrio maritimus* | 5.85 | 0 | 0 | 1 | 1 | 0 | 0 | 1 | 1 | II(1) | Mediterranei | *Vibrio maritimus* | Chimetto et al. 2011 |
| *Vibrio mediterranei* | 5.65 | 0 | 0 | 11 | 11 | 1 | 1 | 10 | 10 | II(12) | Mediterranei | *Vibrio mediterranei* | Pujalte and Garay 1986 |
| *Vibrio metoecus* | 3.99 | 2 | 0 | 0 | 28 | 0 | 4 | 0 | 24 | None |  | *Vibrio metoecus* | Kirchberger et al. 2014 |
| *Vibrio metschnikovii* | 3.62 | 2 | 0 | 0 | 12 | 0 | 3 | 0 | 9 | None | Cholerae | *Vibrio metschnikovii* | Gamaleia 1888 (Approved Lists 1980) |
| *Vibrio mexicanus* | 4.81 | 0 | 0 | 0 | 1 | 0 | 0 | 0 | 1 | None |  | *Vibrio mexicanus* | González-Castillo et al. 2015 |
| *Vibrio mimicus* | 4.31 | 0 | 0 | 1 | 27 | 0 | 7 | 1 | 20 | IX(1) | Cholerae | *Vibrio mimicus* | Davis et al. 1982 |
| *Vibrio mytili* | 4.61 | 0 | 0 | 1 | 1 | 0 | 0 | 1 | 1 | II(1) | Harveyi | *Vibrio mytili* | Pujalte et al. 1993 |
| *Vibrio natriegens* | 5.18 | 2 | 0 | 0 | 9 | 0 | 5 | 0 | 4 | None | Harveyi | *Vibrio natriegens* | Payne et al. 1961; Baumann et al. 1981 |
| *Vibrio navarrensis* | 4.29 | 2 | 1 | 0 | 29 | 0 | 4 | 0 | 25 | None | Vulnificus | *Vibrio navarrensis* | Urdaci et al. 1991 |
| *Vibrio neocaledonicus* | 5.25 | 2 | 0 | 0 | 1 | 0 | 1 | 0 | 0 | None |  | *"Vibrio neocaledonicus"* | Chalkiadakis et al. 2013 |
| *Vibrio neptunius* | 5.20 | 0 | 0 | 0 | 6 | 0 | 0 | 0 | 6 | None | Coralliilyticus | *Vibrio neptunius* | Thompson et al. 2003 |
| *Vibrio nereis* | 4.09 | 0 | 0 | 2 | 2 | 0 | 0 | 2 | 2 | VIII(2) | Nereis | *Vibrio nereis* | Harwood et al. 1980; Baumann et al. 1981 |
| *Vibrio nigripulchritudo* | 6.06 | 0 | 0 | 18 | 18 | 2 | 2 | 16 | 16 | VII(18) | Nigripulchritudo | *Vibrio nigripulchritudo* | Baumann et al. 1971; Baumann et al. 1981 |
| *Vibrio nitrifigilis* | 5.48 | 0 | 0 | 0 | 1 | 0 | 0 | 0 | 1 | None |  | None | - |
| *Vibrio ordalii* | 3.78 | 0 | 0 | 0 | 2 | 0 | 0 | 0 | 2 | None | Anguillarum | *Vibrio ordalii* | Schiewe et al. 1982 |
| *Vibrio orientalis* | 4.70 | 0 | 0 | 0 | 2 | 0 | 0 | 0 | 2 | None | Orientalis | *Vibrio orientalis* | Yang et al. 1983 |
| *Vibrio ostreicida* | 4.37 | 0 | 0 | 0 | 2 | 0 | 0 | 0 | 2 | None |  | *Vibrio ostreicida* | Prado et al. 2014 |
| *Vibrio ouci* | 5.48 | 0 | 0 | 0 | 1 | 0 | 0 | 0 | 1 | None |  | *Vibrio ouci* | Li et al. 2020 |
| *Vibrio owensii* | 5.89 | 2 | 0 | 0 | 23 | 0 | 6 | 0 | 17 | None |  | *Vibrio owensii* | Cano-Gómez et al. 2010 |
| *Vibrio pacinii* | 4.30 | 0 | 0 | 0 | 1 | 0 | 0 | 0 | 1 | None |  | *Vibrio pacinii* | Gomez-Gil et al. 2003 |
| *Vibrio palustris* | 3.70 | 0 | 0 | 0 | 1 | 0 | 0 | 0 | 1 | None |  | *Vibrio palustris* | Lucena et al. 2017 |
| *Vibrio panuliri* | 4.74 | 0 | 0 | 0 | 4 | 0 | 1 | 0 | 3 | None |  | *Vibrio panuliri* | Kumari et al. 2015 |
| *Vibrio parahaemolyticus* | 5.17 | 2 | 0 | 0 | 1575 | 0 | 62 | 0 | 1513 | None | Harveyi | *Vibrio parahaemolyticus* | Fujino et al. 1951; Sakazaki et al. 1963 (Approved Lists 1980) |
| *Vibrio pectenicida* | 4.25 | 0 | 0 | 0 | 2 | 0 | 0 | 0 | 2 | None | Pectenicida | *Vibrio pectenicida* | Lambert et al. 1998 |
| *Vibrio penaeicida* | 6.57 | 0 | 0 | 2 | 2 | 0 | 0 | 2 | 2 | VII(2) | Nigripulchritudo | *Vibrio penaeicida* | Ishimaru et al. 1995 |
| *Vibrio plantisponsor* | 4.44 | 0 | 0 | 0 | 1 | 0 | 0 | 0 | 1 | None |  | *Vibrio plantisponsor* | Rameshkumar et al. 2012 |
| *Vibrio ponticus* | 4.80 | 2 | 1 | 0 | 4 | 0 | 1 | 0 | 3 | None | Scopthalmi | *Vibrio ponticus* | Macián et al. 2005 |
| *Vibrio profundi* | 5.19 | 0 | 0 | 0 | 1 | 0 | 0 | 0 | 1 | None |  | *"Vibrio profundi"* | Zhang et al. 2019 |
| *Vibrio proteolyticus* | 4.75 | 0 | 0 | 0 | 1 | 0 | 0 | 0 | 1 | None | Proteolyticus | *Vibrio proteolyticus* | Merkel et al. 1964; Baumann et al. 1982 |
| *Vibrio qinghaiensis* | 4.02 | 2 | 0 | 0 | 1 | 0 | 1 | 0 | 0 | None |  | None | - |
| *Vibrio quintilis* | 5.71 | 0 | 0 | 0 | 1 | 0 | 0 | 0 | 1 | None |  | *Vibrio quintilis* | Lucena et al. 2013 |
| *Vibrio renipiscarius* | 4.34 | 0 | 0 | 0 | 2 | 0 | 0 | 0 | 2 | None |  | *Vibrio renipiscarius* | Tarazona et al. 2015 |
| *Vibrio rhizosphaerae* | 4.46 | 0 | 0 | 0 | 1 | 0 | 0 | 0 | 1 | None | Gazogenes | *Vibrio rhizosphaerae* | Ramesh Kumar and Nair 2007 |
| *Vibrio rhodolitus* | 4.56 | 0 | 0 | 0 | 1 | 0 | 0 | 0 | 1 | None |  | *"Vibrio rhodolitus"* | Silva et al. 2018 |
| *Vibrio rotiferianus* | 5.28 | 2 | 0 | 0 | 14 | 0 | 2 | 0 | 12 | None | Harveyi | *Vibrio rotiferianus* | Gomez-Gil et al. 2003 |
| *Vibrio ruber* | 4.62 | 0 | 0 | 0 | 1 | 0 | 0 | 0 | 1 | None | Gazogenes | *Vibrio ruber* | Shieh et al. 2003 |
| *Vibrio rumoiensis* | 4.21 | 2 | 2 | 2 | 2 | 1 | 1 | 1 | 1 | V(2) | Rumoiensis | *Vibrio rumoiensis* | Yumoto et al. 1999 |
| *Vibrio sagamiensis* | 4.48 | 0 | 0 | 0 | 2 | 0 | 0 | 0 | 2 | None |  | *Vibrio sagamiensis* | Yoshizawa et al. 2011 |
| *Vibrio salilacus* | 3.62 | 0 | 0 | 0 | 1 | 0 | 0 | 0 | 1 | None |  | *Vibrio salilacus* | Zhong et al. 2015 |
| *Vibrio scophthalmi* | 4.80 | 2 | 2 | 0 | 4 | 0 | 2 | 0 | 2 | None | Scopthalmi | *Vibrio scophthalmi* | Cerdà-Cuéllar et al. 1997 |
| *Vibrio sinaloensis* | 4.54 | 0 | 0 | 0 | 5 | 0 | 0 | 0 | 5 | None | Orientalis | *Vibrio sinaloensis* | Gomez-Gil et al. 2008 |
| *Vibrio sinensis* | 5.30 | 0 | 0 | 0 | 1 | 0 | 0 | 0 | 1 | None |  | *Vibrio sinensis* | Li et al. 2020 |
| *Vibrio sonorensis* | 4.77 | 0 | 0 | 0 | 1 | 0 | 0 | 0 | 1 | None |  | *Vibrio sonorensis* | González-Castillo et al. 2017 |
| *Vibrio spartinae* | 5.01 | 2 | 0 | 0 | 2 | 0 | 1 | 0 | 1 | None |  | *Vibrio spartinae* | Lucena et al. 2017 |
| *Vibrio splendidus* | 5.54 | 0 | 0 | 5 | 110 | 0 | 1 | 5 | 109 | I(5) | Splendidus | *Vibrio splendidus* | Beijerinck 1900; Baumann et al. 1981 |
| *Vibrio superstes* | 4.72 | 0 | 0 | 0 | 2 | 0 | 0 | 0 | 2 | None | Halioticoli | *Vibrio superstes* | Hayashi et al. 2003 |
| *Vibrio taketomensis* | 4.36 | 2 | 1 | 0 | 2 | 0 | 2 | 0 | 0 | None |  | *"Vibrio taketomensis"* | Tanaka et al. 2020 |
| *Vibrio tapetis* | 5.73 | 2 | 1 | 0 | 1 | 0 | 1 | 0 | 0 | None | Tapetis | *Vibrio tapetis* | Borrego et al. 1996 |
| *Vibrio tasmaniensis* | 4.83 | 0 | 0 | 2 | 23 | 0 | 0 | 2 | 23 | I(2) | Splendidus | *Vibrio tasmaniensis* | Thompson et al. 2003 |
| *Vibrio tetraodonis* | 4.36 | 0 | 0 | 0 | 1 | 0 | 0 | 0 | 1 | None |  | *"Vibrio tetraodonis"* | Azevedo et al. 2021 |
| *Vibrio thalassae* | 5.11 | 0 | 0 | 1 | 1 | 0 | 0 | 1 | 1 | II(1) |  | *Vibrio thalassae* | Tarazona et al. 2014 |
| *Vibrio toranzoniae* | 4.56 | 0 | 0 | 0 | 7 | 0 | 0 | 0 | 7 | None |  | *Vibrio toranzoniae* | Lasa et al. 2013 |
| *Vibrio tritonius* | 5.22 | 2 | 0 | 0 | 1 | 0 | 1 | 0 | 0 | None | Porteresiae | *Vibrio tritonius* | Sawabe et al. 2015 |
| *Vibrio tubiashii* | 5.54 | 2 | 4 | 0 | 5 | 0 | 1 | 0 | 4 | None | Orientalis | *Vibrio tubiashii* | Hada et al. 1984 |
| *Vibrio variabilis* | 5.80 | 0 | 0 | 0 | 2 | 0 | 0 | 0 | 2 | None | Mediterranei | *Vibrio variabilis* | Chimetto et al. 2011 |
| *Vibrio viridaestus* | 4.71 | 0 | 0 | 0 | 1 | 0 | 0 | 0 | 1 | None |  | *Vibrio viridaestus* | Li et al. 2020 |
| *Vibrio vulnificus* | 4.97 | 2 | 0 | 2 | 226 | 1 | 23 | 1 | 203 | IX(2) | Vulnificus | *Vibrio vulnificus* | Reichelt et al. 1979; Farmer 1980 |
| *Vibrio xiamenensis* | 5.50 | 0 | 0 | 0 | 1 | 0 | 0 | 0 | 1 | None |  | *Vibrio xiamenensis* | Gao et al. 2012 |
| *Vibrio xuii* | 6.30 | 0 | 0 | 0 | 1 | 0 | 0 | 0 | 1 | None | Nereis | *Vibrio xuii* | Thompson et al. 2003 |
| *Vibrio zhugei* | 4.01 | 2 | 0 | 0 | 1 | 0 | 1 | 0 | 0 | None |  | *Vibrio zhugei* | Guo et al. 2019 |
| *Vibrio ziniensis* | 4.65 | 2 | 0 | 0 | 1 | 0 | 1 | 0 | 0 | None |  | *Vibrio ziniensis* | Wu et al. 2021 |

**Table S5. Significant up- or down- regulated genes in the transcriptomes of strains WXL032, YSX02 and WXL531 incubated with phosphate (Pho)/MPn.**

| Isolate_ID | Gene_ID | Gene name | FC_MPn/Pho | Log2_FC (MPn/Pho) | P-value | P-adjust | Regulate | Pho_mean TPM | MPn_mean TPM |
| --- | --- | --- | --- | --- | --- | --- | --- | --- | --- |
| WXL531 | BSZ05_RS01265 | BSZ05_RS01265 | 2.140 | 1.097 | 5.89E-04 | 4.86E-02 | up | 123.03 | 260.76 |
|  | BSZ05_RS03305 | *phoB* | 0.316 | -1.663 | 1.11E-06 | 1.55E-04 | down | 16.21 | 4.85 |
|  | BSZ05_RS06415 | BSZ05_RS06415 | 0.343 | -1.545 | 4.71E-06 | 5.72E-04 | down | 161.73 | 52.74 |
|  | BSZ05_RS08140 | *cobA* | 0.412 | -1.281 | 3.41E-04 | 2.97E-02 | down | 4.87 | 1.92 |
|  | BSZ05_RS08145 | *cysD* | 0.464 | -1.108 | 9.81E-05 | 9.63E-03 | down | 13.40 | 6.08 |
|  | BSZ05_RS08150 | *cysN* | 0.469 | -1.091 | 9.50E-05 | 9.52E-03 | down | 9.10 | 4.24 |
|  | BSZ05_RS09185 | BSZ05_RS09185 | 0.287 | -1.802 | 4.57E-04 | 3.90E-02 | down | 3.18 | 0.88 |
|  | BSZ05_RS09285 | BSZ05_RS09285 | 13.283 | 3.731 | 4.83E-07 | 7.43E-05 | up | 0.05 | 0.74 |
|  | BSZ05_RS09925 | *galM* | 2.371 | 1.245 | 3.12E-04 | 2.77E-02 | up | 2.26 | 5.29 |
|  | BSZ05_RS09975 | *asnB* | 0.204 | -2.291 | 2.56E-05 | 2.88E-03 | down | 38.99 | 7.47 |
|  | BSZ05_RS11060 | BSZ05_RS11060 | 2.560 | 1.356 | 3.19E-05 | 3.44E-03 | up | 234.89 | 601.13 |
|  | BSZ05_RS12630 | *metE* | 0.337 | -1.567 | 8.58E-06 | 9.89E-04 | down | 48.42 | 15.33 |
|  | BSZ05_RS13015 | *ugpB* | 9.080 | 3.183 | 2.48E-10 | 6.36E-08 | up | 0.23 | 2.07 |
|  | BSZ05_RS13120 | *glpD* | 4.184 | 2.065 | 6.17E-07 | 8.89E-05 | up | 36.34 | 141.93 |
|  | BSZ05_RS13565 | BSZ05_RS13565 | 0.372 | -1.428 | 2.51E-04 | 2.32E-02 | down | 12.47 | 4.42 |
|  | BSZ05_RS13570 | *melB* | 0.317 | -1.655 | 2.92E-06 | 3.64E-04 | down | 9.85 | 2.99 |
|  | BSZ05_RS15700 | BSZ05_RS15700 | 2.843 | 1.508 | 9.29E-05 | 9.52E-03 | up | 64.71 | 169.50 |
|  | BSZ05_RS17720 | *potA* | 6.259 | 2.646 | 5.91E-07 | 8.79E-05 | up | 0.32 | 2.12 |
|  | BSZ05_RS17725 | *potC* | 49.372 | 5.626 | 3.68E-09 | 7.37E-07 | up | 0.03 | 1.74 |
|  | BSZ05_RS17730 | BSZ05_RS17730 | 16.641 | 4.057 | 1.68E-09 | 3.88E-07 | up | 0.09 | 1.48 |
|  | BSZ05_RS17735 | BSZ05_RS17735 | 53.732 | 5.748 | 2.37E-19 | 1.36E-16 | up | 0.09 | 4.88 |
|  | BSZ05_RS17740 | BSZ05_RS17740 | 97.961 | 6.614 | 1.13E-34 | 1.73E-31 | up | 0.11 | 11.13 |
|  | BSZ05_RS18005 | ***phnF*** | 122.862 | 6.941 | 7.59E-18 | 3.50E-15 | up | 0.05 | 6.64 |
|  | BSZ05_RS18010 | ***phnG*** | 408.508 | 8.674 | 1.72E-12 | 5.68E-10 | up | 0.03 | 11.03 |
|  | BSZ05_RS18015 | ***phnH*** | 202.996 | 7.665 | 5.40E-21 | 3.56E-18 | up | 0.06 | 12.36 |
|  | BSZ05_RS18020 | ***phnI*** | 133.303 | 7.059 | 1.76E-43 | 8.10E-40 | up | 0.12 | 16.58 |
|  | BSZ05_RS18025 | ***phnJ*** | 107.114 | 6.743 | 7.97E-38 | 1.84E-34 | up | 0.17 | 18.66 |
|  | BSZ05_RS18030 | ***phnK*** | 125.574 | 6.972 | 2.28E-30 | 2.10E-27 | up | 0.10 | 13.34 |
|  | BSZ05_RS18035 | ***phnL*** | 61.239 | 5.936 | 3.11E-27 | 2.39E-24 | up | 0.17 | 10.62 |
|  | BSZ05_RS18040 | ***phnM*** | 87.659 | 6.454 | 2.00E-32 | 2.30E-29 | up | 0.20 | 17.11 |
|  | BSZ05_RS18045 | ***phnN*** | 26.180 | 4.710 | 2.82E-11 | 8.68E-09 | up | 0.12 | 3.21 |
|  | BSZ05_RS18050 | ***phnP*** | 50.126 | 5.647 | 1.34E-15 | 5.62E-13 | up | 0.08 | 4.23 |
|  | BSZ05_RS18055 | ***act*** | 241.029 | 7.913 | 2.08E-10 | 5.64E-08 | up | 0.02 | 3.83 |
|  | BSZ05_RS18255 | ***phnC*** | 38.293 | 5.259 | 2.58E-15 | 9.90E-13 | up | 0.07 | 2.72 |
|  | BSZ05_RS18260 | ***phnD*** | 7.060 | 2.820 | 2.94E-15 | 1.04E-12 | up | 1.06 | 7.45 |
|  | BSZ05_RS18265 | ***phnE*** | 38.753 | 5.276 | 3.82E-08 | 6.77E-06 | up | 0.03 | 1.21 |
|  | BSZ05_RS18270 | ***phnE*** | 11.536 | 3.528 | 6.11E-09 | 1.17E-06 | up | 0.16 | 1.86 |
|  | BSZ05_RS18490 | BSZ05_RS18490 | 6.793 | 2.764 | 2.69E-07 | 4.28E-05 | up | 10.48 | 81.76 |
|  | BSZ05_RS18495 | *malQ* | 6.426 | 2.684 | 1.80E-07 | 2.97E-05 | up | 5.84 | 42.92 |
|  | BSZ05_RS18980 | BSZ05_RS18980 | 0.361 | -1.469 | 7.71E-06 | 9.12E-04 | down | 4.86 | 1.77 |
|  | BSZ05_RS19535 | *phnX* | 3.501 | 1.808 | 2.67E-04 | 2.41E-02 | up | 0.77 | 2.82 |
|  | BSZ05_RS19540 | *puuE* | 5.646 | 2.497 | 2.24E-04 | 2.11E-02 | up | 0.20 | 1.20 |
|  | BSZ05_RS19545 | *phnW* | 7.934 | 2.988 | 6.81E-09 | 1.26E-06 | up | 0.19 | 1.41 |
|  | BSZ05_RS19555 | *phnT2* | 26.615 | 4.734 | 6.00E-08 | 1.02E-05 | up | 0.03 | 0.75 |
|  | BSZ05_RS20635 | *malK* | 4.770 | 2.254 | 2.13E-06 | 2.73E-04 | up | 4.62 | 24.08 |
|  | BSZ05_RS20640 | *malE* | 11.363 | 3.506 | 7.56E-11 | 2.18E-08 | up | 9.67 | 124.04 |
|  | BSZ05_RS20645 | *malF* | 11.341 | 3.503 | 3.39E-09 | 7.11E-07 | up | 1.91 | 24.74 |
|  | BSZ05_RS23095 | BSZ05_RS23095 | 3.665 | 1.874 | 3.21E-05 | 3.44E-03 | up | 0.54 | 2.07 |
|  | BSZ05_RS25620 | *pstC* | 4.828 | 2.271 | 1.68E-06 | 2.22E-04 | up | 0.34 | 1.62 |
|  | BSZ05_RS25625 | *pstS* | 7.210 | 2.850 | 6.79E-19 | 3.48E-16 | up | 2.14 | 15.23 |
|  | BSZ05_RS26160 | BSZ05_RS26160 | 3.563 | 1.833 | 1.17E-06 | 1.59E-04 | up | 0.34 | 1.20 |
|  | BSZ05_RS26250 | BSZ05_RS26250 | 2.806 | 1.489 | 1.44E-04 | 1.38E-02 | up | 2.71 | 7.81 |
|  | BSZ05_RS26255 | BSZ05_RS26255 | 4.654 | 2.218 | 2.51E-09 | 5.52E-07 | up | 5.45 | 26.73 |
| YSX02 | MNEHDJFA_00006 | *ibpA* | 0.153 | -2.709 | 2.01E-05 | 1.32E-03 | down | 4883.77 | 1412.97 |
|  | MNEHDJFA_00016 | *acuI* | 0.444 | -1.171 | 6.01E-05 | 2.92E-03 | down | 39.46 | 22.49 |
|  | MNEHDJFA_00034 | MNEHDJFA_00034 | 0.379 | -1.401 | 1.68E-03 | 2.95E-02 | down | 20.66 | 11.51 |
|  | MNEHDJFA_00036 | MNEHDJFA_00036 | 4.195 | 2.069 | 1.69E-04 | 5.76E-03 | up | 2.58 | 12.54 |
|  | MNEHDJFA_00042 | *gsiA* | 3.320 | 1.731 | 3.55E-04 | 9.62E-03 | up | 6.60 | 21.71 |
|  | MNEHDJFA_00043 | *dppA* | 3.338 | 1.739 | 3.49E-04 | 9.52E-03 | up | 12.79 | 41.44 |
|  | MNEHDJFA_00087 | *glnG* | 3.356 | 1.747 | 1.53E-03 | 2.80E-02 | up | 14.98 | 49.22 |
|  | MNEHDJFA_00088 | *glnL* | 4.405 | 2.139 | 2.48E-05 | 1.49E-03 | up | 6.00 | 26.99 |
|  | MNEHDJFA_00090 | *glnA* | 2.624 | 1.392 | 1.67E-03 | 2.94E-02 | up | 778.98 | 2359.12 |
|  | MNEHDJFA_00099 | *hslO* | 2.549 | 1.350 | 3.30E-03 | 4.68E-02 | up | 8.02 | 20.68 |
|  | MNEHDJFA_00118 | MNEHDJFA_00118 | 3.244 | 1.698 | 6.68E-05 | 3.11E-03 | up | 40.94 | 177.71 |
|  | MNEHDJFA_00138 | *rpmB* | 2.628 | 1.394 | 2.83E-03 | 4.27E-02 | up | 13.23 | 35.03 |
|  | MNEHDJFA_00151 | *pseB* | 0.458 | -1.127 | 2.27E-03 | 3.70E-02 | down | 7.98 | 4.84 |
|  | MNEHDJFA_00240 | *frdA* | 0.433 | -1.208 | 3.01E-03 | 4.46E-02 | down | 509.36 | 280.09 |
|  | MNEHDJFA_00281 | *rpsF* | 2.165 | 1.115 | 2.20E-03 | 3.62E-02 | up | 496.79 | 1269.09 |
|  | MNEHDJFA_00304 | MNEHDJFA_00304 | 3.028 | 1.598 | 2.39E-03 | 3.81E-02 | up | 2.13 | 10.07 |
|  | MNEHDJFA_00309 | MNEHDJFA_00309 | 4.366 | 2.126 | 6.83E-04 | 1.62E-02 | up | 0.60 | 3.04 |
|  | MNEHDJFA_00310 | MNEHDJFA_00310 | 3.887 | 1.959 | 1.20E-04 | 4.54E-03 | up | 1.91 | 9.70 |
|  | MNEHDJFA_00312 | MNEHDJFA_00312 | 5.614 | 2.489 | 3.80E-04 | 1.01E-02 | up | 0.23 | 1.41 |
|  | MNEHDJFA_00389 | *rpmA* | 2.673 | 1.419 | 2.67E-03 | 4.12E-02 | up | 38.08 | 121.28 |
|  | MNEHDJFA_00412 | MNEHDJFA_00412 | 0.214 | -2.222 | 4.52E-04 | 1.15E-02 | down | 2.75 | 0.73 |
|  | MNEHDJFA_00413 | *leuO* | 0.249 | -2.003 | 3.03E-05 | 1.68E-03 | down | 123.62 | 40.83 |
|  | MNEHDJFA_00415 | *ilvI* | 2.721 | 1.444 | 8.73E-06 | 7.37E-04 | up | 7.80 | 23.96 |
|  | MNEHDJFA_00458 | *petA* | 3.378 | 1.756 | 1.46E-03 | 2.72E-02 | up | 11.42 | 35.65 |
|  | MNEHDJFA_00515 | MNEHDJFA_00515 | 10.299 | 3.364 | 2.39E-07 | 5.00E-05 | up | 80.37 | 823.36 |
|  | MNEHDJFA_00516 | *ung* | 2.743 | 1.456 | 1.11E-03 | 2.20E-02 | up | 38.54 | 116.37 |
|  | MNEHDJFA_00546 | *rpsT* | 3.113 | 1.638 | 4.38E-04 | 1.13E-02 | up | 26.07 | 80.97 |
|  | MNEHDJFA_00580 | *clpB* | 0.433 | -1.207 | 8.92E-04 | 1.92E-02 | down | 38.50 | 21.57 |
|  | MNEHDJFA_00645 | *urea* | 2.941 | 1.556 | 1.65E-03 | 2.93E-02 | up | 5.17 | 17.19 |
|  | MNEHDJFA_00659 | *acp* | 2.122 | 1.085 | 1.57E-03 | 2.83E-02 | up | 3.36 | 8.33 |
|  | MNEHDJFA_00668 | MNEHDJFA_00668 | 0.282 | -1.827 | 3.31E-03 | 4.68E-02 | down | 5.07 | 1.96 |
|  | MNEHDJFA_00690 | *bamE* | 2.718 | 1.443 | 6.33E-04 | 1.54E-02 | up | 9.05 | 25.76 |
|  | MNEHDJFA_00691 | *recN* | 2.156 | 1.109 | 2.70E-03 | 4.14E-02 | up | 15.13 | 46.59 |
|  | MNEHDJFA_00694 | *gltT* | 5.428 | 2.440 | 3.02E-06 | 3.38E-04 | up | 0.61 | 3.87 |
|  | MNEHDJFA_00713 | MNEHDJFA_00713 | 0.168 | -2.572 | 1.85E-03 | 3.17E-02 | down | 98.21 | 33.72 |
|  | MNEHDJFA_00714 | MNEHDJFA_00714 | 0.129 | -2.950 | 4.25E-04 | 1.10E-02 | down | 408.32 | 120.15 |
|  | MNEHDJFA_00863 | *flaD* | 3.500 | 1.808 | 7.15E-06 | 6.84E-04 | up | 14.73 | 68.44 |
|  | MNEHDJFA_00864 | *flaD* | 3.884 | 1.958 | 2.54E-05 | 1.50E-03 | up | 2.77 | 13.15 |
|  | MNEHDJFA_00866 | MNEHDJFA_00866 | 0.357 | -1.485 | 1.25E-04 | 4.59E-03 | down | 533.88 | 282.94 |
|  | MNEHDJFA_00867 | MNEHDJFA_00867 | 0.397 | -1.332 | 3.87E-04 | 1.03E-02 | down | 44.74 | 25.86 |
|  | MNEHDJFA_00914 | *fadI* | 2.920 | 1.546 | 2.37E-05 | 1.46E-03 | up | 9.32 | 33.56 |
|  | MNEHDJFA_00936 | *dctP* | 7.057 | 2.819 | 7.17E-05 | 3.22E-03 | up | 0.33 | 2.27 |
|  | MNEHDJFA_01043 | *ahpC* | 0.373 | -1.421 | 1.19E-05 | 8.95E-04 | down | 17.10 | 8.10 |
|  | MNEHDJFA_01146 | *bioA* | 0.372 | -1.428 | 3.03E-03 | 4.46E-02 | down | 3.01 | 1.56 |
|  | MNEHDJFA_01183 | *torC* | 0.370 | -1.435 | 2.83E-04 | 8.40E-03 | down | 12.55 | 6.44 |
|  | MNEHDJFA_01184 | *torA* | 0.388 | -1.367 | 1.15E-04 | 4.47E-03 | down | 63.58 | 35.63 |
|  | MNEHDJFA_01185 | MNEHDJFA_01185 | 0.177 | -2.501 | 2.10E-09 | 1.06E-06 | down | 50.30 | 10.36 |
|  | MNEHDJFA_01198 | *ttgD* | 2.445 | 1.290 | 1.33E-03 | 2.52E-02 | up | 3.26 | 8.57 |
|  | MNEHDJFA_01206 | MNEHDJFA_01206 | 2.565 | 1.359 | 5.92E-04 | 1.46E-02 | up | 4.54 | 12.37 |
|  | MNEHDJFA_01216 | MNEHDJFA_01216 | 0.353 | -1.502 | 1.31E-03 | 2.51E-02 | down | 6.90 | 2.83 |
|  | MNEHDJFA_01258 | MNEHDJFA_01258 | 4.144 | 2.051 | 1.38E-04 | 4.96E-03 | up | 4.10 | 17.90 |
|  | MNEHDJFA_01298 | MNEHDJFA_01298 | 2.488 | 1.315 | 3.19E-03 | 4.58E-02 | up | 21.98 | 72.26 |
|  | MNEHDJFA_01299 | MNEHDJFA_01299 | 3.047 | 1.608 | 3.49E-06 | 3.64E-04 | up | 3.10 | 12.09 |
|  | MNEHDJFA_01303 | MNEHDJFA_01303 | 3.357 | 1.747 | 3.95E-04 | 1.03E-02 | up | 2.83 | 11.06 |
|  | MNEHDJFA_01316 | MNEHDJFA_01316 | 0.101 | -3.306 | 2.89E-07 | 5.40E-05 | down | 10.74 | 2.32 |
|  | MNEHDJFA_01318 | MNEHDJFA_01318 | 5.382 | 2.428 | 1.85E-06 | 2.68E-04 | up | 12.33 | 68.96 |
|  | MNEHDJFA_01319 | *fadE* | 3.065 | 1.616 | 5.24E-05 | 2.62E-03 | up | 15.50 | 65.89 |
|  | MNEHDJFA_01354 | *sdaA* | 0.298 | -1.746 | 2.58E-03 | 4.01E-02 | down | 7.60 | 3.74 |
|  | MNEHDJFA_01362 | *ycjX* | 2.597 | 1.377 | 1.42E-04 | 5.01E-03 | up | 9.49 | 30.96 |
|  | MNEHDJFA_01363 | MNEHDJFA_01363 | 2.794 | 1.482 | 4.23E-05 | 2.21E-03 | up | 4.47 | 15.59 |
|  | MNEHDJFA_01425 | MNEHDJFA_01425 | 0.113 | -3.140 | 1.92E-05 | 1.31E-03 | down | 15.00 | 3.90 |
|  | MNEHDJFA_01544 | MNEHDJFA_01544 | 0.341 | -1.551 | 2.97E-03 | 4.44E-02 | down | 0.67 | 0.31 |
|  | MNEHDJFA_01555 | *syrM1* | 0.178 | -2.489 | 8.56E-05 | 3.57E-03 | down | 2.03 | 0.42 |
|  | MNEHDJFA_01575 | MNEHDJFA_01575 | 0.257 | -1.959 | 8.09E-04 | 1.78E-02 | down | 7.58 | 2.45 |
|  | MNEHDJFA_01646 | *artM* | 0.312 | -1.681 | 2.48E-03 | 3.91E-02 | down | 16.89 | 7.02 |
|  | MNEHDJFA_01665 | MNEHDJFA_01665 | 0.280 | -1.839 | 2.71E-03 | 4.14E-02 | down | 1.04 | 0.31 |
|  | MNEHDJFA_01698 | *mcpP* | 0.415 | -1.269 | 3.23E-03 | 4.63E-02 | down | 23.95 | 13.36 |
|  | MNEHDJFA_01799 | *prpB* | 7.016 | 2.811 | 9.66E-06 | 7.79E-04 | up | 0.48 | 3.80 |
|  | MNEHDJFA_01807 | MNEHDJFA_01807 | 3.072 | 1.619 | 7.02E-04 | 1.63E-02 | up | 18.86 | 59.88 |
|  | MNEHDJFA_01824 | MNEHDJFA_01824 | 3.829 | 1.937 | 1.80E-03 | 3.13E-02 | up | 0.96 | 3.41 |
|  | MNEHDJFA_01830 | MNEHDJFA_01830 | 0.252 | -1.991 | 9.63E-06 | 7.79E-04 | down | 214.44 | 69.13 |
|  | MNEHDJFA_01838 | MNEHDJFA_01838 | 0.306 | -1.707 | 8.46E-05 | 3.57E-03 | down | 34.53 | 12.04 |
|  | MNEHDJFA_01862 | *ydeA* | 0.288 | -1.797 | 2.06E-03 | 3.45E-02 | down | 4.45 | 2.16 |
|  | MNEHDJFA_01898 | *tnaA* | 0.219 | -2.193 | 9.19E-05 | 3.71E-03 | down | 36.70 | 11.78 |
|  | MNEHDJFA_01971 | MNEHDJFA_01971 | 4.706 | 2.235 | 8.72E-09 | 3.44E-06 | up | 20.32 | 130.37 |
|  | MNEHDJFA_01978 | *sdcS* | 6.191 | 2.630 | 6.39E-05 | 3.06E-03 | up | 1.92 | 10.53 |
|  | MNEHDJFA_01982 | MNEHDJFA_01982 | 0.394 | -1.344 | 3.16E-03 | 4.57E-02 | down | 6.08 | 3.65 |
|  | MNEHDJFA_02008 | MNEHDJFA_02008 | 6.339 | 2.664 | 1.99E-04 | 6.55E-03 | up | 0.26 | 1.61 |
|  | MNEHDJFA_02010 | MNEHDJFA_02010 | 0.400 | -1.320 | 2.37E-03 | 3.80E-02 | down | 3.89 | 2.01 |
|  | MNEHDJFA_02031 | *nrdA* | 2.373 | 1.246 | 2.15E-04 | 6.79E-03 | up | 5.99 | 15.73 |
|  | MNEHDJFA_02035 | *dmsD* | 0.396 | -1.336 | 2.44E-03 | 3.86E-02 | down | 5.34 | 2.63 |
|  | MNEHDJFA_02069 | MNEHDJFA_02069 | 0.232 | -2.107 | 2.54E-07 | 5.00E-05 | down | 26.16 | 8.82 |
|  | MNEHDJFA_02070 | MNEHDJFA_02070 | 0.220 | -2.182 | 7.43E-06 | 6.84E-04 | down | 24.28 | 7.93 |
|  | MNEHDJFA_02098 | *dadA1* | 4.258 | 2.090 | 1.03E-05 | 8.16E-04 | up | 1.03 | 5.14 |
|  | MNEHDJFA_02163 | *icd2* | 2.199 | 1.137 | 3.03E-03 | 4.46E-02 | up | 11.94 | 29.16 |
|  | MNEHDJFA_02244 | *oppA* | 2.882 | 1.527 | 1.22E-04 | 4.54E-03 | up | 9.82 | 29.15 |
|  | MNEHDJFA_02280 | *mleN* | 0.367 | -1.447 | 2.98E-03 | 4.44E-02 | down | 3.86 | 1.93 |
|  | MNEHDJFA_02294 | *fadH* | 2.201 | 1.138 | 3.38E-03 | 4.76E-02 | up | 10.79 | 30.41 |
|  | MNEHDJFA_02319 | *cvpA* | 2.926 | 1.549 | 2.34E-03 | 3.78E-02 | up | 3.91 | 11.49 |
|  | MNEHDJFA_02364 | *fadD* | 2.125 | 1.087 | 1.91E-04 | 6.34E-03 | up | 5.35 | 14.16 |
|  | MNEHDJFA_02391 | *gltA* | 2.610 | 1.384 | 2.13E-03 | 3.53E-02 | up | 29.91 | 80.61 |
|  | MNEHDJFA_02418 | MNEHDJFA_02418 | 0.208 | -2.268 | 7.51E-04 | 1.71E-02 | down | 3.39 | 0.84 |
|  | MNEHDJFA_02437 | *flaD* | 3.415 | 1.772 | 2.09E-05 | 1.35E-03 | up | 6.04 | 26.10 |
|  | MNEHDJFA_02459 | *pyrP* | 2.584 | 1.370 | 1.53E-03 | 2.80E-02 | up | 3.45 | 9.21 |
|  | MNEHDJFA_02461 | *purM* | 2.472 | 1.306 | 8.20E-04 | 1.80E-02 | up | 8.07 | 22.39 |
|  | MNEHDJFA_02502 | MNEHDJFA_02502 | 4.270 | 2.094 | 7.34E-04 | 1.68E-02 | up | 0.99 | 4.65 |
|  | MNEHDJFA_02512 | MNEHDJFA_02512 | 4.202 | 2.071 | 1.98E-05 | 1.32E-03 | up | 2.83 | 15.04 |
|  | MNEHDJFA_02533 | MNEHDJFA_02533 | 2.569 | 1.361 | 1.44E-03 | 2.70E-02 | up | 1.73 | 5.04 |
|  | MNEHDJFA_02539 | MNEHDJFA_02539 | 0.320 | -1.646 | 2.62E-03 | 4.06E-02 | down | 46.72 | 23.93 |
|  | MNEHDJFA_02540 | *gcvA* | 0.344 | -1.538 | 3.27E-04 | 9.20E-03 | down | 47.63 | 22.30 |
|  | MNEHDJFA_02576 | *galE* | 0.419 | -1.253 | 3.07E-04 | 8.93E-03 | down | 6.36 | 3.32 |
|  | MNEHDJFA_02578 | *ebgA* | 0.221 | -2.177 | 7.86E-04 | 1.76E-02 | down | 0.26 | 0.07 |
|  | MNEHDJFA_02614 | MNEHDJFA_02614 | 2.707 | 1.437 | 1.93E-03 | 3.28E-02 | up | 1.60 | 4.66 |
|  | MNEHDJFA_02648 | *rbfA* | 0.430 | -1.219 | 3.10E-04 | 8.93E-03 | down | 85.05 | 48.42 |
|  | MNEHDJFA_02688 | *amtB* | 3.266 | 1.708 | 1.22E-03 | 2.36E-02 | up | 95.73 | 317.08 |
|  | MNEHDJFA_02689 | *glnB* | 3.495 | 1.805 | 3.29E-04 | 9.20E-03 | up | 7.29 | 28.25 |
|  | MNEHDJFA_02691 | *acnB* | 2.268 | 1.181 | 6.56E-04 | 1.57E-02 | up | 20.79 | 53.93 |
|  | MNEHDJFA_02794 | MNEHDJFA_02794 | 0.307 | -1.703 | 2.84E-04 | 8.40E-03 | down | 7.90 | 3.35 |
|  | MNEHDJFA_02863 | *arcA* | 3.212 | 1.684 | 2.44E-06 | 3.10E-04 | up | 2.40 | 8.84 |
|  | MNEHDJFA_02898 | MNEHDJFA_02898 | 0.415 | -1.269 | 1.85E-03 | 3.17E-02 | down | 44.03 | 24.14 |
|  | MNEHDJFA_02921 | MNEHDJFA_02921 | 0.274 | -1.869 | 7.88E-04 | 1.76E-02 | down | 3.60 | 1.12 |
|  | MNEHDJFA_02929 | MNEHDJFA_02929 | 3.503 | 1.809 | 8.19E-06 | 7.26E-04 | up | 7.98 | 32.11 |
|  | MNEHDJFA_02981 | *cfxP* | 2.255 | 1.173 | 7.63E-04 | 1.73E-02 | up | 4.49 | 12.81 |
|  | MNEHDJFA_03000 | MNEHDJFA_03000 | 0.222 | -2.173 | 3.17E-03 | 4.58E-02 | down | 0.65 | 0.18 |
|  | MNEHDJFA_03024 | *menA* | 0.371 | -1.430 | 7.30E-05 | 3.24E-03 | down | 29.64 | 15.13 |
|  | MNEHDJFA_03031 | MNEHDJFA_03031 | 3.430 | 1.778 | 1.05E-03 | 2.13E-02 | up | 1.80 | 7.20 |
|  | MNEHDJFA_03042 | *aspA* | 0.284 | -1.817 | 2.39E-05 | 1.46E-03 | down | 147.64 | 53.66 |
|  | MNEHDJFA_03044 | MNEHDJFA_03044 | 0.281 | -1.831 | 2.87E-03 | 4.31E-02 | down | 1.70 | 0.73 |
|  | MNEHDJFA_03047 | *arcC2* | 0.310 | -1.688 | 9.20E-05 | 3.71E-03 | down | 4.62 | 1.89 |
|  | MNEHDJFA_03051 | *argR* | 0.179 | -2.482 | 1.96E-06 | 2.68E-04 | down | 23.22 | 6.64 |
|  | MNEHDJFA_03058 | *fumC* | 0.436 | -1.198 | 1.84E-03 | 3.17E-02 | down | 22.12 | 10.69 |
|  | MNEHDJFA_03068 | *dusB* | 2.251 | 1.171 | 1.39E-03 | 2.63E-02 | up | 31.01 | 79.04 |
|  | MNEHDJFA_03071 | *purH* | 2.481 | 1.311 | 1.61E-04 | 5.54E-03 | up | 7.57 | 20.68 |
|  | MNEHDJFA_03072 | *purD* | 2.380 | 1.251 | 1.49E-03 | 2.76E-02 | up | 6.35 | 16.04 |
|  | MNEHDJFA_03125 | *sthA* | 3.526 | 1.818 | 9.68E-04 | 2.02E-02 | up | 1.63 | 5.57 |
|  | MNEHDJFA_03150 | *gap3* | 0.320 | -1.644 | 8.34E-05 | 3.57E-03 | down | 55.50 | 27.58 |
|  | MNEHDJFA_03190 | MNEHDJFA_03190 | 2.235 | 1.160 | 3.91E-04 | 1.03E-02 | up | 3.91 | 10.35 |
|  | MNEHDJFA_03235 | *purE* | 3.221 | 1.687 | 1.74E-05 | 1.21E-03 | up | 5.24 | 19.80 |
|  | MNEHDJFA_03236 | *purK* | 2.413 | 1.271 | 8.96E-05 | 3.70E-03 | up | 4.93 | 13.92 |
|  | MNEHDJFA_03254 | MNEHDJFA_03254 | 3.680 | 1.880 | 3.79E-05 | 2.07E-03 | up | 1.56 | 7.10 |
|  | MNEHDJFA_03313 | MNEHDJFA_03313 | 3.336 | 1.738 | 1.45E-03 | 2.71E-02 | up | 0.81 | 2.80 |
|  | MNEHDJFA_03331 | MNEHDJFA_03331 | 0.426 | -1.230 | 2.63E-04 | 7.97E-03 | down | 14.83 | 7.59 |
|  | MNEHDJFA_03334 | *rcsC* | 0.283 | -1.823 | 3.88E-08 | 1.25E-05 | down | 3.97 | 1.40 |
|  | MNEHDJFA_03335 | *hlyD* | 0.371 | -1.432 | 2.38E-04 | 7.28E-03 | down | 5.64 | 2.59 |
|  | MNEHDJFA_03337 | MNEHDJFA_03337 | 0.285 | -1.810 | 3.10E-06 | 3.38E-04 | down | 308.18 | 123.76 |
|  | MNEHDJFA_03342 | MNEHDJFA_03342 | 3.400 | 1.766 | 1.71E-05 | 1.21E-03 | up | 2.80 | 10.60 |
|  | MNEHDJFA_03370 | *glyA2* | 2.527 | 1.338 | 2.04E-04 | 6.63E-03 | up | 2.84 | 9.08 |
|  | MNEHDJFA_03371 | MNEHDJFA_03371 | 3.446 | 1.785 | 8.55E-05 | 3.57E-03 | up | 1.30 | 5.90 |
|  | MNEHDJFA_03376 | *arfB* | 4.138 | 2.049 | 1.42E-04 | 5.01E-03 | up | 1.13 | 5.75 |
|  | MNEHDJFA_03379 | *fhs* | 2.972 | 1.571 | 9.76E-07 | 1.65E-04 | up | 1.64 | 5.98 |
|  | MNEHDJFA_03408 | MNEHDJFA_03408 | 0.216 | -2.211 | 6.54E-04 | 1.57E-02 | down | 12.82 | 4.13 |
|  | MNEHDJFA_03427 | MNEHDJFA_03427 | 5.004 | 2.323 | 6.59E-05 | 3.11E-03 | up | 0.54 | 3.23 |
|  | MNEHDJFA_03440 | MNEHDJFA_03440 | 2.208 | 1.142 | 2.83E-03 | 4.27E-02 | up | 2.22 | 5.55 |
|  | MNEHDJFA_03495 | MNEHDJFA_03495 | 8.731 | 3.126 | 7.64E-09 | 3.39E-06 | up | 5.28 | 60.56 |
|  | MNEHDJFA_03496 | MNEHDJFA_03496 | 2.856 | 1.514 | 6.93E-04 | 1.63E-02 | up | 2.14 | 7.53 |
|  | MNEHDJFA_03503 | *nrdD* | 2.895 | 1.534 | 1.57E-03 | 2.83E-02 | up | 3.26 | 12.10 |
|  | MNEHDJFA_03582 | *mngB* | 0.100 | -3.323 | 8.50E-06 | 7.36E-04 | down | 0.38 | 0.07 |
|  | MNEHDJFA_03670 | *rhaS* | 0.165 | -2.598 | 1.56E-05 | 1.15E-03 | down | 1.75 | 0.31 |
|  | MNEHDJFA_03685 | MNEHDJFA_03685 | 11.923 | 3.576 | 1.10E-06 | 1.78E-04 | up | 1.51 | 19.21 |
|  | MNEHDJFA_03686 | *cusA* | 10.939 | 3.451 | 5.20E-08 | 1.42E-05 | up | 4.37 | 48.83 |
|  | MNEHDJFA_03687 | MNEHDJFA_03687 | 7.825 | 2.968 | 2.58E-05 | 1.50E-03 | up | 1.52 | 11.29 |
|  | MNEHDJFA_03688 | MNEHDJFA_03688 | 9.282 | 3.214 | 6.39E-06 | 6.48E-04 | up | 1.33 | 11.19 |
|  | MNEHDJFA_03689 | MNEHDJFA_03689 | 9.702 | 3.278 | 1.61E-05 | 1.17E-03 | up | 2.34 | 22.24 |
|  | MNEHDJFA_03699 | *echA8* | 3.035 | 1.602 | 1.23E-03 | 2.36E-02 | up | 3.70 | 11.51 |
|  | MNEHDJFA_03703 | *rhaR* | 3.006 | 1.588 | 1.58E-03 | 2.84E-02 | up | 1.33 | 4.18 |
|  | MNEHDJFA_03782 | MNEHDJFA_03782 | 5.577 | 2.479 | 2.23E-05 | 1.41E-03 | up | 0.49 | 2.76 |
|  | MNEHDJFA_03793 | MNEHDJFA_03793 | 2.423 | 1.277 | 3.47E-04 | 9.52E-03 | up | 1.24 | 3.60 |
|  | MNEHDJFA_03817 | *guaC* | 3.431 | 1.778 | 2.84E-06 | 3.36E-04 | up | 4.50 | 17.20 |
|  | MNEHDJFA_03846 | *bepF* | 6.341 | 2.665 | 5.69E-05 | 2.80E-03 | up | 0.16 | 1.52 |
|  | MNEHDJFA_03894 | MNEHDJFA_03894 | 3.232 | 1.693 | 6.80E-04 | 1.62E-02 | up | 62.21 | 232.56 |
|  | MNEHDJFA_03906 | *chuW* | 0.341 | -1.552 | 7.10E-05 | 3.22E-03 | down | 12.84 | 4.87 |
|  | MNEHDJFA_03907 | MNEHDJFA_03907 | 0.400 | -1.323 | 7.04E-04 | 1.63E-02 | down | 4.57 | 2.16 |
|  | MNEHDJFA_03908 | *tolQ* | 0.468 | -1.094 | 2.17E-03 | 3.58E-02 | down | 8.29 | 4.56 |
|  | MNEHDJFA_03909 | *tolR* | 0.187 | -2.419 | 1.61E-03 | 2.86E-02 | down | 3.32 | 0.75 |
|  | MNEHDJFA_03910 | *hmuT* | 0.354 | -1.497 | 3.14E-06 | 3.38E-04 | down | 13.27 | 6.08 |
|  | MNEHDJFA_03912 | *hmuV* | 0.352 | -1.506 | 1.23E-04 | 4.54E-03 | down | 8.17 | 3.18 |
|  | MNEHDJFA_04005 | MNEHDJFA_04005 | 0.276 | -1.855 | 9.98E-04 | 2.06E-02 | down | 1.14 | 0.41 |
|  | MNEHDJFA_04010 | *hlyC* | 4.421 | 2.144 | 1.09E-04 | 4.33E-03 | up | 1.11 | 5.03 |
|  | MNEHDJFA_04020 | *yiaW* | 2.876 | 1.524 | 9.19E-04 | 1.94E-02 | up | 3.62 | 11.77 |
|  | MNEHDJFA_04021 | *yiaV* | 2.333 | 1.222 | 3.15E-04 | 9.02E-03 | up | 3.90 | 10.86 |
|  | MNEHDJFA_04084 | *ulaE* | 0.288 | -1.796 | 1.47E-04 | 5.10E-03 | down | 28.89 | 11.65 |
|  | MNEHDJFA_04085 | *sgbH* | 0.303 | -1.723 | 6.82E-06 | 6.72E-04 | down | 8.94 | 3.49 |
|  | MNEHDJFA_04086 | *ybhA* | 0.262 | -1.933 | 1.80E-04 | 6.07E-03 | down | 13.18 | 4.95 |
|  | MNEHDJFA_04087 | *ulaF* | 0.212 | -2.240 | 1.13E-05 | 8.70E-04 | down | 25.46 | 9.06 |
|  | MNEHDJFA_04115 | *acr3* | 0.293 | -1.773 | 1.15E-03 | 2.24E-02 | down | 4.44 | 2.17 |
|  | MNEHDJFA_04185 | *uxaC* | 0.219 | -2.190 | 5.46E-04 | 1.37E-02 | down | 2.46 | 0.67 |
|  | MNEHDJFA_04210 | *ectB* | 4.449 | 2.154 | 8.10E-04 | 1.78E-02 | up | 0.36 | 1.59 |
|  | MNEHDJFA_04211 | *ectA* | 4.621 | 2.208 | 8.45E-05 | 3.57E-03 | up | 4.35 | 18.85 |
|  | MNEHDJFA_04249 | *dctD* | 3.251 | 1.701 | 3.09E-03 | 4.51E-02 | up | 0.33 | 1.16 |
|  | MNEHDJFA_04268 | MNEHDJFA_04268 | 5.063 | 2.340 | 2.97E-04 | 8.70E-03 | up | 0.37 | 1.99 |
|  | MNEHDJFA_04281 | MNEHDJFA_04281 | 2.237 | 1.162 | 1.76E-03 | 3.08E-02 | up | 8.60 | 20.95 |
|  | MNEHDJFA_04302 | MNEHDJFA_04302 | 2.858 | 1.515 | 6.33E-04 | 1.54E-02 | up | 26.00 | 82.94 |
|  | MNEHDJFA_04308 | *tauR* | 6.588 | 2.720 | 1.95E-10 | 1.77E-07 | up | 5.33 | 35.69 |
|  | MNEHDJFA_04352 | *rlmJ* | 2.658 | 1.410 | 1.12E-03 | 2.20E-02 | up | 2.28 | 7.03 |
|  | MNEHDJFA_04388 | *mtlD* | 0.455 | -1.135 | 2.12E-04 | 6.79E-03 | down | 9.08 | 5.10 |
|  | MNEHDJFA_04401 | MNEHDJFA_04401 | 4.155 | 2.055 | 1.12E-04 | 4.41E-03 | up | 5.46 | 26.99 |
|  | MNEHDJFA_04441 | MNEHDJFA_04441 | 2.657 | 1.410 | 8.99E-04 | 1.92E-02 | up | 3.21 | 9.26 |
|  | MNEHDJFA_04450 | *lacF* | 0.203 | -2.302 | 3.33E-04 | 9.22E-03 | down | 0.94 | 0.23 |
|  | MNEHDJFA_04451 | *araQ* | 0.308 | -1.697 | 9.06E-04 | 1.92E-02 | down | 2.11 | 0.83 |
|  | MNEHDJFA_04530 | *xdhA* | 0.441 | -1.183 | 1.01E-03 | 2.07E-02 | down | 13.05 | 7.80 |
|  | MNEHDJFA_04547 | MNEHDJFA_04547 | 0.204 | -2.293 | 1.08E-03 | 2.16E-02 | down | 1.66 | 0.62 |
|  | MNEHDJFA_04564 | MNEHDJFA_04564 | 10.762 | 3.428 | 2.81E-06 | 3.36E-04 | up | 0.25 | 2.88 |
|  | MNEHDJFA_04613 | *comR* | 0.324 | -1.625 | 2.05E-03 | 3.44E-02 | down | 17.88 | 9.84 |
|  | MNEHDJFA_04625 | MNEHDJFA_04625 | 0.063 | -3.987 | 1.32E-06 | 2.03E-04 | down | 11.00 | 1.55 |
|  | MNEHDJFA_04641 | *ycjG* | 0.478 | -1.065 | 2.71E-04 | 8.16E-03 | down | 33.72 | 19.39 |
|  | MNEHDJFA_04653 | *glgC* | 2.844 | 1.508 | 4.58E-05 | 2.32E-03 | up | 58.75 | 189.95 |
|  | MNEHDJFA_04654 | *ompW* | 3.043 | 1.606 | 1.90E-03 | 3.24E-02 | up | 19.44 | 56.97 |
|  | MNEHDJFA_04661 | MNEHDJFA_04661 | 3.867 | 1.951 | 2.41E-06 | 3.10E-04 | up | 357.14 | 1669.87 |
|  | MNEHDJFA_04691 | ***phnC*** | 15.447 | 3.949 | 2.00E-10 | 1.77E-07 | up | 0.38 | 6.08 |
|  | MNEHDJFA_04692 | ***phnD*** | 5.060 | 2.339 | 3.25E-04 | 9.20E-03 | up | 0.87 | 4.28 |
|  | MNEHDJFA_04693 | ***phnE*** | 6.631 | 2.729 | 1.20E-04 | 4.54E-03 | up | 0.29 | 1.97 |
|  | MNEHDJFA_04694 | ***phnF*** | 6.594 | 2.721 | 1.86E-04 | 6.24E-03 | up | 0.21 | 1.67 |
|  | MNEHDJFA_04695 | ***phnG*** | 6.047 | 2.596 | 2.99E-05 | 1.68E-03 | up | 0.96 | 6.14 |
|  | MNEHDJFA_04696 | ***phnH*** | 9.065 | 3.180 | 1.46E-08 | 5.18E-06 | up | 0.71 | 7.00 |
|  | MNEHDJFA_04697 | ***phnI*** | 8.088 | 3.016 | 2.95E-10 | 2.10E-07 | up | 0.98 | 8.62 |
|  | MNEHDJFA_04698 | ***phnJ*** | 3.872 | 1.953 | 1.21E-04 | 4.54E-03 | up | 2.15 | 8.83 |
|  | MNEHDJFA_04699 | ***phnK*** | 5.326 | 2.413 | 1.22E-07 | 3.09E-05 | up | 0.99 | 6.66 |
|  | MNEHDJFA_04700 | ***phnL*** | 5.659 | 2.501 | 2.53E-07 | 5.00E-05 | up | 1.12 | 6.96 |
|  | MNEHDJFA_04701 | ***phnM*** | 6.257 | 2.646 | 4.62E-10 | 2.73E-07 | up | 2.32 | 16.60 |
|  | MNEHDJFA_04702 | ***phnN*** | 5.373 | 2.426 | 4.40E-08 | 1.30E-05 | up | 2.58 | 15.67 |
|  | MNEHDJFA_04703 | ***phnP*** | 4.211 | 2.074 | 1.08E-03 | 2.16E-02 | up | 0.49 | 2.45 |
|  | MNEHDJFA_04704 | ***cat*** | 6.876 | 2.782 | 4.29E-05 | 2.21E-03 | up | 0.72 | 4.65 |
|  | MNEHDJFA_04773 | MNEHDJFA_04773 | 3.227 | 1.690 | 5.69E-04 | 1.41E-02 | up | 2.08 | 7.67 |
|  | MNEHDJFA_04795 | *gap2* | 9.299 | 3.217 | 1.09E-10 | 1.77E-07 | up | 1.51 | 14.35 |
|  | MNEHDJFA_04833 | *msrAB* | 0.302 | -1.728 | 1.04E-03 | 2.12E-02 | down | 4.70 | 2.25 |
|  | MNEHDJFA_04869 | MNEHDJFA_04869 | 4.141 | 2.050 | 1.96E-03 | 3.32E-02 | up | 1.40 | 6.43 |
|  | MNEHDJFA_04885 | MNEHDJFA_04885 | 5.661 | 2.501 | 3.59E-04 | 9.64E-03 | up | 105.43 | 512.03 |
|  | MNEHDJFA_04893 | *tpx* | 5.831 | 2.544 | 2.26E-04 | 6.98E-03 | up | 8.43 | 40.29 |
|  | MNEHDJFA_04900 | *tmpC* | 0.351 | -1.511 | 2.16E-04 | 6.79E-03 | down | 12.83 | 6.80 |
|  | MNEHDJFA_04915 | *yccF* | 3.610 | 1.852 | 5.28E-04 | 1.34E-02 | up | 2.84 | 10.53 |
|  | MNEHDJFA_04954 | *fruA* | 0.281 | -1.829 | 2.13E-04 | 6.79E-03 | down | 5.83 | 2.55 |
|  | MNEHDJFA_04955 | *lacC* | 0.298 | -1.748 | 7.35E-04 | 1.68E-02 | down | 8.23 | 3.19 |
|  | MNEHDJFA_04998 | *degA* | 2.508 | 1.326 | 2.42E-03 | 3.85E-02 | up | 1.27 | 3.68 |
|  | MNEHDJFA_05007 | *atpD* | 0.220 | -2.185 | 1.23E-03 | 2.36E-02 | down | 115.53 | 36.44 |
|  | MNEHDJFA_05008 | *atpG* | 0.194 | -2.363 | 9.37E-04 | 1.97E-02 | down | 180.50 | 52.76 |
|  | MNEHDJFA_05009 | *atpA* | 0.182 | -2.457 | 8.45E-04 | 1.84E-02 | down | 144.91 | 41.11 |
|  | MNEHDJFA_05010 | *atpH* | 0.180 | -2.470 | 2.25E-04 | 6.98E-03 | down | 26.14 | 7.59 |
|  | MNEHDJFA_05011 | *atpF* | 0.202 | -2.310 | 2.52E-03 | 3.96E-02 | down | 72.50 | 23.83 |
|  | MNEHDJFA_05012 | *atpE* | 0.075 | -3.741 | 1.96E-07 | 4.65E-05 | down | 25.12 | 2.55 |
|  | MNEHDJFA_05013 | *atpB* | 0.123 | -3.027 | 4.02E-05 | 2.16E-03 | down | 26.66 | 4.66 |
|  | MNEHDJFA_05014 | MNEHDJFA_05014 | 0.204 | -2.296 | 9.02E-04 | 1.92E-02 | down | 27.36 | 6.97 |
| WXL032 | COKMCCJN_00011 | COKMCCJN_00011 | 0.179 | -2.484 | 6.97E-10 | 3.81E-08 | down | 58.75 | 6.55 |
|  | COKMCCJN_00017 | *fadB* | 0.392 | -1.350 | 5.45E-04 | 4.03E-03 | down | 19.71 | 4.51 |
|  | COKMCCJN_00029 | *mco* | 0.340 | -1.555 | 1.41E-06 | 2.94E-05 | down | 4.59 | 0.95 |
|  | COKMCCJN_00037 | COKMCCJN_00037 | 0.499 | -1.004 | 5.24E-04 | 3.91E-03 | down | 8.37 | 2.53 |
|  | COKMCCJN_00055 | *rhlB* | 0.395 | -1.341 | 1.35E-06 | 2.85E-05 | down | 69.71 | 17.23 |
|  | COKMCCJN_00074 | *hemC* | 0.396 | -1.337 | 7.41E-06 | 1.20E-04 | down | 29.85 | 7.55 |
|  | COKMCCJN_00078 | *dapF* | 0.496 | -1.012 | 1.24E-03 | 7.99E-03 | down | 13.81 | 4.11 |
|  | COKMCCJN_00100 | COKMCCJN_00100 | 2.068 | 1.048 | 3.96E-03 | 1.95E-02 | up | 3.54 | 4.72 |
|  | COKMCCJN_00106 | *rpoH* | 2.329 | 1.219 | 4.06E-04 | 3.18E-03 | up | 132.23 | 210.38 |
|  | COKMCCJN_00107 | *glpE* | 3.949 | 1.981 | 6.98E-06 | 1.14E-04 | up | 31.09 | 87.67 |
|  | COKMCCJN_00108 | *glpG* | 2.080 | 1.057 | 4.47E-03 | 2.13E-02 | up | 11.14 | 15.77 |
|  | COKMCCJN_00115 | COKMCCJN_00115 | 0.319 | -1.647 | 1.52E-03 | 9.29E-03 | down | 8.33 | 1.46 |
|  | COKMCCJN_00118 | *fabR* | 2.553 | 1.352 | 4.40E-05 | 5.37E-04 | up | 79.57 | 133.71 |
|  | COKMCCJN_00119 | *yijD* | 2.542 | 1.346 | 1.31E-05 | 1.95E-04 | up | 12.55 | 20.37 |
|  | COKMCCJN_00121 | *btuB* | 2.906 | 1.539 | 4.88E-06 | 8.39E-05 | up | 6.84 | 12.25 |
|  | COKMCCJN_00124 | COKMCCJN_00124 | 2.346 | 1.230 | 6.13E-03 | 2.74E-02 | up | 2.17 | 3.16 |
|  | COKMCCJN_00141 | COKMCCJN_00141 | 0.431 | -1.215 | 9.04E-03 | 3.76E-02 | down | 4.55 | 1.16 |
|  | COKMCCJN_00150 | *secE* | 2.616 | 1.387 | 1.52E-03 | 9.29E-03 | up | 14.08 | 24.81 |
|  | COKMCCJN_00153 | *rplA* | 2.315 | 1.211 | 4.03E-04 | 3.17E-03 | up | 27.16 | 38.28 |
|  | COKMCCJN_00154 | *rplJ* | 2.890 | 1.531 | 6.10E-06 | 1.01E-04 | up | 63.58 | 113.19 |
|  | COKMCCJN_00155 | *rplL* | 4.280 | 2.098 | 3.67E-10 | 2.18E-08 | up | 18.23 | 49.57 |
|  | COKMCCJN_00158 | *rsd* | 0.316 | -1.662 | 1.81E-08 | 6.50E-07 | down | 146.57 | 30.07 |
|  | COKMCCJN_00161 | COKMCCJN_00161 | 0.484 | -1.048 | 6.29E-03 | 2.80E-02 | down | 8.39 | 2.46 |
|  | COKMCCJN_00162 | *ghrA* | 0.398 | -1.329 | 1.40E-04 | 1.36E-03 | down | 7.48 | 1.88 |
|  | COKMCCJN_00169 | *zntR* | 2.424 | 1.277 | 4.52E-05 | 5.50E-04 | up | 21.82 | 34.93 |
|  | COKMCCJN_00170 | *fis* | 3.028 | 1.598 | 4.80E-05 | 5.75E-04 | up | 5.97 | 12.01 |
|  | COKMCCJN_00176 | *acs* | 5.866 | 2.552 | 2.02E-12 | 2.05E-10 | up | 6.30 | 22.27 |
|  | COKMCCJN_00178 | COKMCCJN_00178 | 2.475 | 1.308 | 1.65E-04 | 1.55E-03 | up | 2.17 | 3.23 |
|  | COKMCCJN_00181 | *fumC* | 0.237 | -2.076 | 8.64E-04 | 6.00E-03 | down | 22.19 | 2.64 |
|  | COKMCCJN_00183 | *actP* | 40.478 | 5.339 | 4.69E-32 | 1.95E-28 | up | 0.56 | 15.13 |
|  | COKMCCJN_00184 | COKMCCJN_00184 | 10.076 | 3.333 | 2.60E-03 | 1.41E-02 | up | 0.33 | 1.92 |
|  | COKMCCJN_00187 | *dsbD* | 3.482 | 1.800 | 1.54E-07 | 4.45E-06 | up | 10.84 | 25.26 |
|  | COKMCCJN_00194 | COKMCCJN_00194 | 2.868 | 1.520 | 2.45E-03 | 1.36E-02 | up | 0.25 | 0.47 |
|  | COKMCCJN_00195 | *dcuA* | 2.066 | 1.047 | 9.39E-04 | 6.39E-03 | up | 4.01 | 5.22 |
|  | COKMCCJN_00196 | *aspA* | 2.270 | 1.182 | 8.94E-03 | 3.73E-02 | up | 48.57 | 65.97 |
|  | COKMCCJN_00213 | *rraA* | 2.124 | 1.087 | 1.78E-04 | 1.65E-03 | up | 30.14 | 39.43 |
|  | COKMCCJN_00220 | *rpmE* | 2.200 | 1.137 | 2.06E-04 | 1.85E-03 | up | 40.40 | 57.49 |
|  | COKMCCJN_00222 | *metJ* | 3.106 | 1.635 | 5.53E-05 | 6.42E-04 | up | 30.94 | 65.40 |
|  | COKMCCJN_00224 | *metL* | 2.475 | 1.307 | 1.11E-05 | 1.69E-04 | up | 10.08 | 15.97 |
|  | COKMCCJN_00225 | *metF* | 2.573 | 1.364 | 4.01E-04 | 3.17E-03 | up | 6.65 | 11.79 |
|  | COKMCCJN_00227 | *ppc* | 0.451 | -1.148 | 5.31E-03 | 2.45E-02 | down | 30.98 | 9.87 |
|  | COKMCCJN_00233 | *yabJ* | 3.206 | 1.681 | 9.96E-08 | 3.00E-06 | up | 17.04 | 36.14 |
|  | COKMCCJN_00234 | *pdhD* | 2.560 | 1.356 | 4.75E-06 | 8.36E-05 | up | 20.16 | 33.86 |
|  | COKMCCJN_00235 | *PGdx* | 2.923 | 1.547 | 3.52E-06 | 6.54E-05 | up | 82.54 | 156.82 |
|  | COKMCCJN_00236 | *oxyR* | 0.320 | -1.645 | 3.12E-04 | 2.61E-03 | down | 61.67 | 13.64 |
|  | COKMCCJN_00246 | *dam* | 0.466 | -1.102 | 4.03E-03 | 1.97E-02 | down | 6.77 | 1.85 |
|  | COKMCCJN_00252 | *aruC* | 0.415 | -1.270 | 1.13E-03 | 7.46E-03 | down | 11.39 | 2.87 |
|  | COKMCCJN_00256 | *crp* | 2.510 | 1.327 | 3.13E-06 | 5.91E-05 | up | 52.01 | 79.60 |
|  | COKMCCJN_00274 | *rpsL* | 4.205 | 2.072 | 3.76E-10 | 2.21E-08 | up | 75.81 | 189.13 |
|  | COKMCCJN_00275 | *rpsG* | 5.190 | 2.376 | 1.77E-11 | 1.39E-09 | up | 26.24 | 82.46 |
|  | COKMCCJN_00278 | *rpsJ* | 4.993 | 2.320 | 2.28E-15 | 4.51E-13 | up | 199.71 | 639.28 |
|  | COKMCCJN_00279 | *rplC* | 6.017 | 2.589 | 8.31E-15 | 1.48E-12 | up | 136.72 | 508.37 |
|  | COKMCCJN_00280 | *rplD* | 8.936 | 3.160 | 6.35E-20 | 2.20E-17 | up | 30.47 | 165.25 |
|  | COKMCCJN_00281 | *rplW* | 5.055 | 2.338 | 1.15E-12 | 1.23E-10 | up | 156.22 | 474.82 |
|  | COKMCCJN_00282 | *rplB* | 7.748 | 2.954 | 1.84E-27 | 3.82E-24 | up | 41.22 | 196.95 |
|  | COKMCCJN_00283 | *rpsS* | 7.215 | 2.851 | 2.16E-19 | 6.92E-17 | up | 28.24 | 125.35 |
|  | COKMCCJN_00284 | *rplV* | 6.664 | 2.736 | 4.01E-15 | 7.58E-13 | up | 255.51 | 1027.02 |
|  | COKMCCJN_00285 | *rpsC* | 8.050 | 3.009 | 1.52E-23 | 1.06E-20 | up | 52.93 | 257.79 |
|  | COKMCCJN_00286 | *rplP* | 7.004 | 2.808 | 2.03E-16 | 4.69E-14 | up | 299.81 | 1282.77 |
|  | COKMCCJN_00287 | *rpmC* | 6.493 | 2.699 | 1.14E-09 | 5.91E-08 | up | 1440.52 | 5854.63 |
|  | COKMCCJN_00288 | *rpsQ* | 5.285 | 2.402 | 1.31E-11 | 1.11E-09 | up | 135.20 | 420.56 |
|  | COKMCCJN_00290 | *rplX* | 2.433 | 1.283 | 8.76E-04 | 6.06E-03 | up | 58.00 | 86.32 |
|  | COKMCCJN_00291 | *rplE* | 2.300 | 1.202 | 3.95E-04 | 3.14E-03 | up | 48.56 | 68.08 |
|  | COKMCCJN_00292 | *rpsN* | 2.242 | 1.165 | 4.95E-03 | 2.33E-02 | up | 25.92 | 36.19 |
|  | COKMCCJN_00293 | *rpsH* | 2.820 | 1.496 | 3.27E-04 | 2.70E-03 | up | 87.51 | 146.77 |
|  | COKMCCJN_00294 | *rplF* | 2.877 | 1.524 | 1.61E-04 | 1.52E-03 | up | 55.90 | 94.96 |
|  | COKMCCJN_00295 | *rplR* | 3.208 | 1.682 | 1.80E-05 | 2.54E-04 | up | 66.33 | 129.86 |
|  | COKMCCJN_00296 | *rpsE* | 5.006 | 2.324 | 6.43E-11 | 4.69E-09 | up | 30.50 | 93.89 |
|  | COKMCCJN_00297 | *rpmD* | 5.723 | 2.517 | 8.81E-11 | 5.91E-09 | up | 17.67 | 66.18 |
|  | COKMCCJN_00298 | *rplO* | 4.457 | 2.156 | 2.25E-10 | 1.38E-08 | up | 78.88 | 211.68 |
|  | COKMCCJN_00299 | *secY* | 5.006 | 2.324 | 1.40E-16 | 3.42E-14 | up | 92.87 | 292.71 |
|  | COKMCCJN_00300 | *rpmJ* | 10.904 | 3.447 | 9.80E-22 | 5.10E-19 | up | 20.61 | 148.46 |
|  | COKMCCJN_00301 | *rpsM* | 4.360 | 2.124 | 1.07E-08 | 4.27E-07 | up | 379.21 | 1024.01 |
|  | COKMCCJN_00302 | *rpsK* | 3.488 | 1.803 | 4.20E-07 | 1.04E-05 | up | 65.18 | 138.52 |
|  | COKMCCJN_00303 | *rpsD* | 3.161 | 1.660 | 4.47E-06 | 7.97E-05 | up | 988.72 | 1880.15 |
|  | COKMCCJN_00304 | *rpoA* | 4.023 | 2.008 | 4.82E-10 | 2.75E-08 | up | 97.78 | 239.44 |
|  | COKMCCJN_00305 | *rplQ* | 4.147 | 2.052 | 1.18E-06 | 2.55E-05 | up | 103.30 | 258.33 |
|  | COKMCCJN_00312 | COKMCCJN_00312 | 0.359 | -1.477 | 2.23E-05 | 3.08E-04 | down | 96.84 | 22.31 |
|  | COKMCCJN_00318 | COKMCCJN_00318 | 5.390 | 2.430 | 7.28E-07 | 1.66E-05 | up | 4.53 | 18.25 |
|  | COKMCCJN_00319 | *cpdB* | 0.385 | -1.379 | 1.55E-04 | 1.49E-03 | down | 58.79 | 14.75 |
|  | COKMCCJN_00330 | *ytfJ* | 2.227 | 1.155 | 5.33E-05 | 6.23E-04 | up | 17.51 | 23.96 |
|  | COKMCCJN_00331 | COKMCCJN_00331 | 3.549 | 1.827 | 1.73E-03 | 1.02E-02 | up | 1.60 | 3.77 |
|  | COKMCCJN_00332 | *msrA* | 8.759 | 3.131 | 4.55E-06 | 8.09E-05 | up | 92.21 | 648.42 |
|  | COKMCCJN_00335 | *ytfP* | 14.455 | 3.853 | 2.47E-09 | 1.14E-07 | up | 81.02 | 851.70 |
|  | COKMCCJN_00336 | COKMCCJN_00336 | 6.893 | 2.785 | 1.66E-06 | 3.38E-05 | up | 85.40 | 426.36 |
|  | COKMCCJN_00342 | *thiQ* | 2.480 | 1.310 | 4.47E-03 | 2.13E-02 | up | 1.01 | 1.51 |
|  | COKMCCJN_00352 | *lptA* | 3.598 | 1.847 | 4.26E-05 | 5.23E-04 | up | 22.44 | 55.88 |
|  | COKMCCJN_00356 | *yrbG* | 2.501 | 1.323 | 1.27E-03 | 8.09E-03 | up | 9.78 | 16.85 |
|  | COKMCCJN_00365 | COKMCCJN_00365 | 0.463 | -1.112 | 1.44E-03 | 8.86E-03 | down | 179.38 | 52.15 |
|  | COKMCCJN_00368 | *argF* | 2.138 | 1.096 | 1.20E-03 | 7.77E-03 | up | 4.32 | 6.04 |
|  | COKMCCJN_00409 | *mltC* | 0.487 | -1.038 | 9.87E-04 | 6.66E-03 | down | 8.90 | 2.65 |
|  | COKMCCJN_00411 | *mutY* | 0.452 | -1.144 | 8.01E-04 | 5.64E-03 | down | 4.65 | 1.29 |
|  | COKMCCJN_00417 | COKMCCJN_00417 | 2.846 | 1.509 | 3.45E-04 | 2.81E-03 | up | 10.03 | 18.54 |
|  | COKMCCJN_00418 | COKMCCJN_00418 | 0.420 | -1.253 | 9.83E-05 | 1.04E-03 | down | 13.30 | 3.51 |
|  | COKMCCJN_00429 | *sprT* | 0.389 | -1.363 | 1.44E-03 | 8.86E-03 | down | 7.28 | 1.74 |
|  | COKMCCJN_00431 | *metK* | 2.137 | 1.095 | 1.15E-03 | 7.57E-03 | up | 8.44 | 12.14 |
|  | COKMCCJN_00433 | *epd* | 2.313 | 1.210 | 8.05E-05 | 8.76E-04 | up | 8.75 | 13.32 |
|  | COKMCCJN_00437 | *argO* | 3.351 | 1.744 | 1.18E-06 | 2.55E-05 | up | 2.58 | 5.57 |
|  | COKMCCJN_00451 | *nadB* | 0.379 | -1.399 | 8.85E-06 | 1.40E-04 | down | 320.26 | 78.68 |
|  | COKMCCJN_00452 | *rpoE* | 3.179 | 1.669 | 9.94E-06 | 1.54E-04 | up | 242.98 | 531.05 |
|  | COKMCCJN_00453 | *rseA* | 3.944 | 1.980 | 5.62E-10 | 3.16E-08 | up | 517.70 | 1366.54 |
|  | COKMCCJN_00454 | *rseB* | 3.357 | 1.747 | 1.09E-08 | 4.31E-07 | up | 135.03 | 293.30 |
|  | COKMCCJN_00455 | *rseC* | 2.601 | 1.379 | 9.47E-05 | 1.00E-03 | up | 26.55 | 45.44 |
|  | COKMCCJN_00471 | *ispF* | 0.481 | -1.056 | 3.51E-03 | 1.78E-02 | down | 7.95 | 2.40 |
|  | COKMCCJN_00475 | *nlpD* | 0.306 | -1.707 | 1.60E-06 | 3.28E-05 | down | 308.85 | 64.22 |
|  | COKMCCJN_00476 | *rpoS* | 0.483 | -1.049 | 2.29E-03 | 1.28E-02 | down | 135.60 | 43.16 |
|  | COKMCCJN_00495 | *gcdB* | 2.003 | 1.002 | 3.30E-03 | 1.71E-02 | up | 16.48 | 19.80 |
|  | COKMCCJN_00505 | *rpsP* | 4.296 | 2.103 | 2.27E-13 | 2.86E-11 | up | 31.12 | 83.54 |
|  | COKMCCJN_00506 | *rimM* | 3.795 | 1.924 | 6.06E-07 | 1.41E-05 | up | 59.66 | 130.93 |
|  | COKMCCJN_00507 | *trmD* | 5.315 | 2.410 | 4.92E-11 | 3.65E-09 | up | 29.98 | 94.10 |
|  | COKMCCJN_00508 | *rplS* | 4.988 | 2.318 | 9.67E-09 | 3.91E-07 | up | 15.45 | 46.28 |
|  | COKMCCJN_00510 | *zapD* | 0.481 | -1.056 | 3.06E-04 | 2.57E-03 | down | 19.64 | 6.07 |
|  | COKMCCJN_00511 | *coaE* | 0.295 | -1.760 | 1.56E-03 | 9.45E-03 | down | 2.79 | 0.50 |
|  | COKMCCJN_00533 | *dksA* | 2.918 | 1.545 | 1.68E-06 | 3.39E-05 | up | 13.82 | 24.89 |
|  | COKMCCJN_00539 | COKMCCJN_00539 | 4.643 | 2.215 | 1.77E-08 | 6.40E-07 | up | 17.70 | 57.12 |
|  | COKMCCJN_00542 | *futA1* | 0.397 | -1.332 | 3.39E-03 | 1.74E-02 | down | 68.15 | 15.44 |
|  | COKMCCJN_00544 | *potA* | 0.324 | -1.625 | 5.89E-05 | 6.76E-04 | down | 11.57 | 2.12 |
|  | COKMCCJN_00556 | *rsmC* | 0.494 | -1.018 | 6.29E-03 | 2.80E-02 | down | 5.02 | 1.51 |
|  | COKMCCJN_00566 | *dacB* | 0.350 | -1.513 | 1.03E-06 | 2.25E-05 | down | 6.08 | 1.32 |
|  | COKMCCJN_00567 | *ompU* | 0.228 | -2.130 | 5.11E-05 | 6.04E-04 | down | 182.86 | 22.16 |
|  | COKMCCJN_00581 | *truB* | 2.375 | 1.248 | 5.91E-04 | 4.32E-03 | up | 3.00 | 4.40 |
|  | COKMCCJN_00582 | *rpsO* | 5.285 | 2.402 | 1.41E-11 | 1.16E-09 | up | 467.57 | 1524.18 |
|  | COKMCCJN_00587 | COKMCCJN_00587 | 3.638 | 1.863 | 2.78E-04 | 2.38E-03 | up | 0.64 | 1.54 |
|  | COKMCCJN_00589 | COKMCCJN_00589 | 0.408 | -1.292 | 6.10E-05 | 6.95E-04 | down | 6.07 | 1.50 |
|  | COKMCCJN_00590 | *ubiJ* | 2.262 | 1.178 | 1.27E-02 | 4.92E-02 | up | 1.74 | 2.68 |
|  | COKMCCJN_00591 | COKMCCJN_00591 | 2.209 | 1.143 | 6.01E-03 | 2.71E-02 | up | 2.37 | 3.42 |
|  | COKMCCJN_00597 | *nupX* | 2.139 | 1.097 | 6.38E-05 | 7.17E-04 | up | 15.67 | 20.45 |
|  | COKMCCJN_00616 | COKMCCJN_00616 | 2.180 | 1.124 | 1.31E-03 | 8.26E-03 | up | 2.82 | 3.67 |
|  | COKMCCJN_00618 | COKMCCJN_00618 | 7.903 | 2.982 | 1.59E-05 | 2.30E-04 | up | 1.80 | 9.24 |
|  | COKMCCJN_00619 | COKMCCJN_00619 | 7.913 | 2.984 | 7.91E-05 | 8.64E-04 | up | 0.19 | 0.93 |
|  | COKMCCJN_00620 | COKMCCJN_00620 | 4.494 | 2.168 | 6.28E-04 | 4.56E-03 | up | 0.14 | 0.40 |
|  | COKMCCJN_00646 | *melR* | 0.481 | -1.056 | 1.74E-03 | 1.03E-02 | down | 19.77 | 6.17 |
|  | COKMCCJN_00660 | *pepD* | 0.334 | -1.583 | 1.53E-08 | 5.64E-07 | down | 50.03 | 10.18 |
|  | COKMCCJN_00663 | COKMCCJN_00663 | 0.421 | -1.248 | 1.57E-04 | 1.49E-03 | down | 48.80 | 13.79 |
|  | COKMCCJN_00667 | *nrdR* | 0.365 | -1.455 | 1.16E-04 | 1.19E-03 | down | 9.32 | 2.19 |
|  | COKMCCJN_00679 | *pomA* | 2.674 | 1.419 | 2.28E-05 | 3.15E-04 | up | 3.98 | 6.88 |
|  | COKMCCJN_00680 | *motB* | 2.355 | 1.236 | 1.26E-03 | 8.07E-03 | up | 7.69 | 11.32 |
|  | COKMCCJN_00686 | *ygdG* | 0.344 | -1.538 | 4.30E-04 | 3.34E-03 | down | 6.62 | 1.53 |
|  | COKMCCJN_00687 | *ppnN* | 0.332 | -1.591 | 3.25E-05 | 4.20E-04 | down | 28.57 | 5.53 |
|  | COKMCCJN_00688 | COKMCCJN_00688 | 0.449 | -1.155 | 6.29E-05 | 7.11E-04 | down | 9.46 | 2.58 |
|  | COKMCCJN_00689 | COKMCCJN_00689 | 0.441 | -1.182 | 5.21E-04 | 3.90E-03 | down | 5.98 | 1.58 |
|  | COKMCCJN_00690 | *queF* | 0.379 | -1.398 | 5.77E-06 | 9.67E-05 | down | 11.82 | 2.82 |
|  | COKMCCJN_00691 | *syd* | 0.425 | -1.233 | 6.06E-04 | 4.41E-03 | down | 9.21 | 2.57 |
|  | COKMCCJN_00692 | COKMCCJN_00692 | 0.489 | -1.032 | 1.57E-03 | 9.51E-03 | down | 9.31 | 2.95 |
|  | COKMCCJN_00694 | *metQ* | 3.234 | 1.693 | 2.78E-07 | 7.33E-06 | up | 39.28 | 77.52 |
|  | COKMCCJN_00699 | *treR* | 0.383 | -1.383 | 2.00E-05 | 2.81E-04 | down | 8.64 | 2.00 |
|  | COKMCCJN_00712 | *abgT* | 3.360 | 1.749 | 8.21E-06 | 1.32E-04 | up | 1.40 | 2.95 |
|  | COKMCCJN_00713 | COKMCCJN_00713 | 4.213 | 2.075 | 2.64E-03 | 1.42E-02 | up | 8.50 | 29.19 |
|  | COKMCCJN_00722 | *glnD* | 0.316 | -1.663 | 5.20E-08 | 1.72E-06 | down | 16.41 | 3.19 |
|  | COKMCCJN_00724 | *rpsB* | 2.442 | 1.288 | 3.95E-05 | 4.90E-04 | up | 41.98 | 65.69 |
|  | COKMCCJN_00731 | *rseP* | 2.249 | 1.169 | 3.40E-04 | 2.78E-03 | up | 74.64 | 111.21 |
|  | COKMCCJN_00732 | *bamA* | 2.025 | 1.018 | 7.90E-04 | 5.57E-03 | up | 49.77 | 66.43 |
|  | COKMCCJN_00742 | COKMCCJN_00742 | 2.037 | 1.027 | 2.51E-03 | 1.38E-02 | up | 23.43 | 30.31 |
|  | COKMCCJN_00744 | COKMCCJN_00744 | 0.489 | -1.033 | 1.02E-03 | 6.81E-03 | down | 5.30 | 1.59 |
|  | COKMCCJN_00757 | *purN* | 0.333 | -1.585 | 3.88E-05 | 4.84E-04 | down | 12.78 | 2.45 |
|  | COKMCCJN_00758 | *purM* | 2.855 | 1.514 | 1.45E-06 | 3.00E-05 | up | 3.53 | 6.49 |
|  | COKMCCJN_00760 | *pyrP* | 3.046 | 1.607 | 1.02E-02 | 4.13E-02 | up | 0.22 | 0.39 |
|  | COKMCCJN_00762 | COKMCCJN_00762 | 2.140 | 1.097 | 1.18E-03 | 7.71E-03 | up | 8.25 | 11.12 |
|  | COKMCCJN_00765 | *bepA* | 2.777 | 1.473 | 1.61E-04 | 1.52E-03 | up | 106.58 | 198.38 |
|  | COKMCCJN_00770 | *dapA* | 4.706 | 2.235 | 8.93E-09 | 3.68E-07 | up | 21.97 | 69.76 |
|  | COKMCCJN_00771 | *bamC* | 2.652 | 1.407 | 1.63E-05 | 2.33E-04 | up | 40.61 | 71.46 |
|  | COKMCCJN_00775 | *yffB* | 0.426 | -1.232 | 4.47E-03 | 2.13E-02 | down | 9.55 | 2.59 |
|  | COKMCCJN_00780 | *flaD* | 4.302 | 2.105 | 1.59E-08 | 5.80E-07 | up | 7.31 | 20.16 |
|  | COKMCCJN_00781 | *flaD* | 3.003 | 1.586 | 1.06E-04 | 1.11E-03 | up | 16.96 | 30.37 |
|  | COKMCCJN_00787 | *zipA* | 2.154 | 1.107 | 1.38E-03 | 8.60E-03 | up | 38.07 | 54.45 |
|  | COKMCCJN_00812 | *asnB* | 0.209 | -2.256 | 9.84E-04 | 6.66E-03 | down | 30.60 | 5.04 |
|  | COKMCCJN_00826 | COKMCCJN_00826 | 0.453 | -1.142 | 1.53E-03 | 9.30E-03 | down | 6.61 | 1.99 |
|  | COKMCCJN_00829 | *sdhD* | 0.356 | -1.491 | 1.71E-04 | 1.60E-03 | down | 25.51 | 5.37 |
|  | COKMCCJN_00837 | *znuC* | 0.377 | -1.408 | 1.16E-04 | 1.19E-03 | down | 19.24 | 4.92 |
|  | COKMCCJN_00838 | *znuA* | 0.247 | -2.017 | 4.64E-08 | 1.56E-06 | down | 9.82 | 1.55 |
|  | COKMCCJN_00840 | COKMCCJN_00840 | 0.500 | -1.001 | 7.68E-03 | 3.30E-02 | down | 60.70 | 18.37 |
|  | COKMCCJN_00851 | COKMCCJN_00851 | 2.808 | 1.489 | 3.91E-04 | 3.13E-03 | up | 129.31 | 255.60 |
|  | COKMCCJN_00852 | *menH* | 2.186 | 1.128 | 1.64E-03 | 9.82E-03 | up | 2.97 | 4.10 |
|  | COKMCCJN_00856 | *minD* | 0.410 | -1.286 | 3.52E-05 | 4.47E-04 | down | 35.61 | 8.79 |
|  | COKMCCJN_00859 | COKMCCJN_00859 | 0.480 | -1.058 | 3.04E-04 | 2.56E-03 | down | 40.55 | 12.32 |
|  | COKMCCJN_00865 | *aroC* | 0.481 | -1.057 | 1.88E-04 | 1.72E-03 | down | 31.12 | 9.65 |
|  | COKMCCJN_00878 | *dedD* | 0.415 | -1.270 | 4.42E-04 | 3.42E-03 | down | 9.44 | 2.40 |
|  | COKMCCJN_00886 | COKMCCJN_00886 | 4.888 | 2.289 | 3.93E-11 | 2.97E-09 | up | 39.98 | 133.60 |
|  | COKMCCJN_00887 | *ybaB* | 2.968 | 1.569 | 5.16E-04 | 3.88E-03 | up | 267.80 | 587.72 |
|  | COKMCCJN_00888 | *recR* | 2.339 | 1.226 | 3.79E-04 | 3.05E-03 | up | 150.90 | 241.64 |
|  | COKMCCJN_00893 | *caiT* | 2.536 | 1.342 | 4.31E-03 | 2.08E-02 | up | 0.32 | 0.48 |
|  | COKMCCJN_00894 | COKMCCJN_00894 | 3.896 | 1.962 | 1.69E-04 | 1.58E-03 | up | 38.21 | 91.14 |
|  | COKMCCJN_00897 | COKMCCJN_00897 | 2.196 | 1.135 | 1.76E-03 | 1.04E-02 | up | 1.25 | 1.66 |
|  | COKMCCJN_00917 | COKMCCJN_00917 | 0.482 | -1.054 | 1.28E-03 | 8.16E-03 | down | 5.19 | 1.51 |
|  | COKMCCJN_00923 | *adhE* | 4.654 | 2.218 | 1.26E-07 | 3.68E-06 | up | 100.31 | 305.82 |
|  | COKMCCJN_00931 | *pal* | 2.896 | 1.534 | 4.76E-06 | 8.36E-05 | up | 2.50 | 4.69 |
|  | COKMCCJN_00933 | *gloA* | 0.404 | -1.308 | 1.29E-05 | 1.93E-04 | down | 47.33 | 11.77 |
|  | COKMCCJN_00958 | *ackA* | 2.465 | 1.301 | 2.58E-05 | 3.47E-04 | up | 20.57 | 32.71 |
|  | COKMCCJN_00961 | COKMCCJN_00961 | 0.478 | -1.066 | 7.68E-04 | 5.44E-03 | down | 9.67 | 2.94 |
|  | COKMCCJN_00964 | *dsbB* | 2.532 | 1.340 | 2.61E-03 | 1.41E-02 | up | 13.69 | 22.51 |
|  | COKMCCJN_00966 | *fadR* | 0.443 | -1.175 | 1.04E-04 | 1.10E-03 | down | 42.90 | 12.05 |
|  | COKMCCJN_00975 | *rne* | 2.026 | 1.019 | 4.43E-03 | 2.12E-02 | up | 23.24 | 27.18 |
|  | COKMCCJN_00979 | *yceD* | 3.668 | 1.875 | 4.60E-07 | 1.12E-05 | up | 17.06 | 36.71 |
|  | COKMCCJN_00980 | *rpmF* | 5.350 | 2.419 | 1.55E-04 | 1.49E-03 | up | 5.40 | 19.17 |
|  | COKMCCJN_00998 | *mlc* | 0.394 | -1.345 | 1.41E-04 | 1.37E-03 | down | 7.09 | 1.68 |
|  | COKMCCJN_00999 | *cheV* | 0.475 | -1.074 | 1.70E-03 | 1.01E-02 | down | 8.33 | 2.55 |
|  | COKMCCJN_01001 | COKMCCJN_01001 | 2.703 | 1.434 | 5.88E-04 | 4.30E-03 | up | 1.95 | 3.70 |
|  | COKMCCJN_01002 | COKMCCJN_01002 | 2.839 | 1.505 | 2.44E-05 | 3.32E-04 | up | 4.21 | 7.62 |
|  | COKMCCJN_01010 | *pflA* | 0.453 | -1.142 | 2.08E-03 | 1.18E-02 | down | 43.41 | 13.20 |
|  | COKMCCJN_01012 | *pflB* | 5.860 | 2.551 | 5.78E-17 | 1.50E-14 | up | 247.30 | 944.62 |
|  | COKMCCJN_01013 | COKMCCJN_01013 | 9.353 | 3.225 | 3.90E-22 | 2.32E-19 | up | 1.29 | 7.69 |
|  | COKMCCJN_01014 | COKMCCJN_01014 | 2.431 | 1.282 | 1.06E-05 | 1.63E-04 | up | 4.88 | 7.65 |
|  | COKMCCJN_01028 | COKMCCJN_01028 | 6.972 | 2.801 | 6.41E-24 | 6.67E-21 | up | 4.77 | 21.20 |
|  | COKMCCJN_01037 | COKMCCJN_01037 | 2.046 | 1.033 | 7.30E-03 | 3.17E-02 | up | 87.11 | 122.94 |
|  | COKMCCJN_01041 | *glgC* | 0.452 | -1.145 | 2.63E-04 | 2.28E-03 | down | 87.06 | 23.41 |
|  | COKMCCJN_01044 | COKMCCJN_01044 | 5.789 | 2.533 | 5.27E-05 | 6.18E-04 | up | 16.26 | 76.60 |
|  | COKMCCJN_01045 | COKMCCJN_01045 | 5.470 | 2.452 | 2.68E-04 | 2.32E-03 | up | 103.99 | 473.53 |
|  | COKMCCJN_01049 | *torR* | 4.213 | 2.075 | 6.60E-10 | 3.66E-08 | up | 17.20 | 48.33 |
|  | COKMCCJN_01059 | *cmoB* | 0.372 | -1.425 | 5.23E-04 | 3.91E-03 | down | 2.36 | 0.53 |
|  | COKMCCJN_01061 | COKMCCJN_01061 | 0.489 | -1.032 | 4.20E-03 | 2.04E-02 | down | 8.04 | 2.63 |
|  | COKMCCJN_01075 | *pal_2* | 0.451 | -1.150 | 1.87E-03 | 1.09E-02 | down | 98.98 | 26.42 |
|  | COKMCCJN_01076 | *cpoB* | 0.398 | -1.328 | 3.37E-05 | 4.34E-04 | down | 35.28 | 8.32 |
|  | COKMCCJN_01077 | *nadA* | 0.418 | -1.258 | 6.26E-05 | 7.10E-04 | down | 21.91 | 6.01 |
|  | COKMCCJN_01093 | *csqR* | 0.429 | -1.222 | 9.86E-04 | 6.66E-03 | down | 6.83 | 1.96 |
|  | COKMCCJN_01095 | COKMCCJN_01095 | 0.466 | -1.102 | 2.42E-04 | 2.12E-03 | down | 55.41 | 16.89 |
|  | COKMCCJN_01097 | *siaT* | 0.391 | -1.353 | 3.61E-04 | 2.91E-03 | down | 2.91 | 0.71 |
|  | COKMCCJN_01104 | *czcR* | 2.655 | 1.409 | 7.61E-04 | 5.42E-03 | up | 29.62 | 46.14 |
|  | COKMCCJN_01109 | COKMCCJN_01109 | 2.334 | 1.223 | 2.30E-03 | 1.28E-02 | up | 4.29 | 5.81 |
|  | COKMCCJN_01112 | COKMCCJN_01112 | 3.021 | 1.595 | 5.33E-06 | 9.09E-05 | up | 20.82 | 40.84 |
|  | COKMCCJN_01113 | *metR* | 5.186 | 2.375 | 5.54E-07 | 1.30E-05 | up | 0.40 | 1.26 |
|  | COKMCCJN_01114 | *metE* | 5.069 | 2.342 | 4.47E-08 | 1.53E-06 | up | 6.86 | 20.02 |
|  | COKMCCJN_01119 | COKMCCJN_01119 | 5.116 | 2.355 | 5.62E-04 | 4.14E-03 | up | 0.81 | 2.62 |
|  | COKMCCJN_01120 | *gcvA* | 0.381 | -1.394 | 1.96E-03 | 1.13E-02 | down | 1.75 | 0.42 |
|  | COKMCCJN_01121 | *hisC* | 0.396 | -1.338 | 1.25E-02 | 4.88E-02 | down | 1.30 | 0.35 |
|  | COKMCCJN_01139 | *yeiP* | 0.428 | -1.223 | 5.03E-05 | 5.98E-04 | down | 118.00 | 33.23 |
|  | COKMCCJN_01143 | *uvrC* | 0.404 | -1.307 | 3.12E-05 | 4.07E-04 | down | 9.35 | 2.31 |
|  | COKMCCJN_01152 | COKMCCJN_01152 | 0.489 | -1.033 | 1.15E-03 | 7.57E-03 | down | 367.99 | 111.76 |
|  | COKMCCJN_01155 | *dmsC* | 3.993 | 1.997 | 5.17E-05 | 6.09E-04 | up | 4.69 | 13.90 |
|  | COKMCCJN_01156 | *dmsB* | 9.202 | 3.202 | 2.00E-12 | 2.05E-10 | up | 3.31 | 21.07 |
|  | COKMCCJN_01157 | *dmsA* | 3.752 | 1.908 | 2.37E-06 | 4.61E-05 | up | 8.91 | 23.59 |
|  | COKMCCJN_01158 | *dmsD* | 4.942 | 2.305 | 1.62E-07 | 4.64E-06 | up | 2.48 | 8.59 |
|  | COKMCCJN_01159 | *napF* | 6.993 | 2.806 | 7.80E-11 | 5.41E-09 | up | 1.26 | 5.93 |
|  | COKMCCJN_01160 | COKMCCJN_01160 | 5.509 | 2.462 | 1.47E-06 | 3.03E-05 | up | 2.83 | 9.67 |
|  | COKMCCJN_01161 | *nrdB* | 2.875 | 1.523 | 2.14E-05 | 2.97E-04 | up | 8.84 | 15.15 |
|  | COKMCCJN_01162 | *nrdA* | 2.369 | 1.244 | 9.37E-05 | 9.97E-04 | up | 3.04 | 4.25 |
|  | COKMCCJN_01167 | *nrfA* | 4.249 | 2.087 | 4.05E-09 | 1.76E-07 | up | 2.54 | 6.87 |
|  | COKMCCJN_01168 | *nrfB* | 2.522 | 1.334 | 5.18E-04 | 3.88E-03 | up | 5.02 | 8.23 |
|  | COKMCCJN_01169 | *ydhX* | 3.777 | 1.917 | 8.10E-07 | 1.82E-05 | up | 2.67 | 6.45 |
|  | COKMCCJN_01170 | COKMCCJN_01170 | 3.636 | 1.862 | 6.21E-05 | 7.06E-04 | up | 1.04 | 2.29 |
|  | COKMCCJN_01184 | COKMCCJN_01184 | 5.088 | 2.347 | 1.33E-06 | 2.81E-05 | up | 1.32 | 4.03 |
|  | COKMCCJN_01185 | COKMCCJN_01185 | 18.631 | 4.220 | 2.70E-14 | 4.32E-12 | up | 0.47 | 5.26 |
|  | COKMCCJN_01186 | COKMCCJN_01186 | 7.782 | 2.960 | 1.98E-04 | 1.79E-03 | up | 0.29 | 1.23 |
|  | COKMCCJN_01187 | *dctM* | 2.520 | 1.333 | 9.09E-04 | 6.23E-03 | up | 1.93 | 2.93 |
|  | COKMCCJN_01190 | COKMCCJN_01190 | 2.316 | 1.212 | 1.24E-04 | 1.26E-03 | up | 3.15 | 4.44 |
|  | COKMCCJN_01192 | *pctB* | 2.714 | 1.440 | 8.31E-07 | 1.86E-05 | up | 3.53 | 6.14 |
|  | COKMCCJN_01194 | *cydD* | 0.401 | -1.317 | 5.06E-05 | 6.00E-04 | down | 6.04 | 1.47 |
|  | COKMCCJN_01206 | *maeA* | 0.438 | -1.192 | 5.41E-05 | 6.31E-04 | down | 17.65 | 4.69 |
|  | COKMCCJN_01210 | COKMCCJN_01210 | 2.261 | 1.177 | 4.70E-04 | 3.57E-03 | up | 14.99 | 22.71 |
|  | COKMCCJN_01219 | *hutH* | 2.616 | 1.387 | 1.41E-03 | 8.75E-03 | up | 2.07 | 3.19 |
|  | COKMCCJN_01223 | COKMCCJN_01223 | 0.436 | -1.198 | 2.55E-03 | 1.39E-02 | down | 4.54 | 1.31 |
|  | COKMCCJN_01228 | *rpmI* | 3.153 | 1.657 | 9.61E-06 | 1.50E-04 | up | 59.91 | 115.01 |
|  | COKMCCJN_01229 | *rplT* | 3.732 | 1.900 | 2.79E-04 | 2.39E-03 | up | 31.89 | 71.84 |
|  | COKMCCJN_01245 | COKMCCJN_01245 | 2.858 | 1.515 | 1.45E-08 | 5.48E-07 | up | 7.08 | 12.89 |
|  | COKMCCJN_01247 | *phnE* | 0.353 | -1.503 | 1.99E-04 | 1.79E-03 | down | 56.70 | 11.54 |
|  | COKMCCJN_01248 | *phnE* | 0.319 | -1.647 | 1.93E-04 | 1.75E-03 | down | 15.73 | 2.87 |
|  | COKMCCJN_01249 | *phnC* | 0.366 | -1.449 | 7.35E-04 | 5.27E-03 | down | 55.91 | 12.00 |
|  | COKMCCJN_01250 | *phnD* | 0.494 | -1.017 | 5.55E-03 | 2.54E-02 | down | 150.27 | 45.19 |
|  | COKMCCJN_01256 | COKMCCJN_01256 | 2.351 | 1.233 | 2.17E-03 | 1.22E-02 | up | 0.73 | 1.01 |
|  | COKMCCJN_01263 | *kdgM* | 0.413 | -1.276 | 3.13E-03 | 1.63E-02 | down | 8.00 | 1.84 |
|  | COKMCCJN_01264 | *alyA* | 2.218 | 1.149 | 3.58E-04 | 2.90E-03 | up | 4.46 | 6.50 |
|  | COKMCCJN_01272 | COKMCCJN_01272 | 0.284 | -1.816 | 7.82E-10 | 4.12E-08 | down | 6.61 | 1.18 |
|  | COKMCCJN_01273 | COKMCCJN_01273 | 0.334 | -1.583 | 1.19E-04 | 1.22E-03 | down | 4782.06 | 1129.45 |
|  | COKMCCJN_01276 | *msrAB* | 0.472 | -1.084 | 6.02E-04 | 4.39E-03 | down | 19.96 | 5.88 |
|  | COKMCCJN_01284 | *nprV* | 2.859 | 1.516 | 8.17E-04 | 5.73E-03 | up | 1.80 | 3.25 |
|  | COKMCCJN_01285 | COKMCCJN_01285 | 3.162 | 1.661 | 3.99E-03 | 1.95E-02 | up | 0.57 | 1.15 |
|  | COKMCCJN_01286 | COKMCCJN_01286 | 7.814 | 2.966 | 2.76E-03 | 1.48E-02 | up | 0.09 | 0.46 |
|  | COKMCCJN_01294 | COKMCCJN_01294 | 2.169 | 1.117 | 1.16E-02 | 4.62E-02 | up | 9.68 | 12.23 |
|  | COKMCCJN_01301 | COKMCCJN_01301 | 0.330 | -1.601 | 7.31E-04 | 5.25E-03 | down | 259.51 | 58.03 |
|  | COKMCCJN_01304 | *alsT* | 2.335 | 1.224 | 1.41E-03 | 8.75E-03 | up | 1.45 | 2.23 |
|  | COKMCCJN_01318 | COKMCCJN_01318 | 2.093 | 1.065 | 2.60E-03 | 1.41E-02 | up | 39.80 | 56.90 |
|  | COKMCCJN_01321 | COKMCCJN_01321 | 3.026 | 1.598 | 3.98E-04 | 3.15E-03 | up | 0.78 | 1.54 |
|  | COKMCCJN_01338 | *pal* | 0.435 | -1.200 | 8.51E-04 | 5.92E-03 | down | 5.38 | 1.45 |
|  | COKMCCJN_01340 | COKMCCJN_01340 | 0.471 | -1.085 | 1.57E-04 | 1.49E-03 | down | 16.94 | 5.09 |
|  | COKMCCJN_01343 | COKMCCJN_01343 | 2.750 | 1.460 | 2.94E-03 | 1.54E-02 | up | 3.94 | 6.75 |
|  | COKMCCJN_01344 | COKMCCJN_01344 | 5.336 | 2.416 | 9.89E-07 | 2.18E-05 | up | 21.66 | 76.10 |
|  | COKMCCJN_01345 | COKMCCJN_01345 | 2.476 | 1.308 | 4.29E-03 | 2.08E-02 | up | 24.14 | 38.62 |
|  | COKMCCJN_01346 | COKMCCJN_01346 | 2.644 | 1.403 | 5.44E-03 | 2.51E-02 | up | 0.87 | 1.44 |
|  | COKMCCJN_01347 | COKMCCJN_01347 | 7.036 | 2.815 | 9.57E-08 | 2.95E-06 | up | 3.46 | 16.77 |
|  | COKMCCJN_01348 | COKMCCJN_01348 | 5.282 | 2.401 | 2.01E-04 | 1.81E-03 | up | 1.10 | 3.94 |
|  | COKMCCJN_01350 | COKMCCJN_01350 | 2.177 | 1.122 | 3.18E-05 | 4.13E-04 | up | 546.27 | 744.55 |
|  | COKMCCJN_01351 | COKMCCJN_01351 | 3.534 | 1.821 | 2.67E-04 | 2.31E-03 | up | 3.29 | 7.62 |
|  | COKMCCJN_01352 | COKMCCJN_01352 | 3.658 | 1.871 | 4.05E-04 | 3.18E-03 | up | 10.21 | 22.21 |
|  | COKMCCJN_01354 | COKMCCJN_01354 | 8.718 | 3.124 | 4.85E-06 | 8.38E-05 | up | 1.00 | 5.37 |
|  | COKMCCJN_01356 | COKMCCJN_01356 | 3.086 | 1.626 | 1.36E-03 | 8.49E-03 | up | 2.14 | 4.22 |
|  | COKMCCJN_01359 | COKMCCJN_01359 | 6.418 | 2.682 | 1.20E-06 | 2.57E-05 | up | 22.49 | 95.60 |
|  | COKMCCJN_01361 | COKMCCJN_01361 | 3.486 | 1.802 | 1.92E-03 | 1.11E-02 | up | 0.67 | 1.49 |
|  | COKMCCJN_01362 | COKMCCJN_01362 | 3.254 | 1.702 | 5.62E-05 | 6.52E-04 | up | 4.02 | 8.15 |
|  | COKMCCJN_01363 | COKMCCJN_01363 | 2.398 | 1.262 | 9.29E-06 | 1.45E-04 | up | 678.88 | 1028.14 |
|  | COKMCCJN_01364 | COKMCCJN_01364 | 5.202 | 2.379 | 1.76E-04 | 1.64E-03 | up | 34.80 | 113.01 |
|  | COKMCCJN_01365 | COKMCCJN_01365 | 6.771 | 2.759 | 3.77E-05 | 4.76E-04 | up | 1.03 | 5.11 |
|  | COKMCCJN_01369 | COKMCCJN_01369 | 2.328 | 1.219 | 1.05E-02 | 4.25E-02 | up | 0.54 | 0.73 |
|  | COKMCCJN_01372 | *yccA* | 3.278 | 1.713 | 4.80E-06 | 8.36E-05 | up | 56.10 | 125.84 |
|  | COKMCCJN_01378 | COKMCCJN_01378 | 0.471 | -1.087 | 1.01E-03 | 6.80E-03 | down | 15.85 | 4.67 |
|  | COKMCCJN_01379 | *aroH* | 0.332 | -1.590 | 1.67E-09 | 8.09E-08 | down | 44.11 | 9.10 |
|  | COKMCCJN_01388 | *msrC* | 2.992 | 1.581 | 7.75E-04 | 5.48E-03 | up | 40.82 | 88.92 |
|  | COKMCCJN_01395 | *pyrD* | 3.128 | 1.645 | 3.32E-07 | 8.38E-06 | up | 9.79 | 18.21 |
|  | COKMCCJN_01399 | *uup* | 2.134 | 1.093 | 1.10E-03 | 7.33E-03 | up | 14.12 | 19.65 |
|  | COKMCCJN_01406 | COKMCCJN_01406 | 0.332 | -1.590 | 2.88E-03 | 1.53E-02 | down | 6.46 | 1.28 |
|  | COKMCCJN_01408 | COKMCCJN_01408 | 0.453 | -1.141 | 1.06E-02 | 4.28E-02 | down | 7.37 | 2.15 |
|  | COKMCCJN_01418 | COKMCCJN_01418 | 0.355 | -1.495 | 1.00E-02 | 4.08E-02 | down | 2.89 | 0.61 |
|  | COKMCCJN_01422 | *fnr* | 0.402 | -1.314 | 1.91E-03 | 1.11E-02 | down | 138.83 | 38.85 |
|  | COKMCCJN_01442 | COKMCCJN_01442 | 2.788 | 1.479 | 5.02E-08 | 1.67E-06 | up | 21.60 | 38.39 |
|  | COKMCCJN_01443 | COKMCCJN_01443 | 2.646 | 1.404 | 3.31E-07 | 8.38E-06 | up | 29.41 | 49.20 |
|  | COKMCCJN_01448 | *mcpP* | 2.377 | 1.249 | 2.94E-04 | 2.49E-03 | up | 5.65 | 8.02 |
|  | COKMCCJN_01452 | *tupB* | 2.145 | 1.101 | 4.51E-04 | 3.46E-03 | up | 5.54 | 7.09 |
|  | COKMCCJN_01460 | COKMCCJN_01460 | 0.469 | -1.093 | 2.79E-03 | 1.49E-02 | down | 11.99 | 3.28 |
|  | COKMCCJN_01473 | *ousW* | 2.897 | 1.535 | 4.14E-04 | 3.24E-03 | up | 1.21 | 2.06 |
|  | COKMCCJN_01474 | *opuAA* | 2.087 | 1.061 | 2.00E-03 | 1.14E-02 | up | 7.75 | 10.05 |
|  | COKMCCJN_01478 | COKMCCJN_01478 | 4.455 | 2.156 | 6.95E-09 | 2.89E-07 | up | 2.82 | 8.54 |
|  | COKMCCJN_01492 | *dmlR* | 3.676 | 1.878 | 3.03E-04 | 2.56E-03 | up | 0.32 | 0.71 |
|  | COKMCCJN_01494 | *ompD* | 3.802 | 1.927 | 1.12E-04 | 1.16E-03 | up | 0.42 | 1.06 |
|  | COKMCCJN_01505 | COKMCCJN_01505 | 0.255 | -1.973 | 6.40E-06 | 1.06E-04 | down | 9.76 | 1.62 |
|  | COKMCCJN_01517 | *artM* | 2.183 | 1.126 | 1.84E-03 | 1.08E-02 | up | 4.29 | 5.80 |
|  | COKMCCJN_01521 | *bmrA* | 0.306 | -1.709 | 2.77E-09 | 1.25E-07 | down | 8.90 | 1.71 |
|  | COKMCCJN_01524 | COKMCCJN_01524 | 0.388 | -1.367 | 1.16E-02 | 4.62E-02 | down | 1.45 | 0.35 |
|  | COKMCCJN_01528 | *cat* | 0.477 | -1.068 | 4.14E-03 | 2.01E-02 | down | 4.00 | 1.19 |
|  | COKMCCJN_01544 | COKMCCJN_01544 | 3.105 | 1.635 | 4.29E-07 | 1.06E-05 | up | 5.38 | 10.45 |
|  | COKMCCJN_01545 | COKMCCJN_01545 | 0.386 | -1.373 | 8.33E-03 | 3.53E-02 | down | 6.88 | 1.70 |
|  | COKMCCJN_01556 | *lgrB* | 3.139 | 1.650 | 9.44E-03 | 3.89E-02 | up | 4.83 | 7.75 |
|  | COKMCCJN_01563 | COKMCCJN_01563 | 0.342 | -1.549 | 6.60E-05 | 7.38E-04 | down | 4.61 | 0.94 |
|  | COKMCCJN_01579 | *cirA* | 0.139 | -2.851 | 1.44E-09 | 7.20E-08 | down | 4.97 | 0.38 |
|  | COKMCCJN_01588 | *cysL* | 0.430 | -1.219 | 2.38E-03 | 1.32E-02 | down | 3.14 | 0.84 |
|  | COKMCCJN_01599 | COKMCCJN_01599 | 0.290 | -1.787 | 2.12E-03 | 1.20E-02 | down | 4.11 | 0.67 |
|  | COKMCCJN_01600 | COKMCCJN_01600 | 0.332 | -1.592 | 1.26E-04 | 1.26E-03 | down | 5.82 | 1.14 |
|  | COKMCCJN_01602 | COKMCCJN_01602 | 0.428 | -1.225 | 1.89E-03 | 1.10E-02 | down | 3.57 | 0.90 |
|  | COKMCCJN_01603 | COKMCCJN_01603 | 0.362 | -1.465 | 2.90E-04 | 2.46E-03 | down | 6.97 | 1.48 |
|  | COKMCCJN_01634 | *cynR* | 0.229 | -2.124 | 2.86E-07 | 7.49E-06 | down | 3.78 | 0.53 |
|  | COKMCCJN_01640 | COKMCCJN_01640 | 2.189 | 1.130 | 4.42E-03 | 2.12E-02 | up | 1.88 | 2.64 |
|  | COKMCCJN_01647 | *phoE* | 3.600 | 1.848 | 1.67E-07 | 4.74E-06 | up | 8.54 | 19.65 |
|  | COKMCCJN_01659 | COKMCCJN_01659 | 0.488 | -1.035 | 2.14E-03 | 1.21E-02 | down | 19.16 | 6.02 |
|  | COKMCCJN_01660 | COKMCCJN_01660 | 0.385 | -1.377 | 8.60E-04 | 5.97E-03 | down | 3.82 | 0.92 |
|  | COKMCCJN_01664 | COKMCCJN_01664 | 2.136 | 1.095 | 5.81E-03 | 2.63E-02 | up | 1.10 | 1.59 |
|  | COKMCCJN_01668 | *sbcB* | 0.376 | -1.412 | 1.86E-07 | 5.12E-06 | down | 20.14 | 4.74 |
|  | COKMCCJN_01671 | *cdd* | 0.497 | -1.007 | 5.27E-03 | 2.44E-02 | down | 84.38 | 27.67 |
|  | COKMCCJN_01687 | COKMCCJN_01687 | 2.275 | 1.186 | 4.92E-03 | 2.32E-02 | up | 0.85 | 1.21 |
|  | COKMCCJN_01689 | COKMCCJN_01689 | 0.350 | -1.517 | 4.12E-03 | 2.00E-02 | down | 2.66 | 0.59 |
|  | COKMCCJN_01690 | *pctB* | 2.091 | 1.064 | 1.71E-03 | 1.02E-02 | up | 5.64 | 7.72 |
|  | COKMCCJN_01698 | *cdr* | 0.345 | -1.537 | 2.21E-04 | 1.96E-03 | down | 24.08 | 5.59 |
|  | COKMCCJN_01699 | COKMCCJN_01699 | 0.380 | -1.396 | 8.63E-03 | 3.63E-02 | down | 123.38 | 32.49 |
|  | COKMCCJN_01706 | *metQ* | 2.925 | 1.548 | 1.32E-04 | 1.29E-03 | up | 3.20 | 6.11 |
|  | COKMCCJN_01714 | COKMCCJN_01714 | 2.272 | 1.184 | 7.09E-03 | 3.10E-02 | up | 0.80 | 1.18 |
|  | COKMCCJN_01715 | COKMCCJN_01715 | 2.851 | 1.512 | 3.90E-04 | 3.12E-03 | up | 8.25 | 15.64 |
|  | COKMCCJN_01716 | COKMCCJN_01716 | 2.004 | 1.003 | 1.26E-03 | 8.07E-03 | up | 37.45 | 47.18 |
|  | COKMCCJN_01721 | *pheS* | 0.484 | -1.046 | 3.91E-03 | 1.93E-02 | down | 18.74 | 5.58 |
|  | COKMCCJN_01723 | COKMCCJN_01723 | 9.495 | 3.247 | 2.91E-06 | 5.58E-05 | up | 5.20 | 38.08 |
|  | COKMCCJN_01744 | COKMCCJN_01744 | 2.252 | 1.171 | 5.15E-03 | 2.39E-02 | up | 6121.23 | 9852.33 |
|  | COKMCCJN_01758 | *tyrP* | 2.303 | 1.203 | 3.43E-03 | 1.75E-02 | up | 0.75 | 1.06 |
|  | COKMCCJN_01761 | *dauA* | 3.004 | 1.587 | 1.57E-04 | 1.49E-03 | up | 0.37 | 0.70 |
|  | COKMCCJN_01766 | COKMCCJN_01766 | 8.598 | 3.104 | 1.53E-09 | 7.51E-08 | up | 1.98 | 12.67 |
|  | COKMCCJN_01769 | COKMCCJN_01769 | 0.373 | -1.421 | 1.06E-05 | 1.62E-04 | down | 28.81 | 6.96 |
|  | COKMCCJN_01770 | *polC* | 0.435 | -1.201 | 1.46E-05 | 2.14E-04 | down | 40.58 | 10.95 |
|  | COKMCCJN_01776 | COKMCCJN_01776 | 0.482 | -1.053 | 6.10E-04 | 4.44E-03 | down | 9.10 | 2.85 |
|  | COKMCCJN_01780 | *tfoX1* | 0.327 | -1.612 | 1.29E-06 | 2.76E-05 | down | 53.43 | 10.16 |
|  | COKMCCJN_01781 | COKMCCJN_01781 | 0.363 | -1.461 | 1.28E-04 | 1.28E-03 | down | 5.16 | 1.23 |
|  | COKMCCJN_01784 | *panP* | 0.376 | -1.412 | 1.25E-04 | 1.26E-03 | down | 5.85 | 1.29 |
|  | COKMCCJN_01799 | COKMCCJN_01799 | 0.495 | -1.016 | 7.07E-03 | 3.09E-02 | down | 26.17 | 8.28 |
|  | COKMCCJN_01803 | *radD* | 0.300 | -1.735 | 3.07E-05 | 4.03E-04 | down | 1.96 | 0.34 |
|  | COKMCCJN_01808 | COKMCCJN_01808 | 2.902 | 1.537 | 1.07E-02 | 4.30E-02 | up | 19.19 | 41.80 |
|  | COKMCCJN_01809 | COKMCCJN_01809 | 2.973 | 1.572 | 5.97E-03 | 2.69E-02 | up | 84.80 | 188.42 |
|  | COKMCCJN_01813 | COKMCCJN_01813 | 0.357 | -1.488 | 5.07E-03 | 2.37E-02 | down | 1.44 | 0.32 |
|  | COKMCCJN_01829 | COKMCCJN_01829 | 3.873 | 1.954 | 9.18E-06 | 1.44E-04 | up | 4.70 | 12.41 |
|  | COKMCCJN_01832 | COKMCCJN_01832 | 3.331 | 1.736 | 1.08E-03 | 7.20E-03 | up | 4.21 | 9.41 |
|  | COKMCCJN_01834 | COKMCCJN_01834 | 4.597 | 2.201 | 2.09E-06 | 4.10E-05 | up | 1.86 | 5.49 |
|  | COKMCCJN_01836 | COKMCCJN_01836 | 2.832 | 1.502 | 9.23E-04 | 6.29E-03 | up | 1.36 | 2.63 |
|  | COKMCCJN_01847 | COKMCCJN_01847 | 3.103 | 1.634 | 1.90E-05 | 2.69E-04 | up | 15.21 | 28.20 |
|  | COKMCCJN_01849 | COKMCCJN_01849 | 2.492 | 1.317 | 3.51E-05 | 4.47E-04 | up | 38.65 | 61.83 |
|  | COKMCCJN_01850 | COKMCCJN_01850 | 2.063 | 1.045 | 5.80E-03 | 2.63E-02 | up | 7.38 | 9.45 |
|  | COKMCCJN_01854 | *ynjB* | 4.350 | 2.121 | 3.26E-04 | 2.70E-03 | up | 128.09 | 438.15 |
|  | COKMCCJN_01855 | COKMCCJN_01855 | 2.855 | 1.513 | 2.50E-03 | 1.37E-02 | up | 2.61 | 5.20 |
|  | COKMCCJN_01871 | COKMCCJN_01871 | 9.697 | 3.277 | 2.68E-05 | 3.57E-04 | up | 1.33 | 8.09 |
|  | COKMCCJN_01878 | *tag* | 0.310 | -1.689 | 2.04E-07 | 5.54E-06 | down | 14.79 | 2.90 |
|  | COKMCCJN_01882 | *pspC* | 7.168 | 2.842 | 2.36E-11 | 1.82E-09 | up | 35.80 | 168.95 |
|  | COKMCCJN_01883 | *pspB* | 6.356 | 2.668 | 5.88E-07 | 1.37E-05 | up | 19.01 | 88.52 |
|  | COKMCCJN_01884 | *pspA* | 9.902 | 3.308 | 1.12E-12 | 1.23E-10 | up | 37.22 | 259.78 |
|  | COKMCCJN_01891 | COKMCCJN_01891 | 0.457 | -1.130 | 2.92E-03 | 1.54E-02 | down | 5.17 | 1.38 |
|  | COKMCCJN_01894 | COKMCCJN_01894 | 0.197 | -2.346 | 2.17E-04 | 1.93E-03 | down | 6.63 | 0.66 |
|  | COKMCCJN_01896 | *torC* | 3.302 | 1.724 | 1.50E-04 | 1.45E-03 | up | 6.95 | 16.58 |
|  | COKMCCJN_01901 | COKMCCJN_01901 | 0.393 | -1.349 | 4.48E-04 | 3.45E-03 | down | 99.68 | 25.82 |
|  | COKMCCJN_01903 | COKMCCJN_01903 | 0.487 | -1.039 | 1.18E-02 | 4.70E-02 | down | 2.84 | 0.87 |
|  | COKMCCJN_01910 | *hisI* | 0.308 | -1.700 | 7.48E-05 | 8.21E-04 | down | 59.18 | 11.02 |
|  | COKMCCJN_01911 | *hisF* | 0.266 | -1.912 | 6.47E-07 | 1.49E-05 | down | 39.82 | 6.59 |
|  | COKMCCJN_01912 | *hisA* | 0.364 | -1.459 | 8.94E-05 | 9.59E-04 | down | 29.37 | 6.49 |
|  | COKMCCJN_01913 | *hisH* | 0.280 | -1.837 | 7.41E-07 | 1.68E-05 | down | 72.49 | 12.77 |
|  | COKMCCJN_01914 | *hisB* | 0.300 | -1.737 | 3.85E-06 | 7.02E-05 | down | 55.32 | 10.51 |
|  | COKMCCJN_01915 | *hisC* | 0.288 | -1.796 | 1.09E-07 | 3.27E-06 | down | 50.88 | 9.30 |
|  | COKMCCJN_01916 | *hisD* | 0.355 | -1.495 | 1.82E-04 | 1.68E-03 | down | 40.28 | 9.43 |
|  | COKMCCJN_01917 | *hisG* | 0.478 | -1.064 | 2.54E-03 | 1.39E-02 | down | 62.63 | 19.52 |
|  | COKMCCJN_01927 | *bioD1* | 3.145 | 1.653 | 2.93E-06 | 5.59E-05 | up | 2.42 | 5.01 |
|  | COKMCCJN_01947 | *rpsA* | 4.197 | 2.069 | 5.94E-08 | 1.92E-06 | up | 174.19 | 429.31 |
|  | COKMCCJN_01962 | *ndh* | 0.492 | -1.023 | 4.47E-04 | 3.45E-03 | down | 14.19 | 4.42 |
|  | COKMCCJN_01963 | COKMCCJN_01963 | 0.381 | -1.393 | 2.83E-06 | 5.44E-05 | down | 110.31 | 26.18 |
|  | COKMCCJN_01968 | COKMCCJN_01968 | 0.390 | -1.357 | 3.16E-05 | 4.10E-04 | down | 48.72 | 12.45 |
|  | COKMCCJN_01972 | *zntA* | 2.472 | 1.305 | 2.30E-05 | 3.15E-04 | up | 19.24 | 30.51 |
|  | COKMCCJN_01978 | COKMCCJN_01978 | 0.383 | -1.385 | 2.81E-03 | 1.50E-02 | down | 35.43 | 9.16 |
|  | COKMCCJN_01979 | *ppiB* | 0.394 | -1.346 | 8.21E-04 | 5.75E-03 | down | 8.07 | 2.03 |
|  | COKMCCJN_01981 | *nemA* | 2.328 | 1.219 | 1.12E-03 | 7.38E-03 | up | 6.82 | 10.63 |
|  | COKMCCJN_01987 | COKMCCJN_01987 | 0.307 | -1.704 | 1.44E-05 | 2.13E-04 | down | 7.98 | 1.57 |
|  | COKMCCJN_01991 | *cyoE* | 2.317 | 1.212 | 1.72E-03 | 1.02E-02 | up | 6.96 | 9.16 |
|  | COKMCCJN_01992 | *cyoD* | 2.818 | 1.494 | 3.35E-03 | 1.72E-02 | up | 3.66 | 5.93 |
|  | COKMCCJN_01995 | *cyoA* | 2.146 | 1.101 | 7.66E-03 | 3.29E-02 | up | 12.32 | 14.90 |
|  | COKMCCJN_02007 | *leuO* | 0.286 | -1.804 | 1.65E-06 | 3.37E-05 | down | 15.46 | 2.63 |
|  | COKMCCJN_02009 | COKMCCJN_02009 | 2.797 | 1.484 | 1.58E-04 | 1.50E-03 | up | 0.48 | 0.87 |
|  | COKMCCJN_02014 | COKMCCJN_02014 | 0.457 | -1.129 | 8.90E-03 | 3.72E-02 | down | 3.60 | 1.04 |
|  | COKMCCJN_02025 | COKMCCJN_02025 | 2.608 | 1.383 | 1.17E-04 | 1.20E-03 | up | 2.32 | 3.94 |
|  | COKMCCJN_02035 | COKMCCJN_02035 | 7.034 | 2.814 | 2.50E-08 | 8.89E-07 | up | 25.10 | 113.71 |
|  | COKMCCJN_02068 | COKMCCJN_02068 | 0.401 | -1.318 | 2.60E-03 | 1.41E-02 | down | 35.60 | 9.12 |
|  | COKMCCJN_02071 | COKMCCJN_02071 | 4.863 | 2.282 | 1.24E-05 | 1.87E-04 | up | 4.69 | 14.33 |
|  | COKMCCJN_02082 | COKMCCJN_02082 | 0.432 | -1.211 | 4.66E-04 | 3.55E-03 | down | 14.08 | 3.75 |
|  | COKMCCJN_02086 | *ppiD* | 2.576 | 1.365 | 1.16E-05 | 1.76E-04 | up | 25.49 | 41.82 |
|  | COKMCCJN_02095 | COKMCCJN_02095 | 3.106 | 1.635 | 1.30E-05 | 1.95E-04 | up | 23.01 | 48.17 |
|  | COKMCCJN_02096 | *dctM* | 2.378 | 1.250 | 1.65E-03 | 9.89E-03 | up | 0.92 | 1.35 |
|  | COKMCCJN_02097 | *dctQ* | 7.568 | 2.920 | 1.62E-05 | 2.33E-04 | up | 0.19 | 0.82 |
|  | COKMCCJN_02098 | *dctP* | 23.303 | 4.542 | 3.57E-12 | 3.23E-10 | up | 0.71 | 8.35 |
|  | COKMCCJN_02106 | *msmX* | 3.141 | 1.651 | 5.68E-04 | 4.18E-03 | up | 0.76 | 1.65 |
|  | COKMCCJN_02120 | *fadI* | 0.393 | -1.346 | 1.32E-03 | 8.30E-03 | down | 9.66 | 2.16 |
|  | COKMCCJN_02121 | COKMCCJN_02121 | 6.426 | 2.684 | 3.78E-05 | 4.76E-04 | up | 399.32 | 1969.50 |
|  | COKMCCJN_02122 | COKMCCJN_02122 | 5.684 | 2.507 | 2.67E-05 | 3.57E-04 | up | 268.66 | 1168.33 |
|  | COKMCCJN_02124 | COKMCCJN_02124 | 2.204 | 1.140 | 6.06E-03 | 2.72E-02 | up | 2.78 | 4.30 |
|  | COKMCCJN_02126 | COKMCCJN_02126 | 2.180 | 1.125 | 1.29E-04 | 1.28E-03 | up | 13.60 | 18.42 |
|  | COKMCCJN_02134 | *ccmA* | 0.410 | -1.287 | 1.28E-02 | 4.97E-02 | down | 2.86 | 0.75 |
|  | COKMCCJN_02145 | *ftsY* | 2.265 | 1.179 | 4.96E-04 | 3.74E-03 | up | 5.07 | 6.79 |
|  | COKMCCJN_02155 | COKMCCJN_02155 | 2.359 | 1.238 | 8.48E-06 | 1.35E-04 | up | 13.17 | 20.00 |
|  | COKMCCJN_02167 | COKMCCJN_02167 | 2.403 | 1.265 | 6.35E-05 | 7.16E-04 | up | 10.33 | 16.34 |
|  | COKMCCJN_02168 | COKMCCJN_02168 | 2.830 | 1.501 | 1.41E-05 | 2.09E-04 | up | 145.07 | 269.66 |
|  | COKMCCJN_02169 | *flaB* | 3.105 | 1.634 | 4.01E-04 | 3.17E-03 | up | 34.61 | 64.11 |
|  | COKMCCJN_02170 | *flaD* | 3.459 | 1.790 | 4.25E-05 | 5.23E-04 | up | 15.25 | 32.31 |
|  | COKMCCJN_02171 | *flaB* | 5.087 | 2.347 | 2.05E-15 | 4.27E-13 | up | 60.10 | 188.25 |
|  | COKMCCJN_02172 | COKMCCJN_02172 | 2.543 | 1.346 | 3.67E-05 | 4.65E-04 | up | 2.70 | 4.45 |
|  | COKMCCJN_02173 | COKMCCJN_02173 | 2.935 | 1.553 | 1.70E-07 | 4.74E-06 | up | 3.21 | 6.13 |
|  | COKMCCJN_02174 | *flgJ* | 2.567 | 1.360 | 4.85E-06 | 8.38E-05 | up | 4.43 | 7.30 |
|  | COKMCCJN_02175 | *flgI* | 3.267 | 1.708 | 1.51E-08 | 5.62E-07 | up | 2.98 | 6.17 |
|  | COKMCCJN_02177 | *flgG* | 2.198 | 1.136 | 1.58E-03 | 9.52E-03 | up | 5.19 | 7.24 |
|  | COKMCCJN_02178 | *flgF* | 4.392 | 2.135 | 3.65E-06 | 6.75E-05 | up | 2.87 | 8.77 |
|  | COKMCCJN_02179 | *flgE* | 3.184 | 1.671 | 5.97E-09 | 2.54E-07 | up | 21.90 | 44.47 |
|  | COKMCCJN_02180 | COKMCCJN_02180 | 2.934 | 1.553 | 9.26E-09 | 3.78E-07 | up | 34.76 | 64.01 |
|  | COKMCCJN_02181 | *flgC* | 2.261 | 1.177 | 1.23E-04 | 1.25E-03 | up | 69.93 | 102.01 |
|  | COKMCCJN_02182 | *flgB* | 2.493 | 1.318 | 6.65E-05 | 7.41E-04 | up | 251.14 | 392.69 |
|  | COKMCCJN_02188 | COKMCCJN_02188 | 3.011 | 1.590 | 2.58E-07 | 6.83E-06 | up | 6.93 | 12.95 |
|  | COKMCCJN_02189 | COKMCCJN_02189 | 3.999 | 2.000 | 4.69E-10 | 2.71E-08 | up | 4.76 | 12.53 |
|  | COKMCCJN_02197 | *acpP* | 0.343 | -1.546 | 8.67E-05 | 9.37E-04 | down | 70.38 | 16.10 |
|  | COKMCCJN_02201 | COKMCCJN_02201 | 0.349 | -1.519 | 1.07E-04 | 1.11E-03 | down | 9.84 | 2.21 |
|  | COKMCCJN_02212 | COKMCCJN_02212 | 0.420 | -1.251 | 1.94E-03 | 1.12E-02 | down | 13.27 | 3.23 |
|  | COKMCCJN_02220 | COKMCCJN_02220 | 25.277 | 4.660 | 1.03E-23 | 8.54E-21 | up | 42.28 | 700.70 |
|  | COKMCCJN_02231 | *lolB* | 0.323 | -1.631 | 5.17E-04 | 3.88E-03 | down | 5.91 | 1.16 |
|  | COKMCCJN_02273 | *dacA* | 0.421 | -1.247 | 3.10E-05 | 4.05E-04 | down | 29.07 | 7.76 |
|  | COKMCCJN_02277 | COKMCCJN_02277 | 5.142 | 2.362 | 9.79E-08 | 3.00E-06 | up | 1.10 | 3.97 |
|  | COKMCCJN_02282 | *pleD* | 0.445 | -1.168 | 8.35E-04 | 5.82E-03 | down | 7.59 | 2.20 |
|  | COKMCCJN_02283 | COKMCCJN_02283 | 6.294 | 2.654 | 1.54E-07 | 4.45E-06 | up | 17.33 | 74.76 |
|  | COKMCCJN_02284 | *apbE* | 6.314 | 2.659 | 2.11E-05 | 2.94E-04 | up | 18.97 | 92.33 |
|  | COKMCCJN_02292 | *rlmG* | 0.382 | -1.389 | 2.96E-07 | 7.66E-06 | down | 47.99 | 11.64 |
|  | COKMCCJN_02295 | *ampG* | 0.422 | -1.244 | 6.91E-05 | 7.65E-04 | down | 4.54 | 1.17 |
|  | COKMCCJN_02296 | COKMCCJN_02296 | 3.309 | 1.726 | 7.58E-04 | 5.41E-03 | up | 3.25 | 7.00 |
|  | COKMCCJN_02309 | COKMCCJN_02309 | 11.478 | 3.521 | 1.63E-03 | 9.77E-03 | up | 6.04 | 56.62 |
|  | COKMCCJN_02335 | *nadK* | 0.361 | -1.470 | 3.25E-06 | 6.07E-05 | down | 25.35 | 6.08 |
|  | COKMCCJN_02340 | *smpB* | 0.486 | -1.040 | 1.11E-03 | 7.37E-03 | down | 68.46 | 21.83 |
|  | COKMCCJN_02346 | COKMCCJN_02346 | 5.077 | 2.344 | 1.63E-05 | 2.33E-04 | up | 0.44 | 1.57 |
|  | COKMCCJN_02352 | COKMCCJN_02352 | 0.323 | -1.632 | 5.72E-05 | 6.61E-04 | down | 4.22 | 0.88 |
|  | COKMCCJN_02356 | *satP* | 2.080 | 1.057 | 1.43E-03 | 8.84E-03 | up | 8.21 | 11.18 |
|  | COKMCCJN_02378 | *yfgJ* | 2.693 | 1.429 | 1.26E-04 | 1.26E-03 | up | 6.27 | 10.61 |
|  | COKMCCJN_02382 | *bamB* | 2.346 | 1.230 | 1.89E-04 | 1.72E-03 | up | 19.88 | 30.96 |
|  | COKMCCJN_02389 | *pepB* | 0.473 | -1.080 | 4.46E-04 | 3.45E-03 | down | 36.87 | 11.07 |
|  | COKMCCJN_02391 | *fdx* | 0.437 | -1.194 | 2.29E-03 | 1.28E-02 | down | 14.73 | 4.08 |
|  | COKMCCJN_02403 | *tgt* | 2.103 | 1.072 | 4.88E-04 | 3.69E-03 | up | 25.15 | 34.36 |
|  | COKMCCJN_02408 | *ahpC* | 0.428 | -1.224 | 6.44E-05 | 7.23E-04 | down | 523.14 | 135.33 |
|  | COKMCCJN_02419 | *pstS* | 0.369 | -1.440 | 2.87E-04 | 2.46E-03 | down | 6.80 | 1.49 |
|  | COKMCCJN_02420 | *phoR* | 0.415 | -1.268 | 7.65E-04 | 5.43E-03 | down | 10.12 | 2.45 |
|  | COKMCCJN_02433 | *clpB* | 2.843 | 1.508 | 1.54E-03 | 9.38E-03 | up | 123.54 | 251.24 |
|  | COKMCCJN_02434 | *yfiH* | 0.499 | -1.004 | 9.61E-03 | 3.95E-02 | down | 4.15 | 1.27 |
|  | COKMCCJN_02437 | *mltF* | 2.285 | 1.192 | 2.74E-04 | 2.36E-03 | up | 3.28 | 4.93 |
|  | COKMCCJN_02445 | COKMCCJN_02445 | 2.350 | 1.233 | 3.42E-03 | 1.75E-02 | up | 2.36 | 3.55 |
|  | COKMCCJN_02450 | *anmK* | 0.486 | -1.042 | 1.64E-03 | 9.82E-03 | down | 10.29 | 3.12 |
|  | COKMCCJN_02453 | *cstA* | 4.216 | 2.076 | 9.36E-05 | 9.97E-04 | up | 2.25 | 5.05 |
|  | COKMCCJN_02458 | *lspA* | 3.688 | 1.883 | 9.23E-11 | 6.09E-09 | up | 23.78 | 56.98 |
|  | COKMCCJN_02462 | *rpsT* | 2.429 | 1.280 | 5.10E-04 | 3.84E-03 | up | 17.88 | 25.68 |
|  | COKMCCJN_02464 | *nhaR* | 0.368 | -1.441 | 3.97E-04 | 3.15E-03 | down | 3.80 | 0.88 |
|  | COKMCCJN_02473 | COKMCCJN_02473 | 3.439 | 1.782 | 1.80E-06 | 3.61E-05 | up | 14.66 | 32.24 |
|  | COKMCCJN_02478 | *dsbC* | 2.525 | 1.336 | 9.21E-04 | 6.29E-03 | up | 40.37 | 70.58 |
|  | COKMCCJN_02481 | *brnQ* | 2.983 | 1.577 | 3.95E-03 | 1.95E-02 | up | 0.21 | 0.38 |
|  | COKMCCJN_02502 | COKMCCJN_02502 | 2.403 | 1.265 | 3.76E-03 | 1.88E-02 | up | 0.97 | 1.55 |
|  | COKMCCJN_02510 | *btuF* | 0.378 | -1.405 | 1.28E-03 | 8.15E-03 | down | 3.61 | 0.79 |
|  | COKMCCJN_02511 | COKMCCJN_02511 | 2.613 | 1.386 | 9.70E-04 | 6.58E-03 | up | 1.29 | 2.11 |
|  | COKMCCJN_02512 | COKMCCJN_02512 | 2.658 | 1.410 | 7.27E-04 | 5.25E-03 | up | 1.49 | 2.59 |
|  | COKMCCJN_02529 | *murE* | 0.489 | -1.031 | 1.44E-03 | 8.86E-03 | down | 9.99 | 2.90 |
|  | COKMCCJN_02538 | COKMCCJN_02538 | 2.543 | 1.346 | 3.87E-07 | 9.69E-06 | up | 50.55 | 81.24 |
|  | COKMCCJN_02543 | *rpsI* | 3.855 | 1.947 | 4.06E-07 | 1.01E-05 | up | 10.32 | 24.37 |
|  | COKMCCJN_02544 | *rplM* | 3.392 | 1.762 | 2.96E-07 | 7.66E-06 | up | 71.91 | 153.05 |
|  | COKMCCJN_02569 | *folB* | 0.341 | -1.552 | 2.05E-03 | 1.17E-02 | down | 5.82 | 1.21 |
|  | COKMCCJN_02574 | *yqeY* | 2.160 | 1.111 | 3.69E-03 | 1.86E-02 | up | 57.54 | 76.83 |
|  | COKMCCJN_02588 | COKMCCJN_02588 | 0.366 | -1.449 | 2.77E-03 | 1.48E-02 | down | 155.14 | 38.66 |
|  | COKMCCJN_02590 | *mdtC* | 2.708 | 1.437 | 1.11E-05 | 1.69E-04 | up | 4.42 | 7.97 |
|  | COKMCCJN_02591 | COKMCCJN_02591 | 4.001 | 2.000 | 1.52E-05 | 2.22E-04 | up | 10.38 | 26.55 |
|  | COKMCCJN_02592 | COKMCCJN_02592 | 3.029 | 1.599 | 5.16E-06 | 8.83E-05 | up | 8.41 | 17.01 |
|  | COKMCCJN_02593 | *leuA* | 0.448 | -1.158 | 3.63E-05 | 4.60E-04 | down | 26.61 | 7.56 |
|  | COKMCCJN_02595 | *leuC* | 0.397 | -1.331 | 3.97E-06 | 7.21E-05 | down | 96.60 | 23.06 |
|  | COKMCCJN_02596 | *leuD1* | 0.473 | -1.079 | 1.41E-03 | 8.75E-03 | down | 21.59 | 6.10 |
|  | COKMCCJN_02597 | COKMCCJN_02597 | 5.358 | 2.422 | 3.35E-04 | 2.75E-03 | up | 70.45 | 308.40 |
|  | COKMCCJN_02607 | COKMCCJN_02607 | 2.203 | 1.139 | 5.10E-03 | 2.38E-02 | up | 3.10 | 4.45 |
|  | COKMCCJN_02608 | *yjjP* | 3.454 | 1.788 | 3.00E-08 | 1.05E-06 | up | 3.36 | 7.45 |
|  | COKMCCJN_02610 | *rpmA* | 2.852 | 1.512 | 2.50E-03 | 1.37E-02 | up | 10.11 | 17.39 |
|  | COKMCCJN_02613 | COKMCCJN_02613 | 2.938 | 1.555 | 4.41E-03 | 2.11E-02 | up | 174.23 | 339.74 |
|  | COKMCCJN_02617 | COKMCCJN_02617 | 2.133 | 1.093 | 1.30E-04 | 1.28E-03 | up | 8.84 | 11.53 |
|  | COKMCCJN_02618 | *uspF* | 2.628 | 1.394 | 2.45E-05 | 3.33E-04 | up | 8.62 | 14.37 |
|  | COKMCCJN_02623 | COKMCCJN_02623 | 0.354 | -1.498 | 5.37E-06 | 9.12E-05 | down | 141.89 | 29.64 |
|  | COKMCCJN_02635 | COKMCCJN_02635 | 0.306 | -1.706 | 8.69E-03 | 3.65E-02 | down | 5.18 | 0.90 |
|  | COKMCCJN_02657 | *lysC* | 0.469 | -1.093 | 2.42E-03 | 1.34E-02 | down | 46.40 | 14.87 |
|  | COKMCCJN_02678 | COKMCCJN_02678 | 4.193 | 2.068 | 3.02E-12 | 2.86E-10 | up | 4.89 | 13.11 |
|  | COKMCCJN_02679 | COKMCCJN_02679 | 24.132 | 4.593 | 1.70E-07 | 4.74E-06 | up | 0.56 | 9.05 |
|  | COKMCCJN_02689 | *rplI* | 7.161 | 2.840 | 1.73E-14 | 2.88E-12 | up | 22.24 | 95.36 |
|  | COKMCCJN_02690 | *rpsR* | 6.747 | 2.754 | 2.34E-10 | 1.41E-08 | up | 50.75 | 202.98 |
|  | COKMCCJN_02691 | *rpsF* | 5.942 | 2.571 | 3.75E-14 | 5.57E-12 | up | 58.23 | 204.51 |
|  | COKMCCJN_02696 | *purA* | 0.490 | -1.030 | 2.14E-04 | 1.91E-03 | down | 33.20 | 10.15 |
|  | COKMCCJN_02705 | *tsaE* | 3.814 | 1.931 | 2.05E-06 | 4.05E-05 | up | 25.90 | 65.99 |
|  | COKMCCJN_02727 | *sdcS* | 2.239 | 1.163 | 4.16E-04 | 3.25E-03 | up | 2.43 | 3.22 |
|  | COKMCCJN_02731 | *frdA* | 4.591 | 2.199 | 1.39E-08 | 5.34E-07 | up | 62.64 | 171.20 |
|  | COKMCCJN_02732 | *frdB* | 4.983 | 2.317 | 9.40E-07 | 2.09E-05 | up | 46.40 | 137.11 |
|  | COKMCCJN_02733 | *frdC* | 4.845 | 2.276 | 8.88E-08 | 2.78E-06 | up | 10.64 | 30.63 |
|  | COKMCCJN_02734 | *frdD* | 6.330 | 2.662 | 1.78E-13 | 2.32E-11 | up | 17.37 | 66.00 |
|  | COKMCCJN_02736 | *epmB* | 0.465 | -1.106 | 8.31E-04 | 5.81E-03 | down | 3.88 | 1.16 |
|  | COKMCCJN_02757 | *ogt* | 0.335 | -1.577 | 8.34E-06 | 1.33E-04 | down | 11.40 | 2.29 |
|  | COKMCCJN_02787 | COKMCCJN_02787 | 2.629 | 1.395 | 1.53E-05 | 2.22E-04 | up | 489.75 | 824.67 |
|  | COKMCCJN_02793 | COKMCCJN_02793 | 0.380 | -1.395 | 1.52E-04 | 1.47E-03 | down | 19.07 | 4.90 |
|  | COKMCCJN_02797 | COKMCCJN_02797 | 0.367 | -1.448 | 1.34E-04 | 1.31E-03 | down | 12.27 | 2.63 |
|  | COKMCCJN_02798 | COKMCCJN_02798 | 0.358 | -1.482 | 2.74E-03 | 1.47E-02 | down | 9.37 | 1.90 |
|  | COKMCCJN_02799 | *bshA* | 0.441 | -1.181 | 1.19E-03 | 7.73E-03 | down | 20.72 | 5.32 |
|  | COKMCCJN_02803 | *waaA* | 0.490 | -1.029 | 3.00E-03 | 1.57E-02 | down | 3.12 | 0.93 |
|  | COKMCCJN_02810 | COKMCCJN_02810 | 0.456 | -1.134 | 6.24E-03 | 2.79E-02 | down | 15.33 | 4.81 |
|  | COKMCCJN_02812 | *rpmB* | 2.481 | 1.311 | 5.28E-04 | 3.93E-03 | up | 10.04 | 14.52 |
|  | COKMCCJN_02832 | COKMCCJN_02832 | 5.543 | 2.471 | 1.04E-10 | 6.68E-09 | up | 105.54 | 364.38 |
|  | COKMCCJN_02835 | *nfuA* | 0.435 | -1.201 | 3.88E-04 | 3.11E-03 | down | 23.09 | 6.75 |
|  | COKMCCJN_02836 | *nudE* | 0.483 | -1.049 | 3.46E-04 | 2.81E-03 | down | 79.05 | 23.26 |
|  | COKMCCJN_02837 | *cysQ* | 0.446 | -1.165 | 3.36E-04 | 2.76E-03 | down | 17.12 | 4.55 |
|  | COKMCCJN_02845 | *epsG* | 2.352 | 1.234 | 1.62E-04 | 1.53E-03 | up | 42.53 | 67.33 |
|  | COKMCCJN_02846 | *epsF* | 2.068 | 1.048 | 2.74E-04 | 2.36E-03 | up | 81.30 | 107.59 |
|  | COKMCCJN_02854 | COKMCCJN_02854 | 2.847 | 1.510 | 5.42E-07 | 1.29E-05 | up | 19.28 | 35.56 |
|  | COKMCCJN_02855 | *dtd* | 2.517 | 1.332 | 1.99E-03 | 1.14E-02 | up | 100.71 | 165.53 |
|  | COKMCCJN_02857 | COKMCCJN_02857 | 0.483 | -1.049 | 1.57E-03 | 9.48E-03 | down | 9.27 | 2.84 |
|  | COKMCCJN_02863 | *glnG* | 0.313 | -1.675 | 1.14E-02 | 4.55E-02 | down | 9.50 | 2.20 |
|  | COKMCCJN_02868 | COKMCCJN_02868 | 2.149 | 1.104 | 3.01E-03 | 1.58E-02 | up | 6.15 | 8.94 |
|  | COKMCCJN_02885 | *uspB* | 2.456 | 1.296 | 2.15E-03 | 1.21E-02 | up | 22.46 | 37.88 |
|  | COKMCCJN_02889 | COKMCCJN_02889 | 0.389 | -1.361 | 1.75E-03 | 1.03E-02 | down | 69.52 | 16.14 |
|  | COKMCCJN_02896 | COKMCCJN_02896 | 2.922 | 1.547 | 5.51E-03 | 2.53E-02 | up | 100.25 | 195.77 |
|  | COKMCCJN_02897 | *nqrB* | 3.159 | 1.659 | 5.80E-05 | 6.69E-04 | up | 4.63 | 9.53 |
|  | COKMCCJN_02898 | COKMCCJN_02898 | 2.725 | 1.446 | 2.43E-05 | 3.31E-04 | up | 19.49 | 34.05 |
|  | COKMCCJN_02910 | COKMCCJN_02910 | 4.076 | 2.027 | 7.08E-10 | 3.83E-08 | up | 13.88 | 35.69 |
|  | COKMCCJN_02912 | *bepE* | 2.067 | 1.048 | 3.42E-03 | 1.75E-02 | up | 54.47 | 66.38 |
|  | COKMCCJN_02926 | *aroE* | 0.447 | -1.160 | 3.18E-03 | 1.65E-02 | down | 4.86 | 1.42 |
|  | COKMCCJN_02931 | *topA* | 2.195 | 1.134 | 8.14E-03 | 3.47E-02 | up | 18.87 | 27.68 |
|  | COKMCCJN_02933 | COKMCCJN_02933 | 4.261 | 2.091 | 5.99E-05 | 6.85E-04 | up | 11.11 | 33.39 |
|  | COKMCCJN_02940 | COKMCCJN_02940 | 0.473 | -1.080 | 1.01E-03 | 6.76E-03 | down | 13.12 | 3.71 |
|  | COKMCCJN_02943 | COKMCCJN_02943 | 4.001 | 2.000 | 1.69E-11 | 1.35E-09 | up | 658.56 | 1691.60 |
|  | COKMCCJN_02944 | *yihD* | 0.491 | -1.027 | 3.97E-03 | 1.95E-02 | down | 59.45 | 18.56 |
|  | COKMCCJN_02947 | *dsbA* | 2.550 | 1.351 | 3.05E-06 | 5.79E-05 | up | 31.70 | 51.59 |
|  | COKMCCJN_02950 | *ilvG* | 0.491 | -1.025 | 3.51E-04 | 2.84E-03 | down | 28.97 | 9.09 |
|  | COKMCCJN_02952 | *ilvE* | 0.489 | -1.031 | 1.02E-03 | 6.82E-03 | down | 6.81 | 2.07 |
|  | COKMCCJN_02975 | COKMCCJN_02975 | 5.597 | 2.485 | 4.91E-07 | 1.19E-05 | up | 318.49 | 1032.36 |
|  | COKMCCJN_02979 | COKMCCJN_02979 | 4.621 | 2.208 | 1.10E-06 | 2.39E-05 | up | 61.86 | 167.21 |
|  | COKMCCJN_02980 | COKMCCJN_02980 | 5.767 | 2.528 | 3.97E-03 | 1.95E-02 | up | 1.20 | 4.44 |
|  | COKMCCJN_02981 | COKMCCJN_02981 | 5.076 | 2.344 | 1.59E-05 | 2.30E-04 | up | 4.36 | 12.67 |
|  | COKMCCJN_02982 | COKMCCJN_02982 | 10.658 | 3.414 | 9.46E-11 | 6.15E-09 | up | 8.50 | 51.54 |
|  | COKMCCJN_02983 | *yegS* | 9.605 | 3.264 | 4.01E-09 | 1.76E-07 | up | 0.53 | 2.82 |
|  | COKMCCJN_02984 | *mscS* | 5.543 | 2.471 | 1.16E-09 | 5.94E-08 | up | 0.93 | 3.22 |
|  | COKMCCJN_02985 | COKMCCJN_02985 | 4.397 | 2.137 | 2.03E-05 | 2.83E-04 | up | 1.72 | 4.61 |
|  | COKMCCJN_02986 | COKMCCJN_02986 | 6.105 | 2.610 | 7.46E-10 | 3.98E-08 | up | 1.84 | 6.92 |
|  | COKMCCJN_02987 | COKMCCJN_02987 | 7.085 | 2.825 | 7.99E-11 | 5.45E-09 | up | 47.53 | 200.64 |
|  | COKMCCJN_02988 | COKMCCJN_02988 | 8.387 | 3.068 | 1.44E-18 | 4.29E-16 | up | 6.49 | 33.97 |
|  | COKMCCJN_02989 | COKMCCJN_02989 | 13.208 | 3.723 | 4.80E-06 | 8.36E-05 | up | 0.57 | 4.75 |
|  | COKMCCJN_02990 | *tqsA* | 5.002 | 2.322 | 6.31E-09 | 2.65E-07 | up | 1.09 | 3.37 |
|  | COKMCCJN_02992 | *maa* | 3.262 | 1.706 | 6.24E-06 | 1.03E-04 | up | 2.87 | 6.19 |
|  | COKMCCJN_03004 | COKMCCJN_03004 | 0.481 | -1.056 | 9.10E-03 | 3.78E-02 | down | 12.23 | 3.50 |
|  | COKMCCJN_03005 | *yjcS* | 3.655 | 1.870 | 9.94E-08 | 3.00E-06 | up | 1.69 | 4.08 |
|  | COKMCCJN_03016 | COKMCCJN_03016 | 4.145 | 2.052 | 6.01E-03 | 2.71E-02 | up | 0.34 | 0.95 |
|  | COKMCCJN_03017 | COKMCCJN_03017 | 18.493 | 4.209 | 1.11E-04 | 1.15E-03 | up | 3.22 | 38.83 |
|  | COKMCCJN_03019 | COKMCCJN_03019 | 3.147 | 1.654 | 1.10E-03 | 7.31E-03 | up | 0.61 | 1.18 |
|  | COKMCCJN_03022 | COKMCCJN_03022 | 2.882 | 1.527 | 6.71E-06 | 1.10E-04 | up | 8.37 | 15.75 |
|  | COKMCCJN_03023 | COKMCCJN_03023 | 0.305 | -1.711 | 7.64E-08 | 2.43E-06 | down | 37.18 | 7.32 |
|  | COKMCCJN_03032 | *glpD* | 0.319 | -1.650 | 3.00E-07 | 7.71E-06 | down | 125.41 | 25.76 |
|  | COKMCCJN_03033 | *glpR* | 0.277 | -1.854 | 4.72E-04 | 3.58E-03 | down | 2.43 | 0.40 |
|  | COKMCCJN_03035 | *glpF* | 0.315 | -1.668 | 1.02E-03 | 6.84E-03 | down | 3.35 | 0.63 |
|  | COKMCCJN_03052 | *glpR* | 0.437 | -1.195 | 4.85E-03 | 2.30E-02 | down | 4.26 | 1.27 |
|  | COKMCCJN_03055 | *otnC* | 0.339 | -1.562 | 1.74E-04 | 1.62E-03 | down | 5.31 | 1.10 |
|  | COKMCCJN_03066 | COKMCCJN_03066 | 2.149 | 1.104 | 4.58E-04 | 3.50E-03 | up | 1.47 | 1.97 |
|  | COKMCCJN_03068 | *lamB* | 3.747 | 1.906 | 1.46E-08 | 5.48E-07 | up | 1.33 | 3.25 |
|  | COKMCCJN_03079 | *ttrA* | 4.562 | 2.190 | 6.99E-11 | 5.01E-09 | up | 1.90 | 5.57 |
|  | COKMCCJN_03080 | *ttrC* | 3.831 | 1.938 | 4.82E-05 | 5.76E-04 | up | 0.64 | 1.57 |
|  | COKMCCJN_03081 | *ttrB* | 2.833 | 1.502 | 4.70E-04 | 3.57E-03 | up | 4.80 | 8.98 |
|  | COKMCCJN_03083 | *ttrR* | 3.839 | 1.941 | 1.95E-06 | 3.88E-05 | up | 20.62 | 56.48 |
|  | COKMCCJN_03084 | COKMCCJN_03084 | 2.662 | 1.413 | 7.71E-04 | 5.46E-03 | up | 9.35 | 17.80 |
|  | COKMCCJN_03089 | COKMCCJN_03089 | 2.946 | 1.559 | 2.37E-04 | 2.08E-03 | up | 4.19 | 7.15 |
|  | COKMCCJN_03096 | *ykoD* | 2.389 | 1.256 | 2.09E-04 | 1.87E-03 | up | 0.98 | 1.44 |
|  | COKMCCJN_03098 | *yiaV* | 0.460 | -1.120 | 1.26E-04 | 1.26E-03 | down | 9.09 | 2.56 |
|  | COKMCCJN_03099 | *yiaW* | 0.463 | -1.110 | 5.39E-03 | 2.49E-02 | down | 9.82 | 2.80 |
|  | COKMCCJN_03103 | COKMCCJN_03103 | 0.484 | -1.048 | 2.36E-04 | 2.08E-03 | down | 13.52 | 4.01 |
|  | COKMCCJN_03110 | *lifO* | 0.395 | -1.341 | 1.06E-02 | 4.27E-02 | down | 1.50 | 0.36 |
|  | COKMCCJN_03114 | COKMCCJN_03114 | 3.445 | 1.784 | 1.88E-03 | 1.10E-02 | up | 0.17 | 0.34 |
|  | COKMCCJN_03128 | *mcpH* | 4.200 | 2.070 | 4.03E-06 | 7.29E-05 | up | 11.51 | 30.72 |
|  | COKMCCJN_03132 | *malE* | 2.277 | 1.187 | 7.34E-05 | 8.08E-04 | up | 8.16 | 11.56 |
|  | COKMCCJN_03134 | *malG* | 5.022 | 2.328 | 9.69E-13 | 1.09E-10 | up | 9.08 | 29.73 |
|  | COKMCCJN_03138 | *nimR* | 0.409 | -1.291 | 3.29E-03 | 1.70E-02 | down | 3.07 | 0.82 |
|  | COKMCCJN_03152 | *bceA* | 2.345 | 1.229 | 1.25E-03 | 8.01E-03 | up | 29.69 | 43.47 |
|  | COKMCCJN_03154 | *cntO* | 2.194 | 1.134 | 9.22E-04 | 6.29E-03 | up | 1.04 | 1.46 |
|  | COKMCCJN_03160 | COKMCCJN_03160 | 0.492 | -1.024 | 2.78E-03 | 1.48E-02 | down | 2.82 | 0.87 |
|  | COKMCCJN_03162 | *kduD* | 3.408 | 1.769 | 2.15E-03 | 1.21E-02 | up | 0.33 | 0.75 |
|  | COKMCCJN_03178 | COKMCCJN_03178 | 0.426 | -1.230 | 1.31E-03 | 8.26E-03 | down | 76.06 | 18.65 |
|  | COKMCCJN_03179 | COKMCCJN_03179 | 0.233 | -2.100 | 1.47E-03 | 9.05E-03 | down | 98.50 | 15.77 |
|  | COKMCCJN_03180 | *pcpR* | 0.255 | -1.973 | 2.05E-06 | 4.05E-05 | down | 8.90 | 1.50 |
|  | COKMCCJN_03181 | *glcB* | 0.271 | -1.884 | 1.71E-04 | 1.60E-03 | down | 120.97 | 21.45 |
|  | COKMCCJN_03202 | *hmp* | 0.442 | -1.179 | 5.41E-04 | 4.01E-03 | down | 5.82 | 1.58 |
|  | COKMCCJN_03212 | *hmuV* | 0.272 | -1.881 | 2.56E-03 | 1.39E-02 | down | 18.32 | 2.51 |
|  | COKMCCJN_03213 | *hmuU* | 0.231 | -2.115 | 1.60E-03 | 9.64E-03 | down | 5.54 | 0.65 |
|  | COKMCCJN_03214 | *btuF* | 0.165 | -2.602 | 7.35E-06 | 1.19E-04 | down | 52.35 | 4.48 |
|  | COKMCCJN_03215 | *tolR* | 0.254 | -1.977 | 2.69E-03 | 1.45E-02 | down | 9.05 | 1.19 |
|  | COKMCCJN_03216 | *tolQ* | 0.234 | -2.096 | 1.99E-04 | 1.80E-03 | down | 14.37 | 1.85 |
|  | COKMCCJN_03217 | COKMCCJN_03217 | 0.219 | -2.194 | 4.42E-06 | 7.95E-05 | down | 28.78 | 3.42 |
|  | COKMCCJN_03222 | *cheV* | 0.478 | -1.066 | 9.08E-04 | 6.23E-03 | down | 41.59 | 13.01 |
|  | COKMCCJN_03225 | *pctA* | 2.363 | 1.241 | 1.31E-04 | 1.29E-03 | up | 6.56 | 9.61 |
|  | COKMCCJN_03230 | COKMCCJN_03230 | 0.493 | -1.021 | 4.50E-04 | 3.45E-03 | down | 234.53 | 75.90 |
|  | COKMCCJN_03232 | *mepM* | 0.441 | -1.181 | 1.24E-03 | 7.99E-03 | down | 5.25 | 1.39 |
|  | COKMCCJN_03240 | *ompL* | 0.268 | -1.897 | 9.64E-07 | 2.13E-05 | down | 1963.49 | 302.51 |
|  | COKMCCJN_03241 | *doeB* | 0.498 | -1.006 | 4.78E-03 | 2.27E-02 | down | 4.09 | 1.27 |
|  | COKMCCJN_03244 | *dsbD* | 2.049 | 1.035 | 6.12E-03 | 2.74E-02 | up | 34.30 | 45.89 |
|  | COKMCCJN_03248 | COKMCCJN_03248 | 2.361 | 1.239 | 1.27E-03 | 8.13E-03 | up | 4.51 | 7.15 |
|  | COKMCCJN_03260 | *ydhF* | 5.194 | 2.377 | 1.06E-11 | 9.15E-10 | up | 53.80 | 190.35 |
|  | COKMCCJN_03262 | COKMCCJN_03262 | 2.264 | 1.179 | 9.88E-03 | 4.04E-02 | up | 17.11 | 26.82 |
|  | COKMCCJN_03268 | COKMCCJN_03268 | 2.407 | 1.267 | 5.08E-03 | 2.37E-02 | up | 3.29 | 5.30 |
|  | COKMCCJN_03271 | COKMCCJN_03271 | 2.551 | 1.351 | 2.65E-05 | 3.56E-04 | up | 64.00 | 107.16 |
|  | COKMCCJN_03272 | *pepT* | 3.297 | 1.721 | 2.18E-06 | 4.26E-05 | up | 3.03 | 6.63 |
|  | COKMCCJN_03284 | *ackA* | 5.497 | 2.459 | 2.90E-08 | 1.02E-06 | up | 7.20 | 28.91 |
|  | COKMCCJN_03307 | COKMCCJN_03307 | 2.293 | 1.197 | 4.02E-04 | 3.17E-03 | up | 3.33 | 4.80 |
|  | COKMCCJN_03319 | COKMCCJN_03319 | 0.471 | -1.085 | 8.81E-04 | 6.08E-03 | down | 7.96 | 2.32 |
|  | COKMCCJN_03325 | COKMCCJN_03325 | 2.219 | 1.150 | 1.42E-03 | 8.79E-03 | up | 10.40 | 15.56 |
|  | COKMCCJN_03340 | *ampR* | 0.381 | -1.394 | 2.51E-04 | 2.19E-03 | down | 3.21 | 0.77 |
|  | COKMCCJN_03343 | COKMCCJN_03343 | 2.915 | 1.543 | 4.45E-06 | 7.97E-05 | up | 8.37 | 15.83 |
|  | COKMCCJN_03346 | *guaC* | 0.380 | -1.397 | 1.28E-04 | 1.28E-03 | down | 17.27 | 3.90 |
|  | COKMCCJN_03349 | *pal* | 0.417 | -1.261 | 1.75E-03 | 1.04E-02 | down | 17.58 | 4.43 |
|  | COKMCCJN_03364 | *thiD* | 2.041 | 1.029 | 5.54E-03 | 2.54E-02 | up | 1.82 | 2.37 |
|  | COKMCCJN_03367 | COKMCCJN_03367 | 2.634 | 1.397 | 1.55E-04 | 1.49E-03 | up | 3.08 | 4.78 |
|  | COKMCCJN_03369 | *thiM* | 2.670 | 1.417 | 3.63E-04 | 2.93E-03 | up | 2.52 | 3.91 |
|  | COKMCCJN_03384 | *yclQ* | 0.207 | -2.273 | 7.54E-06 | 1.22E-04 | down | 8.55 | 1.01 |
|  | COKMCCJN_03386 | *yclO* | 0.192 | -2.379 | 1.23E-02 | 4.85E-02 | down | 0.76 | 0.08 |
|  | COKMCCJN_03387 | *yclP* | 0.238 | -2.071 | 8.83E-04 | 6.09E-03 | down | 4.08 | 0.51 |
|  | COKMCCJN_03392 | *prtV* | 2.827 | 1.499 | 1.14E-03 | 7.50E-03 | up | 0.26 | 0.43 |
|  | COKMCCJN_03395 | *lrp* | 0.378 | -1.403 | 1.79E-03 | 1.05E-02 | down | 4.94 | 1.18 |
|  | COKMCCJN_03398 | *tal* | 0.425 | -1.233 | 1.06E-04 | 1.11E-03 | down | 88.25 | 24.55 |
|  | COKMCCJN_03402 | COKMCCJN_03402 | 0.380 | -1.395 | 2.92E-04 | 2.48E-03 | down | 34.41 | 8.34 |
|  | COKMCCJN_03414 | COKMCCJN_03414 | 0.347 | -1.528 | 1.55E-03 | 9.38E-03 | down | 4.83 | 0.94 |
|  | COKMCCJN_03422 | COKMCCJN_03422 | 3.788 | 1.922 | 2.58E-07 | 6.83E-06 | up | 0.90 | 2.18 |
|  | COKMCCJN_03429 | COKMCCJN_03429 | 3.147 | 1.654 | 6.59E-03 | 2.92E-02 | up | 1.41 | 2.94 |
|  | COKMCCJN_03434 | *phnA* | 0.285 | -1.810 | 8.96E-04 | 6.16E-03 | down | 6.23 | 1.17 |
|  | COKMCCJN_03436 | COKMCCJN_03436 | 0.160 | -2.641 | 7.12E-03 | 3.11E-02 | down | 1.23 | 0.11 |
|  | COKMCCJN_03444 | *rhaS* | 0.417 | -1.262 | 2.57E-03 | 1.40E-02 | down | 14.50 | 4.19 |
|  | COKMCCJN_03449 | *rclC* | 5.460 | 2.449 | 1.78E-04 | 1.65E-03 | up | 14.01 | 60.25 |
|  | COKMCCJN_03455 | COKMCCJN_03455 | 0.453 | -1.141 | 3.43E-03 | 1.75E-02 | down | 9.61 | 2.91 |
|  | COKMCCJN_03465 | *pal* | 3.243 | 1.697 | 1.86E-07 | 5.12E-06 | up | 11.05 | 23.05 |
|  | COKMCCJN_03466 | COKMCCJN_03466 | 0.443 | -1.174 | 5.88E-03 | 2.66E-02 | down | 30.56 | 9.24 |
|  | COKMCCJN_03467 | *luxO* | 0.409 | -1.289 | 3.13E-03 | 1.63E-02 | down | 150.06 | 43.43 |
|  | COKMCCJN_03468 | *hmp* | 0.369 | -1.437 | 1.28E-04 | 1.28E-03 | down | 40.40 | 8.65 |
|  | COKMCCJN_03471 | COKMCCJN_03471 | 0.330 | -1.601 | 8.76E-05 | 9.44E-04 | down | 26.00 | 5.09 |
|  | COKMCCJN_03472 | COKMCCJN_03472 | 0.318 | -1.654 | 4.54E-05 | 5.51E-04 | down | 7.04 | 1.29 |
|  | COKMCCJN_03473 | COKMCCJN_03473 | 0.370 | -1.435 | 4.23E-04 | 3.30E-03 | down | 4.47 | 0.96 |
|  | COKMCCJN_03474 | *potA* | 0.265 | -1.916 | 4.10E-05 | 5.06E-04 | down | 3.36 | 0.52 |
|  | COKMCCJN_03475 | *cpdA* | 0.330 | -1.599 | 1.20E-04 | 1.22E-03 | down | 10.38 | 1.97 |
|  | COKMCCJN_03493 | COKMCCJN_03493 | 0.370 | -1.434 | 1.32E-04 | 1.29E-03 | down | 9.91 | 2.50 |
|  | COKMCCJN_03495 | *phaA* | 0.356 | -1.491 | 9.05E-05 | 9.68E-04 | down | 10.16 | 2.10 |
|  | COKMCCJN_03496 | COKMCCJN_03496 | 0.238 | -2.068 | 3.84E-06 | 7.02E-05 | down | 173.26 | 23.04 |
|  | COKMCCJN_03499 | *napC* | 3.397 | 1.764 | 5.86E-06 | 9.79E-05 | up | 13.96 | 31.46 |
|  | COKMCCJN_03500 | COKMCCJN_03500 | 2.820 | 1.496 | 1.29E-04 | 1.28E-03 | up | 25.16 | 48.62 |
|  | COKMCCJN_03506 | *dcuD* | 11.013 | 3.461 | 6.41E-14 | 8.90E-12 | up | 0.26 | 1.87 |
|  | COKMCCJN_03508 | COKMCCJN_03508 | 0.428 | -1.225 | 1.14E-02 | 4.55E-02 | down | 7.58 | 2.34 |
|  | COKMCCJN_03517 | *mngR* | 0.409 | -1.288 | 7.11E-04 | 5.15E-03 | down | 6.23 | 1.71 |
|  | COKMCCJN_03524 | COKMCCJN_03524 | 0.399 | -1.326 | 1.15E-04 | 1.19E-03 | down | 38.75 | 10.37 |
|  | COKMCCJN_03528 | COKMCCJN_03528 | 0.447 | -1.161 | 3.23E-03 | 1.68E-02 | down | 6.37 | 1.81 |
|  | COKMCCJN_03539 | *ravA* | 0.400 | -1.321 | 3.39E-05 | 4.36E-04 | down | 29.60 | 7.36 |
|  | COKMCCJN_03540 | *viaA* | 0.337 | -1.567 | 2.62E-06 | 5.07E-05 | down | 43.60 | 9.13 |
|  | COKMCCJN_03555 | *nasD* | 10.225 | 3.354 | 9.87E-16 | 2.16E-13 | up | 2.67 | 17.51 |
|  | COKMCCJN_03556 | *nirD* | 9.409 | 3.234 | 2.09E-07 | 5.65E-06 | up | 1.09 | 7.02 |
|  | COKMCCJN_03557 | *focA* | 5.422 | 2.439 | 1.39E-06 | 2.92E-05 | up | 1.84 | 6.78 |
|  | COKMCCJN_03563 | *gltP* | 3.244 | 1.698 | 4.53E-08 | 1.53E-06 | up | 4.18 | 8.15 |
|  | COKMCCJN_03565 | COKMCCJN_03565 | 0.362 | -1.466 | 3.97E-05 | 4.92E-04 | down | 14.49 | 3.13 |
|  | COKMCCJN_03566 | *tolQ* | 0.316 | -1.663 | 5.82E-05 | 6.69E-04 | down | 25.53 | 4.66 |
|  | COKMCCJN_03567 | COKMCCJN_03567 | 0.427 | -1.228 | 1.19E-02 | 4.71E-02 | down | 18.37 | 4.59 |
|  | COKMCCJN_03568 | *tolR* | 0.375 | -1.417 | 3.12E-03 | 1.62E-02 | down | 19.83 | 4.39 |
|  | COKMCCJN_03569 | COKMCCJN_03569 | 0.299 | -1.740 | 8.89E-05 | 9.56E-04 | down | 69.02 | 11.92 |
|  | COKMCCJN_03570 | COKMCCJN_03570 | 0.421 | -1.249 | 1.20E-03 | 7.79E-03 | down | 42.18 | 10.41 |
|  | COKMCCJN_03584 | *norM* | 3.266 | 1.708 | 2.34E-04 | 2.07E-03 | up | 0.29 | 0.58 |
|  | COKMCCJN_03593 | *fabH2* | 0.473 | -1.079 | 3.17E-04 | 2.65E-03 | down | 20.27 | 6.13 |
|  | COKMCCJN_03599 | *nrdD* | 3.775 | 1.917 | 1.63E-07 | 4.64E-06 | up | 0.93 | 2.31 |
|  | COKMCCJN_03601 | *tyrB* | 0.377 | -1.406 | 2.75E-05 | 3.65E-04 | down | 13.15 | 2.96 |
|  | COKMCCJN_03603 | *ybiT* | 2.230 | 1.157 | 4.70E-05 | 5.67E-04 | up | 3.70 | 5.13 |
|  | COKMCCJN_03605 | COKMCCJN_03605 | 3.230 | 1.691 | 3.11E-07 | 7.94E-06 | up | 6.39 | 13.64 |
|  | COKMCCJN_03611 | COKMCCJN_03611 | 2.126 | 1.088 | 1.19E-02 | 4.71E-02 | up | 5.57 | 7.23 |
|  | COKMCCJN_03622 | COKMCCJN_03622 | 2.461 | 1.299 | 6.75E-05 | 7.51E-04 | up | 9.27 | 14.32 |
|  | COKMCCJN_03626 | COKMCCJN_03626 | 0.330 | -1.599 | 1.24E-04 | 1.26E-03 | down | 5.28 | 1.07 |
|  | COKMCCJN_03639 | *xerC* | 0.496 | -1.012 | 1.90E-03 | 1.10E-02 | down | 6.98 | 2.14 |
|  | COKMCCJN_03642 | *patZ* | 0.400 | -1.322 | 1.91E-04 | 1.73E-03 | down | 16.28 | 3.76 |
|  | COKMCCJN_03644 | *hemR* | 0.261 | -1.936 | 4.40E-03 | 2.11E-02 | down | 31.09 | 4.00 |
|  | COKMCCJN_03651 | *lolD* | 2.705 | 1.436 | 1.20E-03 | 7.78E-03 | up | 4.04 | 6.97 |
|  | COKMCCJN_03653 | *fabV* | 2.271 | 1.183 | 3.04E-04 | 2.56E-03 | up | 42.33 | 61.17 |
|  | COKMCCJN_03662 | *sgrR* | 0.445 | -1.167 | 1.89E-04 | 1.72E-03 | down | 3.69 | 1.04 |
|  | COKMCCJN_03667 | COKMCCJN_03667 | 2.053 | 1.037 | 6.25E-03 | 2.79E-02 | up | 1.16 | 1.44 |
|  | COKMCCJN_03689 | COKMCCJN_03689 | 2.671 | 1.417 | 3.35E-04 | 2.75E-03 | up | 8.38 | 14.81 |
|  | COKMCCJN_03690 | COKMCCJN_03690 | 0.384 | -1.380 | 1.05E-05 | 1.62E-04 | down | 23.41 | 5.59 |
|  | COKMCCJN_03693 | *argP* | 0.359 | -1.480 | 7.17E-04 | 5.18E-03 | down | 21.07 | 5.21 |
|  | COKMCCJN_03699 | *cph2* | 0.348 | -1.523 | 6.92E-05 | 7.65E-04 | down | 11.59 | 2.60 |
|  | COKMCCJN_03700 | *czcA* | 2.552 | 1.351 | 3.87E-05 | 4.84E-04 | up | 1.54 | 2.62 |
|  | COKMCCJN_03707 | *tyrP* | 2.948 | 1.560 | 8.29E-03 | 3.52E-02 | up | 0.19 | 0.34 |
|  | COKMCCJN_03711 | COKMCCJN_03711 | 0.282 | -1.824 | 8.81E-06 | 1.40E-04 | down | 11.45 | 2.10 |
|  | COKMCCJN_03716 | *yqaB* | 0.383 | -1.384 | 8.11E-05 | 8.81E-04 | down | 169.75 | 42.18 |
|  | COKMCCJN_03717 | *arfB* | 0.418 | -1.260 | 1.85E-03 | 1.08E-02 | down | 5.29 | 1.42 |
|  | COKMCCJN_03729 | *norM* | 4.541 | 2.183 | 2.71E-05 | 3.60E-04 | up | 0.21 | 0.59 |
|  | COKMCCJN_03731 | *pctB* | 4.404 | 2.139 | 7.47E-11 | 5.27E-09 | up | 1.31 | 3.56 |
|  | COKMCCJN_03735 | COKMCCJN_03735 | 0.255 | -1.969 | 9.02E-06 | 1.42E-04 | down | 161.73 | 28.52 |
|  | COKMCCJN_03748 | COKMCCJN_03748 | 5.715 | 2.515 | 4.92E-03 | 2.32E-02 | up | 0.29 | 0.98 |
|  | COKMCCJN_03754 | COKMCCJN_03754 | 7.187 | 2.845 | 1.42E-11 | 1.16E-09 | up | 2.89 | 14.43 |
|  | COKMCCJN_03755 | COKMCCJN_03755 | 5.898 | 2.560 | 1.93E-10 | 1.20E-08 | up | 4.24 | 16.81 |
|  | COKMCCJN_03756 | COKMCCJN_03756 | 4.774 | 2.255 | 1.85E-06 | 3.71E-05 | up | 7.66 | 24.95 |
|  | COKMCCJN_03757 | *ltxD* | 3.123 | 1.643 | 1.86E-04 | 1.71E-03 | up | 4.02 | 8.77 |
|  | COKMCCJN_03758 | *rcsC* | 2.456 | 1.296 | 3.11E-04 | 2.61E-03 | up | 3.17 | 5.10 |
|  | COKMCCJN_03760 | *hlyB* | 2.252 | 1.171 | 9.98E-04 | 6.72E-03 | up | 3.15 | 4.56 |
|  | COKMCCJN_03761 | COKMCCJN_03761 | 2.176 | 1.122 | 2.04E-03 | 1.16E-02 | up | 2.22 | 3.07 |
|  | COKMCCJN_03772 | COKMCCJN_03772 | 2.673 | 1.418 | 3.28E-04 | 2.71E-03 | up | 1.78 | 3.24 |
|  | COKMCCJN_03791 | COKMCCJN_03791 | 4.363 | 2.125 | 1.35E-05 | 2.01E-04 | up | 26.30 | 75.50 |
|  | COKMCCJN_03803 | COKMCCJN_03803 | 3.200 | 1.678 | 4.98E-03 | 2.34E-02 | up | 17.18 | 34.67 |
|  | COKMCCJN_03807 | COKMCCJN_03807 | 2.187 | 1.129 | 1.37E-03 | 8.59E-03 | up | 40.27 | 55.40 |
|  | COKMCCJN_03811 | COKMCCJN_03811 | 2.028 | 1.020 | 1.00E-02 | 4.08E-02 | up | 1.39 | 1.88 |
|  | COKMCCJN_03814 | *aldHT* | 0.360 | -1.473 | 1.06E-04 | 1.11E-03 | down | 4.49 | 1.03 |
|  | COKMCCJN_03815 | *yabJ* | 0.119 | -3.072 | 4.41E-04 | 3.42E-03 | down | 2.33 | 0.21 |
|  | COKMCCJN_03817 | COKMCCJN_03817 | 0.206 | -2.277 | 4.33E-07 | 1.06E-05 | down | 24.93 | 3.58 |
|  | COKMCCJN_03818 | *atpB* | 0.130 | -2.941 | 2.65E-09 | 1.21E-07 | down | 54.93 | 4.70 |
|  | COKMCCJN_03819 | *atpE* | 0.089 | -3.484 | 1.24E-07 | 3.65E-06 | down | 20.16 | 1.23 |
|  | COKMCCJN_03820 | *atpF* | 0.102 | -3.293 | 3.34E-12 | 3.08E-10 | down | 70.59 | 4.94 |
|  | COKMCCJN_03821 | *atpH* | 0.151 | -2.723 | 3.31E-09 | 1.46E-07 | down | 17.56 | 1.82 |
|  | COKMCCJN_03822 | *atpA* | 0.096 | -3.386 | 8.24E-21 | 3.81E-18 | down | 40.99 | 2.68 |
|  | COKMCCJN_03823 | *atpG* | 0.101 | -3.309 | 8.55E-15 | 1.48E-12 | down | 43.78 | 3.17 |
|  | COKMCCJN_03824 | *atpD* | 0.108 | -3.206 | 1.45E-09 | 7.20E-08 | down | 40.88 | 3.40 |
|  | COKMCCJN_03825 | *atpC* | 0.147 | -2.770 | 1.74E-05 | 2.47E-04 | down | 17.46 | 2.05 |
|  | COKMCCJN_03829 | *yejB* | 3.816 | 1.932 | 9.50E-08 | 2.95E-06 | up | 24.20 | 63.17 |
|  | COKMCCJN_03831 | COKMCCJN_03831 | 0.470 | -1.088 | 4.32E-04 | 3.35E-03 | down | 7.72 | 2.26 |
|  | COKMCCJN_03841 | COKMCCJN_03841 | 10.017 | 3.324 | 6.75E-03 | 2.96E-02 | up | 9.49 | 80.00 |
|  | COKMCCJN_03859 | COKMCCJN_03859 | 3.423 | 1.775 | 5.33E-08 | 1.74E-06 | up | 9.85 | 21.88 |
|  | COKMCCJN_03863 | *nrtP* | 7.112 | 2.830 | 7.82E-07 | 1.77E-05 | up | 1.49 | 8.16 |
|  | COKMCCJN_03865 | *cra* | 11.244 | 3.491 | 3.02E-12 | 2.86E-10 | up | 0.98 | 6.80 |
|  | COKMCCJN_03866 | *fruB* | 14.739 | 3.882 | 5.12E-14 | 7.35E-12 | up | 7.91 | 74.14 |
|  | COKMCCJN_03867 | *lacC* | 14.998 | 3.907 | 2.82E-12 | 2.79E-10 | up | 5.39 | 49.94 |
|  | COKMCCJN_03868 | *fruA* | 12.834 | 3.682 | 1.37E-13 | 1.84E-11 | up | 13.45 | 102.38 |
|  | COKMCCJN_03873 | COKMCCJN_03873 | 4.032 | 2.011 | 6.56E-08 | 2.10E-06 | up | 0.76 | 1.91 |
|  | COKMCCJN_03874 | *fan1* | 0.411 | -1.282 | 3.46E-04 | 2.81E-03 | down | 4.65 | 1.22 |
|  | COKMCCJN_03881 | *torT* | 0.440 | -1.183 | 1.20E-03 | 7.77E-03 | down | 2.78 | 0.79 |
|  | COKMCCJN_03882 | *mgsA* | 0.325 | -1.620 | 5.39E-08 | 1.75E-06 | down | 40.64 | 8.42 |
|  | COKMCCJN_03888 | COKMCCJN_03888 | 2.069 | 1.049 | 4.37E-03 | 2.10E-02 | up | 1.37 | 1.78 |
|  | COKMCCJN_03899 | *adhB* | 2.178 | 1.123 | 3.98E-03 | 1.95E-02 | up | 3.02 | 3.94 |
|  | COKMCCJN_03904 | COKMCCJN_03904 | 0.455 | -1.136 | 7.42E-04 | 5.30E-03 | down | 25.57 | 7.64 |
|  | COKMCCJN_03909 | *dmlR* | 0.384 | -1.381 | 5.52E-04 | 4.08E-03 | down | 3.94 | 0.97 |
|  | COKMCCJN_03922 | COKMCCJN_03922 | 0.306 | -1.710 | 1.86E-03 | 1.09E-02 | down | 5.29 | 1.10 |
|  | COKMCCJN_03923 | COKMCCJN_03923 | 0.433 | -1.207 | 7.79E-03 | 3.33E-02 | down | 9.03 | 2.57 |
|  | COKMCCJN_03933 | *gndA* | 3.805 | 1.928 | 4.97E-07 | 1.19E-05 | up | 3.26 | 8.64 |
|  | COKMCCJN_03940 | *phnU2* | 0.324 | -1.625 | 1.66E-05 | 2.37E-04 | down | 4.40 | 0.84 |
|  | COKMCCJN_03941 | *phnT2* | 0.376 | -1.411 | 4.97E-05 | 5.92E-04 | down | 7.05 | 1.70 |
|  | COKMCCJN_03944 | *phnW* | 0.399 | -1.324 | 9.57E-05 | 1.01E-03 | down | 7.10 | 1.88 |
|  | COKMCCJN_03945 | *puuE* | 0.259 | -1.950 | 4.16E-08 | 1.43E-06 | down | 11.69 | 1.78 |
|  | COKMCCJN_03946 | *phnX* | 0.298 | -1.748 | 6.95E-05 | 7.67E-04 | down | 14.97 | 2.56 |
|  | COKMCCJN_03949 | *msrAB* | 5.238 | 2.389 | 1.29E-03 | 8.18E-03 | up | 332.46 | 1485.48 |
|  | COKMCCJN_03953 | COKMCCJN_03953 | 0.351 | -1.512 | 1.33E-03 | 8.38E-03 | down | 23.30 | 5.32 |
|  | COKMCCJN_03954 | COKMCCJN_03954 | 0.315 | -1.666 | 2.34E-07 | 6.27E-06 | down | 25.61 | 5.08 |
|  | COKMCCJN_03955 | COKMCCJN_03955 | 0.184 | -2.444 | 2.41E-13 | 2.95E-11 | down | 21.56 | 2.41 |
|  | COKMCCJN_03956 | *xerC* | 0.224 | -2.159 | 9.33E-12 | 8.26E-10 | down | 23.36 | 3.16 |
|  | COKMCCJN_03972 | COKMCCJN_03972 | 0.487 | -1.039 | 6.93E-03 | 3.03E-02 | down | 3.43 | 0.99 |
|  | COKMCCJN_03990 | *ybdG* | 0.458 | -1.126 | 3.90E-03 | 1.93E-02 | down | 4.21 | 1.18 |
|  | COKMCCJN_03992 | *manC1* | 0.299 | -1.740 | 1.81E-09 | 8.67E-08 | down | 14.17 | 2.66 |
|  | COKMCCJN_03993 | *algC* | 0.480 | -1.059 | 1.31E-03 | 8.26E-03 | down | 4.94 | 1.42 |
|  | COKMCCJN_04005 | *ctaE* | 3.523 | 1.817 | 1.98E-03 | 1.14E-02 | up | 0.62 | 1.32 |
|  | COKMCCJN_04012 | *ompL* | 0.095 | -3.399 | 1.90E-09 | 8.96E-08 | down | 21.45 | 1.14 |
|  | COKMCCJN_04013 | *dnaK* | 2.145 | 1.101 | 1.80E-03 | 1.06E-02 | up | 1.01 | 1.38 |
|  | COKMCCJN_04016 | COKMCCJN_04016 | 0.390 | -1.359 | 8.34E-04 | 5.82E-03 | down | 5.39 | 1.34 |
|  | COKMCCJN_04026 | *gap2* | 3.154 | 1.657 | 2.48E-05 | 3.35E-04 | up | 0.88 | 1.71 |
|  | COKMCCJN_04028 | *hcp* | 0.437 | -1.193 | 1.19E-03 | 7.73E-03 | down | 7.17 | 2.02 |
|  | COKMCCJN_04053 | *ttgB* | 2.710 | 1.438 | 1.46E-05 | 2.14E-04 | up | 1.43 | 2.56 |
|  | COKMCCJN_04055 | *oprM* | 3.747 | 1.906 | 3.43E-03 | 1.75E-02 | up | 0.11 | 0.28 |
|  | COKMCCJN_04058 | COKMCCJN_04058 | 2.333 | 1.222 | 1.19E-02 | 4.71E-02 | up | 1.70 | 2.46 |
|  | COKMCCJN_04060 | COKMCCJN_04060 | 0.312 | -1.681 | 7.91E-03 | 3.38E-02 | down | 4.43 | 0.91 |
|  | COKMCCJN_04084 | *nhaC* | 3.630 | 1.860 | 3.48E-05 | 4.46E-04 | up | 0.40 | 0.91 |
|  | COKMCCJN_04085 | *mhpD* | 3.903 | 1.965 | 3.30E-08 | 1.15E-06 | up | 1.57 | 3.89 |
|  | COKMCCJN_04090 | *sgrR* | 0.449 | -1.155 | 5.23E-03 | 2.42E-02 | down | 6.12 | 1.86 |
|  | COKMCCJN_04091 | *rimK* | 3.695 | 1.886 | 2.60E-04 | 2.26E-03 | up | 1.08 | 2.95 |
|  | COKMCCJN_04092 | COKMCCJN_04092 | 3.309 | 1.726 | 1.65E-03 | 9.89E-03 | up | 3.58 | 9.02 |
|  | COKMCCJN_04094 | COKMCCJN_04094 | 3.239 | 1.696 | 2.89E-04 | 2.46E-03 | up | 1.79 | 4.08 |
|  | COKMCCJN_04097 | COKMCCJN_04097 | 0.253 | -1.981 | 5.39E-07 | 1.29E-05 | down | 59.80 | 8.72 |
|  | COKMCCJN_04098 | *ugpC* | 0.244 | -2.034 | 5.44E-07 | 1.29E-05 | down | 161.95 | 22.80 |
|  | COKMCCJN_04099 | *ugpQ* | 0.288 | -1.798 | 3.20E-06 | 6.02E-05 | down | 69.67 | 11.85 |
|  | COKMCCJN_04109 | COKMCCJN_04109 | 2.062 | 1.044 | 1.12E-03 | 7.41E-03 | up | 2.95 | 4.01 |
|  | COKMCCJN_04111 | COKMCCJN_04111 | 3.837 | 1.940 | 1.45E-06 | 3.00E-05 | up | 2.03 | 4.77 |
|  | COKMCCJN_04113 | ***cat*** | 6.079 | 2.604 | 5.46E-13 | 6.31E-11 | up | 2.13 | 8.09 |
|  | COKMCCJN_04114 | ***phnP*** | 4.035 | 2.012 | 4.32E-09 | 1.85E-07 | up | 8.89 | 21.23 |
|  | COKMCCJN_04115 | ***phnN*** | 4.445 | 2.152 | 1.31E-09 | 6.63E-08 | up | 5.35 | 14.05 |
|  | COKMCCJN_04116 | ***phnM*** | 3.446 | 1.785 | 1.40E-08 | 5.36E-07 | up | 15.82 | 33.06 |
|  | COKMCCJN_04117 | ***phnL*** | 2.937 | 1.554 | 5.55E-06 | 9.39E-05 | up | 15.52 | 27.51 |
|  | COKMCCJN_04118 | ***phnK*** | 2.863 | 1.518 | 1.06E-05 | 1.62E-04 | up | 23.02 | 40.00 |
|  | COKMCCJN_04119 | ***phnJ*** | 2.641 | 1.401 | 2.29E-05 | 3.15E-04 | up | 25.43 | 39.63 |
|  | COKMCCJN_04120 | ***phnI*** | 2.448 | 1.292 | 1.85E-04 | 1.70E-03 | up | 7.21 | 11.06 |
|  | COKMCCJN_04121 | ***phnH*** | 1.924 | 0.944 | 1.78E-02 | 6.44E-02 | up | 7.59 | 9.15 |
|  | COKMCCJN_04122 | ***phnG*** | 1.680 | 0.749 | 8.20E-02 | 2.04E-01 | up | 8.84 | 9.44 |
|  | COKMCCJN_04123 | ***phnF*** | 1.656 | 0.727 | 7.83E-02 | 1.98E-01 | up | 15.14 | 15.54 |
|  | COKMCCJN_04124 | ***phnE*** | 2.184 | 1.127 | 5.01E-03 | 2.34E-02 | up | 3.44 | 4.56 |
|  | COKMCCJN_04125 | ***phnD*** | 2.624 | 1.392 | 2.54E-03 | 1.39E-02 | up | 6.27 | 10.59 |
|  | COKMCCJN_04126 | ***phnC*** | 2.603 | 1.380 | 1.33E-04 | 1.30E-03 | up | 16.71 | 27.31 |
|  | COKMCCJN_04130 | *tcyP* | 3.316 | 1.729 | 4.72E-05 | 5.68E-04 | up | 1.68 | 3.65 |
|  | COKMCCJN_04141 | COKMCCJN_04141 | 2.674 | 1.419 | 2.74E-04 | 2.36E-03 | up | 12.99 | 23.59 |
|  | COKMCCJN_04156 | *baiA* | 2.362 | 1.240 | 2.92E-03 | 1.54E-02 | up | 0.88 | 1.37 |
|  | COKMCCJN_04161 | *ompW* | 5.163 | 2.368 | 1.23E-07 | 3.65E-06 | up | 1.82 | 5.61 |
|  | COKMCCJN_04165 | *phnE* | 2.671 | 1.418 | 4.34E-03 | 2.09E-02 | up | 0.75 | 1.16 |
|  | COKMCCJN_04171 | *sasA* | 0.488 | -1.034 | 1.19E-02 | 4.71E-02 | down | 0.87 | 0.26 |
|  | COKMCCJN_04190 | COKMCCJN_04190 | 2.213 | 1.146 | 9.56E-03 | 3.94E-02 | up | 2.38 | 3.47 |
|  | COKMCCJN_04191 | COKMCCJN_04191 | 2.378 | 1.250 | 1.91E-05 | 2.69E-04 | up | 2.30 | 3.37 |
|  | COKMCCJN_04197 | COKMCCJN_04197 | 2.232 | 1.158 | 5.63E-03 | 2.57E-02 | up | 0.40 | 0.56 |
|  | COKMCCJN_04198 | *rhaR* | 0.358 | -1.482 | 8.37E-05 | 9.07E-04 | down | 10.44 | 2.26 |
|  | COKMCCJN_04199 | COKMCCJN_04199 | 11.360 | 3.506 | 9.60E-21 | 4.00E-18 | up | 0.49 | 3.53 |
|  | COKMCCJN_04200 | *manA* | 13.942 | 3.801 | 4.49E-20 | 1.70E-17 | up | 0.55 | 4.76 |
|  | COKMCCJN_04201 | *manP* | 13.883 | 3.795 | 3.91E-13 | 4.65E-11 | up | 0.64 | 5.72 |
|  | COKMCCJN_04202 | COKMCCJN_04202 | 9.853 | 3.301 | 2.18E-26 | 3.03E-23 | up | 1.79 | 10.96 |
|  | COKMCCJN_04230 | *ald1* | 9.754 | 3.286 | 3.17E-14 | 4.89E-12 | up | 1.49 | 8.96 |
|  | COKMCCJN_04231 | COKMCCJN_04231 | 55.477 | 5.794 | 1.68E-17 | 4.67E-15 | up | 2.92 | 112.06 |
|  | COKMCCJN_04232 | *acoR* | 4.250 | 2.087 | 3.20E-04 | 2.67E-03 | up | 0.37 | 0.89 |
|  | COKMCCJN_04248 | *yedJ* | 0.296 | -1.754 | 8.60E-08 | 2.71E-06 | down | 36.03 | 6.78 |
|  | COKMCCJN_04249 | COKMCCJN_04249 | 0.489 | -1.033 | 1.99E-03 | 1.14E-02 | down | 9.43 | 2.88 |
|  | COKMCCJN_04250 | COKMCCJN_04250 | 0.414 | -1.271 | 9.52E-04 | 6.47E-03 | down | 5.02 | 1.30 |
|  | COKMCCJN_04251 | COKMCCJN_04251 | 0.255 | -1.971 | 5.33E-04 | 3.96E-03 | down | 20.49 | 3.90 |
|  | COKMCCJN_04264 | COKMCCJN_04264 | 0.469 | -1.093 | 1.26E-02 | 4.91E-02 | down | 2.28 | 0.70 |
|  | COKMCCJN_04265 | *allA* | 0.201 | -2.313 | 2.60E-03 | 1.41E-02 | down | 1.36 | 0.17 |
|  | COKMCCJN_04266 | *alc* | 0.273 | -1.873 | 7.35E-04 | 5.27E-03 | down | 1.71 | 0.31 |
|  | COKMCCJN_04267 | *pucL* | 0.209 | -2.260 | 1.19E-04 | 1.22E-03 | down | 16.32 | 2.19 |
|  | COKMCCJN_04268 | COKMCCJN_04268 | 0.156 | -2.681 | 4.36E-05 | 5.34E-04 | down | 10.78 | 1.21 |
|  | COKMCCJN_04273 | COKMCCJN_04273 | 4.329 | 2.114 | 5.81E-04 | 4.26E-03 | up | 0.25 | 0.63 |
|  | COKMCCJN_04274 | COKMCCJN_04274 | 3.872 | 1.953 | 1.53E-03 | 9.30E-03 | up | 0.17 | 0.36 |
|  | COKMCCJN_04282 | *aceB* | 0.154 | -2.695 | 2.35E-09 | 1.10E-07 | down | 6.76 | 0.70 |
|  | COKMCCJN_04283 | COKMCCJN_04283 | 0.217 | -2.207 | 3.25E-06 | 6.07E-05 | down | 15.39 | 2.18 |
|  | COKMCCJN_04294 | *yejF* | 0.201 | -2.316 | 1.84E-04 | 1.69E-03 | down | 50.40 | 5.12 |
|  | COKMCCJN_04306 | *lamB* | 2.530 | 1.339 | 4.65E-04 | 3.55E-03 | up | 0.84 | 1.36 |
|  | COKMCCJN_04308 | *dmlR* | 0.402 | -1.316 | 3.73E-03 | 1.87E-02 | down | 5.46 | 1.54 |
|  | COKMCCJN_04309 | *lldP* | 3.167 | 1.663 | 3.22E-04 | 2.68E-03 | up | 0.70 | 1.55 |
|  | COKMCCJN_04319 | *lysN* | 0.448 | -1.159 | 6.60E-03 | 2.92E-02 | down | 8.28 | 2.55 |
|  | COKMCCJN_04322 | *glpE* | 2.669 | 1.416 | 3.77E-03 | 1.88E-02 | up | 2.46 | 3.60 |
|  | COKMCCJN_04323 | COKMCCJN_04323 | 0.467 | -1.100 | 2.37E-03 | 1.32E-02 | down | 988.46 | 279.41 |
|  | COKMCCJN_04337 | *chiA* | 4.054 | 2.020 | 1.21E-08 | 4.70E-07 | up | 1.06 | 2.61 |
|  | COKMCCJN_04338 | COKMCCJN_04338 | 3.147 | 1.654 | 9.84E-04 | 6.66E-03 | up | 0.46 | 0.89 |
|  | COKMCCJN_04340 | COKMCCJN_04340 | 0.411 | -1.281 | 4.51E-03 | 2.15E-02 | down | 1.70 | 0.43 |
|  | COKMCCJN_04342 | *rclR* | 4.192 | 2.067 | 2.33E-04 | 2.07E-03 | up | 4.84 | 13.98 |
|  | COKMCCJN_04365 | *yibL* | 2.410 | 1.269 | 3.88E-05 | 4.84E-04 | up | 12.20 | 17.91 |
|  | COKMCCJN_04369 | *rpoE* | 2.391 | 1.258 | 5.91E-03 | 2.67E-02 | up | 128.76 | 197.58 |
|  | COKMCCJN_04378 | COKMCCJN_04378 | 3.670 | 1.876 | 7.21E-06 | 1.18E-04 | up | 9.07 | 21.33 |
|  | COKMCCJN_04395 | COKMCCJN_04395 | 0.231 | -2.115 | 5.67E-06 | 9.55E-05 | down | 6.77 | 1.06 |
|  | COKMCCJN_04400 | COKMCCJN_04400 | 2.779 | 1.474 | 3.25E-04 | 2.70E-03 | up | 1.01 | 1.86 |
|  | COKMCCJN_04404 | COKMCCJN_04404 | 0.376 | -1.411 | 2.91E-05 | 3.84E-04 | down | 5.18 | 1.17 |
|  | COKMCCJN_04412 | *dapE* | 2.477 | 1.309 | 7.71E-05 | 8.45E-04 | up | 1.11 | 1.73 |
|  | COKMCCJN_04413 | *garR* | 0.471 | -1.088 | 1.30E-03 | 8.24E-03 | down | 9.56 | 2.76 |
|  | COKMCCJN_04437 | COKMCCJN_04437 | 0.322 | -1.635 | 1.38E-04 | 1.35E-03 | down | 6.92 | 1.42 |
|  | COKMCCJN_04438 | *btuD* | 0.282 | -1.827 | 2.96E-05 | 3.90E-04 | down | 53.78 | 10.09 |
|  | COKMCCJN_04439 | COKMCCJN_04439 | 0.327 | -1.612 | 1.23E-03 | 7.92E-03 | down | 4.16 | 0.90 |
|  | COKMCCJN_04457 | COKMCCJN_04457 | 0.385 | -1.377 | 5.23E-05 | 6.15E-04 | down | 53.99 | 13.37 |
|  | COKMCCJN_04458 | *parA* | 0.453 | -1.142 | 9.33E-03 | 3.85E-02 | down | 201.77 | 63.20 |
|  | COKMCCJN_04459 | COKMCCJN_04459 | 0.488 | -1.036 | 1.06E-02 | 4.27E-02 | down | 39.81 | 12.71 |
|  | COKMCCJN_04464 | COKMCCJN_04464 | 0.481 | -1.057 | 2.13E-03 | 1.21E-02 | down | 6.92 | 2.07 |
|  | COKMCCJN_04467 | *dctB* | 0.257 | -1.962 | 1.98E-07 | 5.42E-06 | down | 8.52 | 1.27 |
|  | COKMCCJN_04469 | COKMCCJN_04469 | 0.456 | -1.134 | 3.85E-03 | 1.91E-02 | down | 3.96 | 1.12 |
|  | COKMCCJN_04475 | *putP* | 2.608 | 1.383 | 6.55E-07 | 1.50E-05 | up | 8.03 | 12.88 |
|  | COKMCCJN_04476 | COKMCCJN_04476 | 2.762 | 1.466 | 2.35E-04 | 2.07E-03 | up | 9.87 | 17.07 |
|  | COKMCCJN_04481 | COKMCCJN_04481 | 2.667 | 1.415 | 1.25E-02 | 4.88E-02 | up | 14.15 | 27.09 |
|  | COKMCCJN_04487 | COKMCCJN_04487 | 0.390 | -1.360 | 4.57E-05 | 5.53E-04 | down | 138.89 | 35.82 |

FC, fold change. TPM, transcripts per million reads.

**Table S6. Taxonomic information of sequences retrieved by the primers phnJ-620F_895R and phnL-370F_672R from ZQ microbial community.** *Vibrio* phnJ/phnL OTUs were assigned into phn operon clusters labelled with bold letters.

| **Domain** | **Order** | **Species** | ***phnJ*-OTUs-Sum** | **Seq-Sum** | **Seq-Percentage %** |
| --- | --- | --- | --- | --- | --- |
| **Bacteria** | *Burkholderiales* | *Variovorax paradoxus* | 1 | 4 | 0.004 |
|  | *Desulfovibrionales* | *Desulfovibrio piger* | 1 | 4 | 0.004 |
|  | *Enterobacterales* | *Citrobacter freundii* | 1 | 4 | 0.004 |
|  | *Enterobacterales* | *Citrobacter portucalensis* | 1 | 2 | 0.002 |
|  | *Enterobacterales* | *Enterobacter cancerogenus* | 1 | 2 | 0.002 |
|  | *Enterobacterales* | *Enterobacter roggenkampii* | 1 | 2 | 0.002 |
|  | *Enterobacterales* | *Escherichia coli* | 3 | 108 | 0.108 |
|  | *Enterobacterales* | *Pantoea* sp. MSR2 | 1 | 4 | 0.004 |
|  | *Enterobacterales* | *Serratia marcescens* | 1 | 4 | 0.004 |
|  | *Enterobacterales* | *Serratia plymuthica* | 1 | 2 | 0.002 |
|  | *Neisseriales* | *Chromobacterium vaccinii* | 1 | 27722 | 27.682 |
|  | *Pseudomonadales* | *Pseudomonas stutzeri* | 1 | 2 | 0.002 |
|  | *Rhizobiales* | *Agrobacterium tumefaciens* | 2 | 49 | 0.049 |
|  | *Rhizobiales* | *Blastochloris viridis* | 1 | 3 | 0.003 |
|  | *Rhizobiales* | *Bradyrhizobium cosmicum* | 1 | 3 | 0.003 |
|  | *Rhizobiales* | *Bradyrhizobium* sp. CCGE-LA001 | 1 | 5 | 0.005 |
|  | *Rhizobiales* | *Neorhizobium galegae* | 1 | 6 | 0.006 |
|  | *Rhizobiales* | *Rhizobium daejeonense* | 1 | 8 | 0.008 |
|  | *Rhizobiales* | *Rhizobium grahamii* | 1 | 10 | 0.010 |
|  | *Rhizobiales* | *Rhizobium oryzihabitans* | 1 | 3 | 0.003 |
|  | *Rhizobiales* | *Rhizobium pusense* | 1 | 2 | 0.002 |
|  | *Rhizobiales* | *Sinorhizobium americanum* | 1 | 6 | 0.006 |
|  | *Rhizobiales* | *Sinorhizobium meliloti* | 1 | 2 | 0.002 |
|  | *Rhizobiales* | *Xanthobacter autotrophicus* | 2 | 190 | 0.190 |
|  | *Rhodobacterales* | *Celeribacter baekdonensis* | 1 | 3 | 0.003 |
|  | *Rhodobacterales* | *Celeribacter indicus* | 3 | 5063 | 5.056 |
|  | *Rhodobacterales* | *Defluviimonas alba* | 1 | 16 | 0.016 |
|  | *Rhodobacterales* | *Labrenzia* sp. PHM005 | 1 | 3 | 0.003 |
|  | *Rhodobacterales* | *Leisingera aquaemixtae* | 4 | 29 | 0.029 |
|  | *Rhodobacterales* | *Leisingera* sp. NJS201 | 3 | 121 | 0.121 |
|  | *Rhodobacterales* | *Litoreibacter* sp. LN3S51 | 1 | 205 | 0.205 |
|  | *Rhodobacterales* | *Maribius* sp. THAF1 | 2 | 64 | 0.064 |
|  | *Rhodobacterales* | *Marivivens* sp. JLT3646 | 1 | 4 | 0.004 |
|  | *Rhodobacterales* | *Oceanicola* sp. D3 | 1 | 5 | 0.005 |
|  | *Rhodobacterales* | *Octadecabacter arcticus* | 1 | 11 | 0.011 |
|  | *Rhodobacterales* | *Octadecabacter temperatus* | 2 | 14 | 0.014 |
|  | *Rhodobacterales* | *Paracoccus aminophilus* | 1 | 29 | 0.029 |
|  | *Rhodobacterales* | *Paracoccus liaowanqingii* | 1 | 11 | 0.011 |
|  | *Rhodobacterales* | *Paracoccus* sp. Arc7-R13 | 2 | 7 | 0.007 |
|  | *Rhodobacterales* | *Phaeobacter gallaeciensis* | 1 | 125 | 0.125 |
|  | *Rhodobacterales* | *Phaeobacter inhibens* | 1 | 7 | 0.007 |
|  | *Rhodobacterales* | *Phaeobacter porticola* | 1 | 2 | 0.002 |
|  | *Rhodobacterales* | *Pseudorhodobacter* sp. S12M18 | 1 | 460 | 0.459 |
|  | *Rhodobacterales* | *Rhodobacteraceae* bacterium | 5 | 155 | 0.155 |
|  | *Rhodobacterales* | *Rhodobacteraceae* bacterium QY30 | 3 | 35 | 0.035 |
|  | *Rhodobacterales* | *Rhodobacteraceae* bacterium SC52 | 1 | 20 | 0.020 |
|  | *Rhodobacterales* | *Rhodovulum* sp. P5 | 4 | 602 | 0.601 |
|  | *Rhodobacterales* | *Roseibacterium elongatum* | 2 | 1663 | 1.661 |
|  | *Rhodobacterales* | *Roseobacter litoralis* | 1 | 251 | 0.251 |
|  | *Rhodobacterales* | *Roseovarius indicus* | 3 | 29 | 0.029 |
|  | *Rhodobacterales* | *Roseovarius mucosus* | 1 | 374 | 0.373 |
|  | *Rhodobacterales* | *Roseovarius* sp. THAF8 | 2 | 8 | 0.008 |
|  | *Rhodobacterales* | *Roseovarius* sp. THAF9 | 1 | 2 | 0.002 |
|  | *Rhodobacterales* | *Ruegeria pomeroyi* | 2 | 17 | 0.017 |
|  | *Rhodobacterales* | *Ruegeria* sp. THAF33 | 4 | 79 | 0.079 |
|  | *Rhodobacterales* | *Sagittula* sp. P11 | 1 | 3 | 0.003 |
|  | *Rhodobacterales* | *Salipiger profundus* | 1 | 3 | 0.003 |
|  | *Rhodobacterales* | *Sulfitobacter* sp. AM1-D1 | 1 | 56 | 0.056 |
|  | *Rhodobacterales* | *Sulfitobacter* sp. BSw21498 | 1 | 8 | 0.008 |
|  | *Rhodobacterales* | *Sulfitobacter* sp. D7 | 4 | 703 | 0.702 |
|  | *Rhodobacterales* | *Sulfitobacter* sp. SK025 | 3 | 42 | 0.042 |
|  | *Rhodobacterales* | *Thalassobius gelatinovorus* | 1 | 78 | 0.078 |
|  | *Rhodobacterales* | *Thalassococcus* sp. S3 | 1 | 2 | 0.002 |
|  | *Rhodobacterales* | *Yangia pacifica* | 4 | 568 | 0.567 |
|  | *Rhodobacterales* | *Yoonia vestfoldensis* | 2 | 141 | 0.141 |
|  | unclassified Bacteria | uncultured bacterium APKI441 | 2 | 63 | 0.063 |
|  | unclassified Bacteria | uncultured bacterium B3TF MPn2 | 2 | 3845 | 3.839 |
|  | unclassified taxon | unclassified taxon | 37 | 56938 | 56.856 |
|  | *Vibrionales* | ***Vibrio gigantis* (Cluster I)** | 1 | 133 | 0.133 |
| **Domain** | **Order** | **Species** | ***phnL*-OTUs-Sum** | **Seq-Sum** | **Seq-Percentage%** |
| **Bacteria** | *Aeromonadales* | *Aeromonas hydrophila* | 1 | 46 | 0.056 |
|  | *Aeromonadales* | *Aeromonas* sp. 1805 | 1 | 3 | 0.004 |
|  | *Aeromonadales* | *Aeromonas veronii* | 1 | 11 | 0.013 |
|  | *Alteromonadales* | *Agarivorans gilvus* | 1 | 22 | 0.027 |
|  | *Bacteroidales* | *Alistipes shahii* | 1 | 16 | 0.020 |
|  | *Bacteroidales* | *Bacteroides fragilis* | 1 | 7 | 0.009 |
|  | *Bacteroidales* | *Bacteroides thetaiotaomicron* | 1 | 11 | 0.013 |
|  | *Bacteroidales* | *Prevotella denticola* | 1 | 15 | 0.018 |
|  | *Bdellovibrionales* | *Bdellovibrio bacteriovorus* | 1 | 6 | 0.007 |
|  | *Bifidobacteriales* | *Bifidobacterium catenulatum* | 1 | 16 | 0.020 |
|  | *Burkholderiales* | *Achromobacter xylosoxidans* | 1 | 5 | 0.006 |
|  | *Burkholderiales* | *Burkholderia ubonensis* | 1 | 42 | 0.051 |
|  | *Burkholderiales* | *Cupriavidus malaysiensis* | 1 | 71 | 0.087 |
|  | *Cellvibrionales* | *Kineobactrum* sp. M2 | 1 | 5 | 0.006 |
|  | *Cellvibrionales* | *Microbulbifer hydrolyticus* | 1 | 12 | 0.015 |
|  | *Cellvibrionales* | *Microbulbifer* sp. SH-1 | 1 | 16 | 0.020 |
|  | *Chromatiales* | *Marichromatium purpuratum* | 1 | 6 | 0.007 |
|  | *Clostridiales* | *Coprococcus* sp. ART55/1 | 1 | 38 | 0.046 |
|  | *Desulfobacterales* | *Desulfobacter hydrogenophilus* | 3 | 45 | 0.055 |
|  | *Desulfobacterales* | *Desulfosarcina widdelii* | 1 | 3 | 0.004 |
|  | *Desulfovibrionales* | *Desulfomicrobium baculatum* | 1 | 3 | 0.004 |
|  | *Enterobacterales* | *Cedecea neteri* | 1 | 89 | 0.109 |
|  | *Enterobacterales* | *Enterobacter cloacae* | 1 | 5 | 0.006 |
|  | *Enterobacterales* | *Enterobacter kobei* | 1 | 36 | 0.044 |
|  | *Enterobacterales* | *Enterobacter* sp. E20 | 1 | 11 | 0.013 |
|  | *Enterobacterales* | *Escherichia coli* | 9 | 618 | 0.754 |
|  | *Enterobacterales* | *Kluyvera georgiana* | 1 | 116 | 0.141 |
|  | *Enterobacterales* | *Kluyvera intermedia* | 2 | 5 | 0.006 |
|  | *Enterobacterales* | *Kosakonia oryzae* | 2 | 20 | 0.024 |
|  | *Enterobacterales* | *Leclercia adecarboxylata* | 1 | 47 | 0.057 |
|  | *Enterobacterales* | *Leclercia* sp. W6 | 1 | 39 | 0.048 |
|  | *Enterobacterales* | *Pantoea* sp. At-9b | 1 | 7 | 0.009 |
|  | *Enterobacterales* | *Pantoea vagans* | 1 | 6 | 0.007 |
|  | *Enterobacterales* | *Raoultella ornithinolytica* | 1 | 18 | 0.022 |
|  | *Enterobacterales* | *Serratia* sp. FGI94 | 1 | 97 | 0.118 |
|  | *Enterobacterales* | *Yersinia enterocolitica* | 1 | 15 | 0.018 |
|  | *Enterobacterales* | *Yersinia entomophaga* | 1 | 3 | 0.004 |
|  | *Enterobacterales* | *Yersinia intermedia* | 1 | 94 | 0.115 |
|  | *Flavobacteriales* | *Chryseobacterium* sp. H3001 | 1 | 4 | 0.005 |
|  | *Hydrogenophilales* | *Hydrogenophilus thermoluteolus* | 2 | 314 | 0.383 |
|  | *Magnetococcales* | Candidatus *Magnetococcus massalia* | 1 | 7 | 0.009 |
|  | *Nitrosomonadales* | *Sterolibacterium denitrificans* | 1 | 3 | 0.004 |
|  | *Nostocales* | *Cylindrospermum* sp. NIES-4074 | 1 | 3 | 0.004 |
|  | *Oceanospirillales* | *Halomonas* sp. BC-M4-5 | 1 | 28 | 0.034 |
|  | *Oceanospirillales* | *Marinobacterium aestuarii* | 1 | 233 | 0.284 |
|  | *Pseudomonadales* | *Acinetobacter* sp. ACNIH2 | 1 | 2 | 0.002 |
|  | *Pseudomonadales* | *Pseudomonas aeruginosa* | 2 | 1585 | 1.933 |
|  | *Pseudomonadales* | *Pseudomonas asplenii* | 1 | 223 | 0.272 |
|  | *Pseudomonadales* | *Pseudomonas* sp. CC6-YY-74 | 1 | 15 | 0.018 |
|  | *Pseudomonadales* | *Pseudomonas stutzeri* | 1 | 4 | 0.005 |
|  | *Pseudomonadales* | *Pseudomonas vancouverensis* | 1 | 63 | 0.077 |
|  | *Rhizobiales* | *Aminobacter aminovorans* | 1 | 3 | 0.004 |
|  | *Rhizobiales* | *Bosea* sp. Tri-49 | 1 | 15 | 0.018 |
|  | *Rhizobiales* | *Bradyrhizobium icense* | 1 | 72 | 0.088 |
|  | *Rhizobiales* | *Bradyrhizobium paxllaeri* | 1 | 35 | 0.043 |
|  | *Rhizobiales* | *Bradyrhizobium* sp. ORS 285 | 1 | 1455 | 1.774 |
|  | *Rhizobiales* | *Mesorhizobium* sp. M4B.F.Ca.ET.058.02.1.1 | 1 | 8 | 0.010 |
|  | *Rhizobiales* | *Pseudorhodoplanes sinuspersici* | 1 | 20 | 0.024 |
|  | *Rhizobiales* | *Variibacter gotjawalensis* | 1 | 12 | 0.015 |
|  | *Rhodobacterales* | *Antarctobacter heliothermus* | 1 | 4 | 0.005 |
|  | *Rhodobacterales* | *Celeribacter baekdonensis* | 1 | 5 | 0.006 |
|  | *Rhodobacterales* | *Celeribacter marinus* | 1 | 92 | 0.112 |
|  | *Rhodobacterales* | *Confluentimicrobium* sp. EMB200-NS6 | 1 | 4 | 0.005 |
|  | *Rhodobacterales* | *Leisingera aquaemixtae* | 1 | 4 | 0.005 |
|  | *Rhodobacterales* | *Phaeobacter inhibens* | 2 | 22 | 0.027 |
|  | *Rhodobacterales* | *Rhodobacter blasticus* | 1 | 15 | 0.018 |
|  | *Rhodobacterales* | *Rhodobacter sphaeroides* | 1 | 16 | 0.020 |
|  | *Rhodobacterales* | *Rhodobacteraceae* bacterium SC52 | 1 | 119 | 0.145 |
|  | *Rhodobacterales* | *Roseivivax* sp. THAF40 | 2 | 13 | 0.016 |
|  | *Rhodobacterales* | *Ruegeria pomeroyi* | 1 | 66 | 0.080 |
|  | *Rhodobacterales* | *Sagittula* sp. P11 | 1 | 139 | 0.170 |
|  | *Rhodobacterales* | *Sulfitobacter* sp. BSw21498 | 1 | 2 | 0.002 |
|  | *Rhodobacterales* | *Thioclava nitratireducens* | 1 | 3 | 0.004 |
|  | *Rhodobacterales* | *Yangia pacifica* | 1 | 13 | 0.016 |
|  | *Rhodobacterales* | *Yoonia vestfoldensis* | 1 | 4 | 0.005 |
|  | *Rhodocyclales* | *Azoarcus communis* | 1 | 2 | 0.002 |
|  | *Rhodospirillales* | *Gluconobacter oxydans* | 1 | 48 | 0.059 |
|  | *Sphingomonadales* | *Altererythrobacter dongtanensis* | 1 | 57 | 0.070 |
|  | *Sphingomonadales* | *Altererythrobacter ishigakiensis* | 1 | 10524 | 12.834 |
|  | *Sphingomonadales* | *Altererythrobacter* sp. BO-6 | 1 | 6 | 0.007 |
|  | *Sphingomonadales* | *Erythrobacter* sp. Alg231-14 | 1 | 3 | 0.004 |
|  | *Sphingomonadales* | *Sphingopyxis* sp. LPB0140 | 1 | 16 | 0.020 |
|  | *Synechococcales* | *Synechococcus* sp. CC9605 | 4 | 116 | 0.141 |
|  | *Synechococcales* | *Synechococcus* sp. KORDI-52 | 1 | 4 | 0.005 |
|  | *Thiotrichales* | *Thiomicrospira cyclica* | 1 | 11 | 0.013 |
|  | unclassified Bacteria | uncultured bacterium GRIST10 | 3 | 223 | 0.272 |
|  | unclassified *Flavobacteriia* | uncultured *Flavobacteriia* bacterium | 1 | 14 | 0.017 |
|  | unclassified *Gammaproteobacteria* | uncultured gamma proteobacterium HF0010 05D02 | 1 | 5 | 0.006 |
|  | unclassified *Planctomycetes* | *Planctomycetes* bacterium V144 | 1 | 6 | 0.007 |
|  | *Verrucomicrobiales* | *Luteolibacter* sp. G-1-1-1 | 1 | 23 | 0.028 |
|  | *Vibrionales* | ***Vibrio brasiliensis* (Cluster III)** | 2 | 383 | 0.467 |
|  | *Vibrionales* | ***Vibrio casei* (Cluster V)** | 1 | 13699 | 16.705 |
|  | *Vibrionales* | ***Vibrio chagasii* (Cluster I)** | 8 | 8280 | 10.097 |
|  | *Vibrionales* | ***Vibrio coralliirubri* (Cluster I)** | 2 | 23 | 0.028 |
|  | *Vibrionales* | ***Vibrio gigantis* (Cluster I)** | 4 | 1428 | 1.741 |
|  | *Vibrionales* | ***Vibrio mediterranei* (Cluster II)** | 12 | 15140 | 18.463 |
|  | *Vibrionales* | ***Vibrio nigripulchritudo* (Cluster VII)** | 4 | 1675 | 2.043 |
|  | *Vibrionales* | ***Vibrio rumoiensis* (Cluster V)** | 1 | 153 | 0.187 |
|  | *Vibrionales* | ***Vibrio_maritimus* (Cluster II)** | 6 | 218 | 0.266 |
| **Viruses** | *Caudovirales* | *Roseobacter* phage CRP-7 | 1 | 6 | 0.007 |
|  | *Caudovirales* | *Synechococcus* virus SRIP1 | 1 | 2 | 0.002 |
|  | *Caudovirales* | uncultured Mediterranean phage uvMED | 1 | 4 | 0.005 |
| **Eukaryota** | *Ustilaginales* | *Sporisorium scitamineum* | 1 | 25 | 0.030 |
|  | unclassified taxon | uncultured marine organism | 291 | 23660 | 28.852 |

**Table S7. The phnJ and phnL relative abundance (´10-4/recA) of Vibrionales spp. from Tara Ocean samples.**

DCM, deep chlorophyll maximum layer; SRF, surface water layer; MIX, marine epipelagic mixed layer; MES, mesopelagic zone. The suffixes present the sample fractions, such as 0.22-3 means the sample was collected at the fraction size between 0.22 μm and 3 μm.
